# Trajectories of physical function, depressive symptoms, and cognitive performance before and after falls in older adults: A matched multicohort study

**DOI:** 10.1371/journal.pmed.1005083

**Published:** 2026-08-03

**Authors:** Junjie Lin, Mika Kivimäki, Zifan Zhang, Hui Wang, Yangyang Cheng, Xiaolin Xu

**Affiliations:** 1 School of Public Health, The Second Affiliated Hospital, Zhejiang University School of Medicine, Hangzhou, Zhejiang, China; 2 The Key Laboratory of Intelligent Preventive Medicine of Zhejiang Province, Zhejiang University, Hangzhou, Zhejiang, China; 3 Division of Brain Sciences, University College London, London, United Kingdom; 4 Clinicum, University of Helsinki, Helsinki, Finland; 5 School of Public Health, Faculty of Medicine, The University of Queensland, Brisbane, Australia; University of melbourne, AUSTRALIA

## Abstract

**Background:**

Impairments in physical, psychological, and cognitive functioning are associated with an increased risk of falls. However, how these functional domains change longitudinally before and after fall events remains unclear. We examined individual and joint trajectories of these outcomes preceding and following falls.

**Methods and findings:**

Using data collected between 2010 and 2021 from three national cohorts in China, the UK, and the USA, we included 28,773 adults aged ≥60 years and performed 1:1 matching between participants who reported falls during follow-up and those who did not. Physical function, depressive symptoms, and cognitive performance were assessed repeatedly using measures of activities of daily living, standardized depression scales, and cognitive test performance, respectively. Falls were self-reported, and fall-related characteristics were evaluated by the need for medical attention, the number of falls, and hip fracture. Statistical analyses included piecewise linear mixed-effects models and group-based multi-trajectory models, with adjustment for age, sex, cohort, socioeconomic status, lifestyle factors, sensory function, and major chronic conditions. A total of 10,990 matched participants who experienced falls and those who did not were included (5,495:5,495; mean age 71.0 [SD 7.2]; 5,010 women and 5,980 men). Compared with participants who did not experience falls, those who experienced falls had poorer physical function, more severe depressive symptoms, and poorer cognitive performance at the time of the fall; these differences were apparent up to 5 years before a fall event and persisted thereafter. Participants who experienced falls also exhibited faster worsening of physical function both before (β_fall * pre-time_ −0.116, 95% CI [−0.142, −0.089]) and after the fall event (β_fall * post-time_ −0.039, 95% CI [−0.050, −0.028]). Faster worsening of depressive symptoms was observed only before the fall event (β_fall * pre-time_ −0.018, 95% CI [−0.026, −0.011]), whereas faster decline in cognitive performance was observed only thereafter (β_fall * post-time_ −0.008, 95% CI [−0.016, 0.000]). In joint trajectory analyses, participants who experienced falls were more likely to experience progressive multidomain functional decline, with the highest odds ratio (2.45, 95% CI [1.97, 3.05]) observed for concurrent worsening across all three domains. Associations were stronger among participants who experienced more severe falls. The main limitations of this study were the reliance on self-reported falls and some functional measures, and the observational design, which precluded causal inference.

**Conclusions:**

In older adults, multidomain functional decline appears to precede falls by several years and worsens further after fall events. Falls may therefore signal broader functional deterioration and highlight the need for prevention and management strategies addressing physical, psychological, and cognitive domains.

## Author summary

**Why was this study done?:**

- Physical, psychological, and cognitive impairments are bidirectionally associated with falls.
- It is unclear how these domains of functioning evolve separately and jointly before and after a fall event, or the extent to which functional changes are associated with fall severity.
- A more nuanced understanding of trajectories across multiple functional domains around fall events is needed to identify opportunities for timely multifactorial fall prevention and to inform targeted management strategies.

**What did the researchers do and find?:**

- Using a rigorous matched design to compare participants who experienced falls and those who did not in three national cohorts from the Program on Global Ageing, Health and Policy, this study examined separate and concurrent changes in physical function, depressive symptoms, and cognitive performance before and after falls.
- Older adults who experienced falls had greater physical function and more severe depressive symptoms, and poorer cognitive performance at the time of the fall, with differences evident up to 5 years beforehand and persisting thereafter. They also showed faster worsening of physical function both before and after the fall event, faster worsening of depressive symptoms before the fall event, and faster decline in cognitive performance after the fall event.
- The strongest association with falls was observed for concurrent worsening trajectories across all three functional domains, and these associations were stronger among participants who experienced more severe falls.

**What do these findings mean?:**

- Multidomain functional decline beginning years before falls and continuing after falls suggests that falls mark broader functional deterioration. This underscores the importance of preventing functional decline across physical, psychological and cognitive domains throughout the continuum of care—from primary to tertiary prevention—and of treating falls as a signal for intensified assessment and intervention.
- These findings support the World Guidelines for Falls Prevention and Management for Older Adults, which emphasize multidimensional and risk-stratified prevention.
- These findings should be interpreted in light of potential reporting bias arising from self-reported falls and some functional measures, as well as the inability to infer causality from the observational design.

## Abstract 3

In this multicohort study, Junjie Lin and colleagues show that multidomain functional decline appears to precede falls by several years and further worsens after fall events in older adults.

## Introduction

For decades, falls have been the second leading cause of unintentional injury-related deaths worldwide [1,2]. They pose an increasingly serious public health challenge among adults aged over 60 years, not only causing an estimated 684,000 deaths globally each year but also contributing to substantial immediate and long-term health burdens [2]. Studies indicate that, among older adults who experienced a fall, 23.5% used health services, 17.2% received treatment, and more than 15% experienced declines in social and physical activities [3,4]; falls also account for approximately 95% of hip fractures [4]. The World Health Organization *Step Safely* framework emphasizes that falls and fall-related injuries arise from interacting biological, behavioral, environmental, and socioeconomic risk factors [2]. At the individual-level, these risk factors include low mobility, depressive symptoms, and cognitive impairment [2,5]. Addressing these factors may help reduce the risk of falls and mitigate their adverse consequences [4,6]. When falls do occur, comprehensive management is recommended to improve long-term outcomes [2,6].

Much of the existing research has examined activities of daily living (ADL), depressive symptoms, and cognitive impairment either as risk factors for falls or as consequences of falls [5], often using longitudinal time-to-event analyses [7–10]. In a Swedish cohort study of community-dwelling adults aged ≥60 years, poorer physical and cognitive function were associated with an increased risk of falls [7]. Conversely, in a study of 9,816 older Korean adults, individuals with a history of falls had poorer ADL and cognitive performance than those without such a history [9]. In addition, the National Health and Aging Trends Study reported bidirectional relationships between falls and depressive symptoms [10]. Emerging studies have examined functional trajectories after a fall, either among individuals who experienced falls alone or by comparing their trajectories with those of individuals who did not experience falls [11,12].

However, few studies have examined physical function, depressive symptoms, and cognitive performance in relation to falls to describe how these key domains evolve in the periods before and after fall events, while comprehensively considering fall severity and related characteristics. This is an important limitation given the availability of interventions aimed at preventing multidomain functional decline in older adults and reducing the risk of recurrent falls, hip fractures, and mortality after a fall [2,13–15]. A more nuanced understanding of trajectories across multiple functional domains around fall events, with consideration of fall severity, is therefore needed to identify opportunities for timely multifactorial fall prevention and to inform targeted management strategies.

To fill these gaps, we used harmonized data from participants with and without falls aged ≥60 years from three national cohort studies, with the following aims: (1) to delineate longitudinal trajectories of physical function, depressive symptoms, and cognitive performance leading up to and following falls, compared with those in a matched nonfall group over a comparable time period; (2) to identify co-evolving patterns across these three functional domains throughout follow-up; and (3) to determine whether these trajectories varied according to fall severity as well as demographic and socioeconomic characteristics.

## Methods

### Study design and participants

This population-based, cross-national study used individual participant data from community-based samples in three sister cohorts in the Global Aging, Health, and Policy programme: the China Health and Retirement Longitudinal Study (CHARLS; China), the English Longitudinal Study of Ageing (ELSA; England), and the Health and Retirement Study (HRS; United States). These cohorts were selected because, among all HRS-family cohorts, they provided more than 10 years of follow-up, comparable fall-related assessments, and harmonizable measures of physical function, depressive symptoms, and cognitive performance. All these cohorts follow harmonized study protocols and conduct surveys every 2–3 years, enabling cross-regional comparisons and data pooling [16]. Details of the three cohorts’ designs are available elsewhere [17–19]. This study adheres to the Strengthening the Reporting of Observational Studies in Epidemiology guidelines (S1 Checklist).

**Fig 1:**
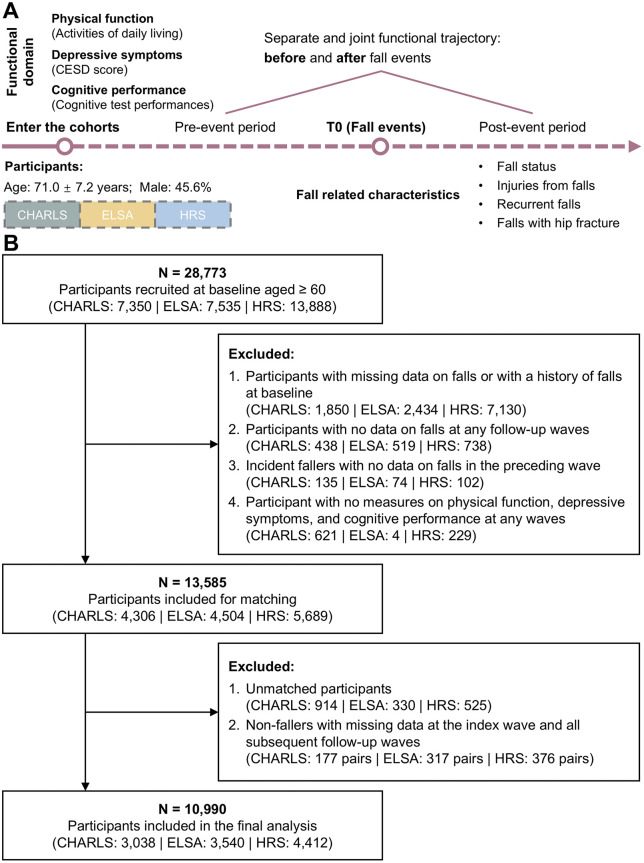
Study design and flow chart of the population selection process. **(A)** Diagram of the study design; **(B)** Population selection process. Abbreviations: CHARLS, China Health and Retirement Longitudinal Study; ELSA, English Longitudinal Study of Ageing; HRS, Health and Retirement Study; CESD, Center for Epidemiologic Studies Depression Scale.

Waves 1–5 of CHARLS (2011–2020), 5–9 of ELSA (2010–2019), and 11–15 of HRS (2012–2021) were used in the present analysis to harmonize study years between surveys. We included participants aged ≥60 years in ELSA and those aged ≥65 years in HRS, as fall-related questions were available only for these age groups in the respective cohorts. In CHARLS, fall-related questions were asked of all participants. To ensure comparability across cohorts and maintain focus on the target population, we included CHARLS participants aged ≥60 years. In total, 28,773 participants met the age eligibility criterion. The study design is visualized in Fig 1A.

The four additional inclusion criteria were (1) no history of falls at baseline; (2) available information on fall status during follow-up; (3) available data on physical function, depressive symptoms, and cognitive performance in at least one wave; and (4) among those who reported falls, fall status information available in the preceding wave to ensure identification of a recent incident fall. At this stage, 13,585 eligible participants were retained for matching (Fig 1B).

### Fall status and matching

In all studies, fall status was assessed using self-administered questionnaires. Participants were asked at each study wave whether they had fallen since the last wave. Participants who reported falls in at least one wave were categorized as the “falls” group, whereas those who reported no falls across all available waves were categorized as the “no falls” group.

Then, we performed 1:1 matching between participants who reported falls during follow-up and those who did not based on age (±2 years) and sex within each cohort. We defined time 0 (t0) as the wave at which the participant with falls first reported a fall, and the corresponding date was assigned as t0 for the matched no-fall participant (Fig 1A). To minimize misclassification bias, we further excluded matched pairs in which the no-fall participant had no available fall status data in any wave between t0 and the last follow-up. This resulted in a total of 10,990 participants for the analysis (Fig 1B).

To assess fall severity and fall-related characteristics, we constructed three alternative indicators: falls with injuries, recurrent falls, and falls with hip fracture. Falls with injuries were classified as: no falls; falls not requiring medical attention at t0 and no subsequent fall requiring medical attention during follow-up; and falls with injuries requiring medical attention at t0. Recurrent falls (available in ELSA and HRS) were classified as: no falls; a single fall (defined as a single fall at t0 and no multiple falls during subsequent follow-up); and multiple falls at t0. Falls with hip fracture were classified as: no falls; falls at t0 with no hip fracture history up to the last follow-up; and falls with incident hip fracture at t0. Further details are provided in the appendix (Table A in S1 Appendix).

### Physical function, depressive symptoms, and cognitive performance

In ELSA and HRS, physical function was assessed using a self-administered questionnaire on difficulties in ADLs. Participants were asked whether they had any difficulty with walking across a room, dressing, bathing, eating, getting in and out of bed, and getting on and off the toilet. In CHARLS, an additional ADL item on difficulty controlling urination and defecation was used instead of the activity of walking across a room, which was not available in this study. The summed ADL score reflected participants’ physical functioning, with higher scores indicating poorer functioning.

Depressive symptoms were assessed through standardized scales: the 8-item version of the Center for Epidemiologic Studies Depression Scale (CESD-8) in ELSA and HRS [20], and the 10-item version of the CESD scale in CHARLS [21]. Higher scores on the CESD scales indicate more severe depressive symptoms.

Cognitive performance was assessed through the following domains: episodic memory (immediate and delayed word recall), orientation (date naming for year, month, day, and day of the week), executive function (serial 7’s subtraction in CHARLS and HRS), and visuospatial ability (a picture-drawing test only in CHARLS). Notably, from 2018 onward (wave 4), CHARLS revised the administration procedures and word lists of the episodic memory test to improve harmonization with HRS-family studies. Accordingly, we used a weighted equipercentile equating approach to recalibrate the 2018 and 2020 word recall scores based on the percentile distribution in 2015 [22]. The composite cognitive scores reflect participants’ cognitive performance, with higher scores indicating better cognitive performance.

To facilitate the interpretation of trajectories and ensure a consistent score direction across the three functional domains, the ADL and depressive scores were reverse-coded, with lower scores indicating poorer functioning. We further standardized the baseline distributions of the three functional domains into cohort-specific *z*-scores (i.e., mean = 0 and standard deviation [SD] = 1) to enable comparability across the three cohorts, and follow-up scores were standardized using the corresponding baseline distributions to ensure comparability over time within each study [23]. Further details of the assessments of physical function, depressive symptoms, and cognitive performance are provided in the appendix (Table A in S1 appendix).

### Covariates

Covariates were drawn from the baseline wave and included sociodemographic characteristics (age, sex, education level [primary, secondary, tertiary], net household wealth [Q1–Q4], and marital status [married or partnered; widowed; separate or divorced or single]), health-related lifestyle factors (current smoking status, alcohol consumption, and physical activity), eyesight, hearing, and chronic conditions (hypertension, diabetes, chronic lung disease, heart problem, stroke, arthritis, cancer, and memory-related diseases). Given their particular relevance to falls, stroke, arthritis, and memory-related diseases were included as separate covariates [24,25], while other chronic conditions were summarized as the number of comorbidities (0, 1, and ≥2). Missing values for covariates were categorized as “unknown”. Harmonized covariate classifications are shown in the appendix (Table A in S1 Appendix).

### Statistical analysis

Baseline characteristics of the participants were summarized as mean (SD) for continuous variables and number (percentage) for categorical variables, stratified by cohort and fall status. Differences between groups were compared using analysis of variance or the chi-squared tests, as appropriate. To visually inspect changes in physical function, depressive symptoms, and cognitive performance over time, we used local polynomial models to plot raw trajectories and calculated differences in original functional scores every 2 years using false discovery rate-corrected *t* tests, according to fall status, falls with injuries, recurrent falls, and falls with hip fracture.

#### Separate trajectory analysis.

Piecewise linear mixed-effects models were used to capture the separate trajectories of physical function, depressive symptoms, and cognitive performance before and after the wave of first reported falls. Mixed-effects models with piecewise regression modeling use all available data, can effectively handle nonmonotone missing patterns, and allow the slope to differ before and after a predefined time point (t0) [26]. The fixed effects included group (fall status or other fall-related characteristics), periods before and after t0 (pre-time and post-time), and interactions of group with pre-time and post-time. Individuals were included as random effects in the models. Models were adjusted for age, sex, education level, net household wealth, marital status, current smoking status, alcohol consumption, physical activity, eyesight, hearing, stroke, arthritis, memory-related diseases, and the number of other comorbidities. In addition, quadratic terms for pre-time and post-time, as well as their interactions with group, were included in the models if they were statistically significant according to the Wald test. To ensure statistical power, the falls with hip fracture group was not included in model fitting for the period earlier than 7 years before the fall (*n* < 20).

#### Joint trajectory analysis.

We further identified the most common joint trajectories of physical function, depressive symptoms, and cognitive performance before and after falls using group-based multi-trajectory modeling (GBMTM). GBMTM is an approach used to identify latent subgroups of individuals who follow similar trajectories across multiple indicators of interest. The optimal number and shape of the trajectory groups were determined by the Bayesian Information Criterion (BIC, values closer to zero), the average posterior probabilities of assignment (Ave pp > 0.7), and the sample sizes in each group [27]. We then used multinomial logistic models to estimate odds ratios (ORs) and 95% confidence intervals (CIs) for the associations between falls and the identified joint trajectories.

#### Subgroup and sensitivity analyses.

We conducted subgroup analyses to examine whether differences between the falls and no falls groups in functional trajectories varied by age (<75 or ≥75 years), sex, and socioeconomic status (including education level and net household wealth). Multiplicative interaction terms were used to test interactions across subgroups. Several sensitivity analyses were conducted to assess the robustness of the results: (1) restricting the analysis to participants who had at least one measurement both before and after the fall (matching) time point, thereby providing information on both pre-time and post-time; (2) restricting the analysis to participants with at least three repeated measurements to improve the precision of trajectory estimation; (3) conducting spline-based mixed-effects models with natural cubic splines of 2 degrees of freedom, selected based on the BIC, to allow for potential nonlinearity in functional trajectories; (4) redefining physical function using five ADL items common to all cohorts; (5) imputing missing covariates using multiple imputation and generating 20 complete datasets, with results combined according to Rubin’s rules, to reduce potential bias due to missing data (for separate and joint trajectory analyses); and (6) conducting inverse probability weighting for the joint trajectory analysis to maintain sample representativeness comparable to that in the separate trajectory analysis and to mitigate potential selection bias.

The study hypotheses and analytical approach were developed before the main statistical analyses were conducted, and the analyses were performed between February and April 2026. No changes to the analytical approach were made based on data-driven findings. The “traj” command in Stata 17 MP was used for the GBMTM. All other analyses were performed using R software (version 4.5.1). A two-sided *P* value < 0.05 was considered statistically significant.

## Results

### Baseline characteristics

A total of 10,990 participants, including 5,495 matched pairs of participants who experienced falls and those who did not, were included to investigate trajectories of physical function, depressive symptoms, and cognitive performance before and after incident falls over 10 years of follow-up, of whom 3,038 (27.6%) from China, 3,540 (32.2%) from the UK, and 4,412 (40.1%) from the USA (Table B in S1 Appendix). The mean (SD) age at baseline was 71.0 (7.2) years, and 5,010 (45.6%) participants were male. Compared with participants who did not experience falls, those who experienced falls were more likely to be widowed and physically inactive, to have poorer vision and hearing, to have a history of stroke, arthritis, and memory-related diseases, and to have a higher number of other comorbidities at baseline (Table 1). The characteristics of participants by fall-related characteristics are shown in the appendix (Tables C–E in S1 Appendix).

**Table 1:** Baseline characteristics of participants by falls status.

	Total (*n* = 10,990)	No falls (*n* = 5,495)	Falls (*n* = 5,495)	*P*
**Cohort study, *n* (%)**				>0.999
CHARLS	3,038 (27.6)	1,519 (27.6)	1,519 (27.6)	
ELSA	3,540 (32.2)	1,770 (32.2)	1,770 (32.2)	
HRS	4,412 (40.1)	2,206 (40.1)	2,206 (40.1)	
**Age, mean (SD)**	71.0 (7.2)	70.5 (7.0)	71.4 (7.4)	<0.001
**Sex, *n* (%)**				>0.999
Male	5,010 (45.6)	2,505 (45.6)	2,505 (45.6)	
Female	5,980 (54.4)	2,990 (54.4)	2,990 (54.4)	
**Education level, *n* (%)**				0.576
Primary	4,802 (43.7)	2,413 (43.9)	2,389 (43.5)	
Secondary	4,366 (39.7)	2,184 (39.7)	2,182 (39.7)	
Tertiary	1,525 (13.9)	742 (13.5)	783 (14.2)	
Unknown	297 (2.7)	156 (2.8)	141 (2.6)	
**Total household wealth, *n* (%)**				0.865
Q1 (lowest)	2,722 (24.8)	1,344 (24.5)	1,378 (25.1)	
Q2	2,721 (24.8)	1,359 (24.7)	1,362 (24.8)	
Q3	2,721 (24.8)	1,384 (25.2)	1,337 (24.3)	
Q4 (highest)	2,720 (24.7)	1,355 (24.7)	1,365 (24.8)	
Unknown	106 (1.0)	53 (1.0)	53 (1.0)	
**Marital status, *n* (%)**				0.029
Married or partnered	7,683 (69.9)	3,898 (70.9)	3,785 (68.9)	
Widowed	2,217 (20.2)	1,054 (19.2)	1,163 (21.2)	
Separated or divorced or single	1,088 (9.9)	543 (9.9)	545 (9.9)	
Unknown	2 (0.0)	0 (0.0)	2 (0.0)	
**Current smoking status, *n* (%)**				0.184
No	9,352 (85.1)	4,643 (84.5)	4,709 (85.7)	
Yes	1,608 (14.6)	835 (15.2)	773 (14.1)	
Unknown	30 (0.3)	17 (0.3)	13 (0.2)	
**Alcohol consumption, *n* (%)**				0.870
Less than weekly drinking	6,403 (58.3)	3,188 (58.0)	3,215 (58.5)	
Weekly drinking or more	4,176 (38.0)	2,101 (38.2)	2075 (37.8)	
Unknown	411 (3.7)	206 (3.7)	205 (3.7)	
**Physical activity, *n* (%)**				<0.001
Physical inactive	1848 (16.8)	834 (15.2)	1,014 (18.5)	
Physical active	7,360 (67.0)	3,766 (68.5)	3,594 (65.4)	
Unknown	1,782 (16.2)	895 (16.3)	887 (16.1)	
**Eyesight, *n* (%)**				<0.001
Good or better	7,259 (66.1)	3,726 (67.8)	3,533 (64.3)	
Fair or worse	3,715 (33.8)	1,760 (32.0)	1955 (35.6)	
Unknown	16 (0.1)	9 (0.2)	7 (0.1)	
**Hearing, *n* (%)**				<0.001
Good or better	7,394 (67.3)	3,822 (69.6)	3,572 (65.0)	
Fair or worse	3,590 (32.7)	1,671 (30.4)	1919 (34.9)	
Unknown	6 (0.1)	2 (0.0)	4 (0.1)	
**Stroke, *n* (%)**				<0.001
No	10,406 (94.7)	5,252 (95.6)	5,154 (93.8)	
Yes	579 (5.3)	242 (4.4)	337 (6.1)	
Unknown	5 (0.0)	1 (0.0)	4 (0.1)	
**Arthritis, *n* (%)**				<0.001
No	5,660 (51.5)	3,081 (56.1)	2,579 (46.9)	
Yes	5,324 (48.4)	2,410 (43.9)	2,914 (53.0)	
Unknown	6 (0.1)	4 (0.1)	2 (0.0)	
**Memory-related diseases, *n* (%)**				<0.001
No	10,824 (98.5)	5,432 (98.9)	5,392 (98.1)	
Yes	160 (1.5)	60 (1.1)	100 (1.8)	
Unknown	6 (0.1)	3 (0.1)	3 (0.1)	
**Number of other comorbidities, *n* (%)**				<0.001
0	3,719 (33.8)	1961 (35.7)	1,758 (32.0)	
1	4,017 (36.6)	2061 (37.5)	1956 (35.6)	
≥ 2	3,207 (29.2)	1,448 (26.4)	1,759 (32.0)	
Unknown	47 (0.4)	25 (0.5)	22 (0.4)	

*P* values were calculated by analysis of variance or chi-squared test when appropriate.

Abbreviations: CHARLS, China Health and Retirement Longitudinal Study; ELSA, English Longitudinal Study on Ageing; HRS, US Health and Retirement Study.

### Separate trajectory analysis

Figs A–D in S1 Appendix show the raw trajectories of physical function, depressive symptoms, and cognitive performance before and after falls and fall-related characteristics. Among participants with incident falls, physical (difference in mean: −0.14, 95% CI [−0.19, −0.09]) and cognitive functioning (difference in mean: −0.07, 95% CI [−0.12, −0.01]) were poorer than among participants without reported falls, beginning 3–5 years before the fall, whereas depressive symptoms (difference in mean: −0.15, 95% CI [−0.23, −0.08]) was more severe beginning 5–7 years before the fall (Table F in S1 Appendix). These differences persisted up to 5 years after the fall and were more pronounced among those with injurious falls, hip fractures, and recurrent falls (Tables G–I in S1 Appendix).

**Fig 2:**
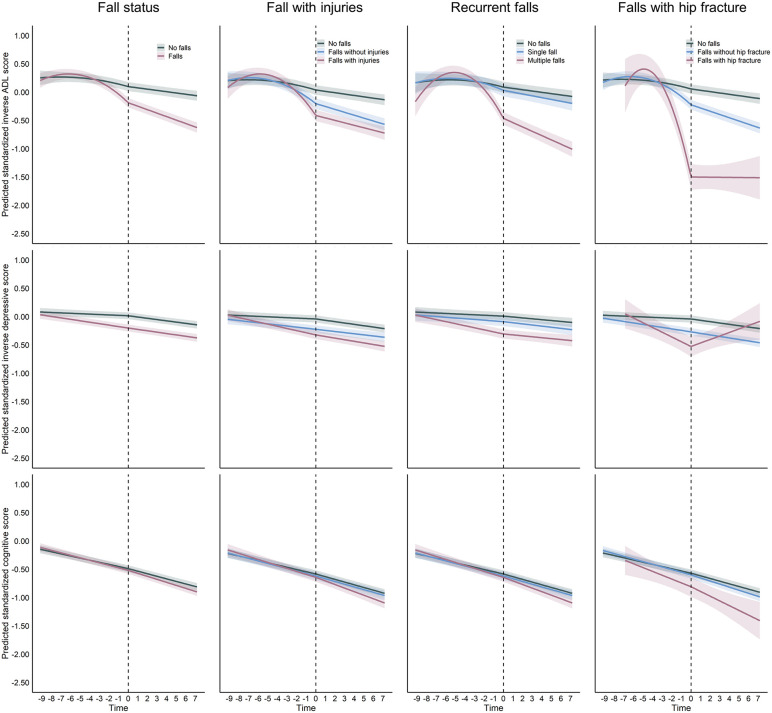
Trajectories of physical function, depressive symptoms, and cognitive performance before and after falls and fall-related characterizations. Models were adjusted age, sex, cohort, education level, total household wealth, marital status, current smoking status, current alcohol consumption, physical activity, stroke, arthritis, memory-related diseases, eyesight, hearing, and number of other comorbidities.

After adjusting for covariates, participants who experienced falls had poorer physical function (β_falls_ = −0.285, 95% CI [−0.331, −0.239]) and more severe depressive symptoms (β_falls_ = −0.217, 95% CI [−0.250, −0.184]) at t0 than matched participants without reported falls (Fig 2, Table 2). Participants who experienced falls also exhibited faster worsening in physical function both before (β_fall * pre-time_ = −0.116, 95% CI [−0.142, −0.089]; β_fall * pre-time^2_ = −0.010, 95% CI [−0.014, −0.006]) and after t0 (β_fall * post-time_ = −0.039, 95% CI [−0.050, −0.028]), whereas faster worsening in depressive symptoms was observed only before t0 (β_fall * pre-time_ = −0.018, 95% CI [−0.026, −0.011]), and faster decline in cognitive performance only after t0 (β_fall * post-time_ = −0.008, 95% CI [−0.016, 0.000], Table 2).

**Table 2:** Estimated changes in physical function, depressive symptoms, and cognitive performance before and after falls and fall-related injuries.

	Physical function β (95% CI)	Depressive symptoms β (95% CI)	Cognitive performance β (95% CI)
**Fall status** ^**a**^			
Falls *(n = 5,495)*	**−0.285 (−0.331, −0.239)**	**−0.217 (−0.250, −0.184)**	−0.029 (−0.062, 0.004)
Falls × pre-time	**−0.116 (−0.142, −0.089)**	**−0.018 (−0.026, −0.011)**	−0.007 (−0.014, 0.001)
Falls × pre-time^2^	**−0.010 (−0.014, −0.006)**	NA	NA
Falls × post-time	**−0.039 (−0.050, −0.028)**	−0.002 (−0.010, 0.006)	**−0.008 (−0.016, 0.000)**
**Injuries from falls** ^**a**^			
Falls without injuries *(n = 3,131)*	**−0.226 (−0.279, −0.174)**	**−0.172 (−0.210, −0.134)**	−0.033 (−0.072, 0.005)
Falls with injuries *(n = 1,795)*	**−0.424 (−0.488, −0.360)**	**−0.266 (−0.312, −0.219)**	**−0.057 (−0.104, −0.009)**
Falls without injuries × pre-time	**−0.077 (−0.106, −0.047)**	**−0.011 (−0.020, −0.002)**	−0.004 (−0.013, 0.004)
Falls with injuries × pre-time	**−0.189 (−0.225, −0.153)**	**−0.029 (−0.040, −0.018)**	**−0.012 (−0.023, −0.002)**
Falls without injuries × pre-time^2^	**−0.006 (−0.010, −0.001)**	NA	NA
Falls with injuries × pre-time^2^	**−0.017 (−0.022, −0.012)**	NA	NA
Falls without injuries × post-time	**−0.026 (−0.038, −0.013)**	0.004 (−0.005, 0.013)	0.000 (−0.010, 0.009)
Falls with injuries × post-time	**−0.018 (−0.033, −0.003)**	−0.004 (−0.015, 0.007)	**−0.014 (−0.026, −0.003)**
**Recurrent falls** ^**b**^			
Single fall *(n = 1,901)*	−0.054 (−0.117, 0.010)	**−0.092 (−0.138, −0.046)**	0.019 (−0.027, 0.064)
Multiple falls *(n = 1,516)*	**−0.520 (−0.589, −0.451)**	**−0.297 (−0.348, −0.247)**	**−0.124 (−0.174, −0.073)**
Single fall × pre-time	−0.027 (−0.063, 0.008)	−0.005 (−0.016, 0.006)	−0.006 (−0.016, 0.004)
Multiple falls × pre-time	**−0.256 (−0.296, −0.216)**	**−0.027 (−0.040, −0.014)**	**−0.020 (−0.033, −0.008)**
Single fall × pre-time^2^	−0.002 (−0.008, 0.003)	NA	NA
Multiple falls × pre-time^2^	**−0.025 (−0.032, −0.019)**	NA	NA
Single fall × post-time	−0.009 (−0.024, 0.007)	−0.004 (−0.016, 0.008)	−0.007 (−0.017, 0.004)
Multiple falls × post-time	**−0.050 (−0.067, −0.033)**	−0.001 (−0.014, 0.012)	−0.005 (−0.017, 0.006)
**Falls with hip fracture** ^**c**^			
Falls without hip fracture *(n = 5,120)*	**−0.263 (−0.309, −0.218)**	**−0.214 (−0.248, −0.181)**	−0.028 (−0.062, 0.006)
Falls with hip fracture *(n = 135)*	**−1.467 (−1.672, −1.261)**	**−0.458 (−0.617, −0.299)**	**−0.226 (−0.386, −0.065)**
Falls without hip fracture × pre-time	**−0.104 (−0.130, −0.078)**	**−0.018 (−0.026, −0.010)**	−0.007 (−0.015, 0.000)
Falls with hip fracture × pre-time	**−0.686 (−0.822, −0.550)**	**−0.072 (−0.113, −0.032)**	−0.027 (−0.067, 0.012)
Falls without hip fracture × pre-time^2^	**−0.009 (−0.013, −0.005)**	NA	NA
Falls with hip fracture × pre-time^2^	**−0.071 (−0.095, −0.047)**	NA	NA
Falls without hip fracture × post-time	**−0.032 (−0.043, −0.021)**	−0.003 (−0.011, 0.005)	−0.007 (−0.015, 0.001)
Falls with hip fracture × post-time	0.021 (−0.035, 0.076)	**0.081 (0.035, 0.126)**	−0.034 (−0.080, 0.011)

Models were adjusted age, sex, cohort, education level, total household wealth, marital status, current smoking status, current alcohol consumption, physical activity, stroke, arthritis, memory-related diseases, eyesight, hearing, and number of other comorbidities.

NA: not applicable for the quadratic time term.

Bolded text indicates statistically significant.

^a^Available for CHARLS, ELSA, and HRS, Ref: No falls (*n* = 5,495).

^b^Available for ELSA, and HRS, Ref: No falls (*n* = 3,976).

^c^Available for CHARLS, ELSA, and HRS, Ref: No falls (*n* = 5,371).

These differences were more pronounced across indicators of fall severity and related characteristics (Fig 2). For example, participants who experienced injurious falls, recurrent falls, or falls resulting in hip fracture had poorer physical function, more severe depressive symptoms, and poorer cognitive performance at t0 (Table 2). Participants with more severe fall-related characteristics also exhibited faster declines in the three functional domains before t0, with a generally graded pattern (physical function: β_falls with injuries * pre-time_ = −0.189, 95% CI [−0.225, −0.153], β_multiple falls * pre-time_ = −0.256, 95% CI [−0.296, −0.216], β_falls with hip fracture * pre-time_ = −0.686, 95% CI [−0.822, −0.550]; depressive symptoms: β_falls with injuries * pre-time_ = −0.029, 95% CI [−0.040, −0.018], β_multiple falls * pre-time_ = −0.027, 95% CI [−0.040, −0.014], β_falls with hip fracture * pre-time_ = −0.072, 95% CI [−0.113, −0.032]; and cognitive performance: β_falls with injuries * pre-time_ = −0.012, 95% CI [−0.023, −0.002], β_multiple falls * pre-time_ = −0.020, 95% CI [−0.033, −0.008], Table 2). In addition, compared with participants who did not experience falls, steeper declines among participants who had more severe fall-related characteristics were observed in specific functional domains after t0 (e.g., β_multiple falls * post-time_ = −0.050, 95% CI [−0.067, −0.033] for physical function, and β_falls with hip injuries * post-time_ = −0.014, 95% CI [−0.026, −0.003] for cognitive performance).

Results were generally similar across cohorts (Tables J–M in S1 Appendix). Subgroup analyses showed that the differences in physical function, depressive symptoms, and cognitive performance at t0 between participants who experienced falls and those who did not were more pronounced among participants with lower socioeconomic status (both lower education level and lower household wealth) (Table N in S1 Appendix). In addition, among participants aged ≥75 years, the differences in the rates of decline in physical function between participants who experienced falls and those who did not, both before and after t0, were greater than among participants aged <75 years (Table N in S1 Appendix). Similar findings were observed when fall severity was further differentiated (Tables O–Q in S1 Appendix). Sensitivity analyses supported the robustness of our findings (Tables R–V and Fig E in S1 Appendix).

### Joint trajectory analysis

**Fig 3:**
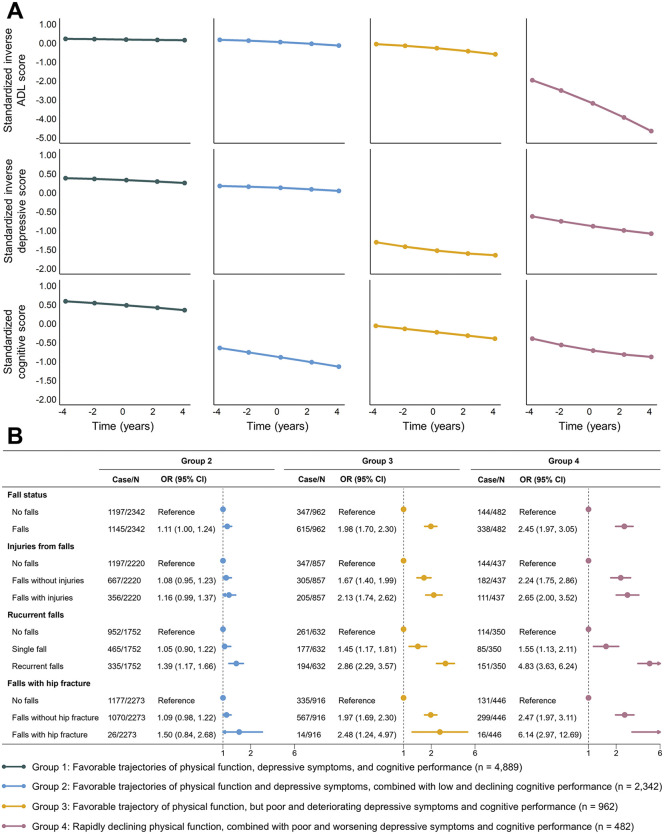
Identification of joint trajectories of physical function, depressive symptoms, and cognitive performance and their associations with fall characteristics. **(A)** Joint trajectories of physical function, depressive symptoms, and cognitive performance before and after falls using Group-based Multi-trajectory Model. **(B)** Associations between falls characterizations joint trajectories of and physical function, depressive symptoms, and cognitive performance. ORs were estimated using multinomial logistic regression models with Group 1 as the reference outcome category, adjusted for age, sex, cohort, education level, total household wealth, marital status, current smoking status, current alcohol consumption, physical activity, and number of comorbidities. Abbreviations: ADL, activities of daily living; OR, odds ratio; CI, confidence interval.

A total of 8,675 participants with at least three repeated measurements of physical function, depressive symptoms, and cognitive performance during follow-up, including 4,366 participants who experienced falls and 4,309 who did not, were included in the joint trajectory analysis (Table W in S1 Appendix). Four distinct trajectory groups were identified (Fig 3A): “Favorable trajectories of physical function, depressive symptoms, and cognitive performance” (*n* = 4,889, 56.4%); “Favorable trajectories of physical function and depressive symptoms, combined with low and declining cognitive performance” (*n* = 2,342, 27.0%); “Favorable trajectory of physical function, but poor and deteriorating depressive symptoms and cognitive performance” (*n* = 962, 11.1%); and “Rapidly declining physical function, combined with poor and worsening depressive symptoms and cognitive performance” (*n* = 482, 5.6%). The fitted parameters of the GBMTM are presented in the appendix (Table X in S1 Appendix), and the baseline characteristics according to the identified trajectory groups are shown in the appendix (Table Y in S1 Appendix).

After adjusting for covariates, participants who experienced falls had progressively higher odds of belonging to increasingly unfavorable joint trajectories of physical function, depressive symptoms, and cognitive performance throughout the follow-up period (OR = 1.11, 95% CI [1.00, 1.24] for the “favorable trajectories of physical function and depressive symptoms, combined with low and declining cognitive performance” group; OR = 1.98, 95% CI [1.70, 2.30] for the “favorable trajectory of physical function, but poor and deteriorating depressive symptoms and cognitive performance” group; and OR = 2.45, 95% CI [1.97, 3.05] for the “rapidly declining physical function, combined with poor and worsening depressive symptoms and cognitive performance” group; Fig 3B). In addition, more severe fall-related characteristics were associated with higher odds of unfavorable joint trajectories across the three functional domains, suggesting a clear graded pattern (e.g., OR _falls with injuries_ = 2.65, 95% CI [2.00, 3.52]; OR _multiple falls_ = 4.83, 95% CI [3.63, 6.24]; and OR _falls with hip fracture_ = 6.14, 95% CI [2.97, 12.69] for the “rapidly declining physical function, combined with poor and worsening depressive symptoms and cognitive performance” group). Similar results were observed across cohorts (Table Z in S1 Appendix) and in subgroups defined by age, sex, and socioeconomic status (Tables AA–DD in S1 Appendix). Sensitivity analyses supported the robustness of the findings (Tables EE–FF in S1 Appendix).

## Discussion

Using longitudinal individual-level data from cohorts in China, the UK, and the USA, this study yielded four main findings. First, participants who experienced falls, particularly with more severe fall-related characteristics, had worse physical function, depressive symptoms, and cognitive performance at the time of the fall than those who did not fall, and these differences were already apparent up to 5 years before the fall. Second, compared with participants who did not experience falls, those who experienced falls exhibited faster decline in physical functioning throughout the follow-up period, whereas faster worsening in depressive symptoms was observed before the first reported fall. Although a more marked decline in cognitive performance was observed only after the fall, faster pre-fall cognitive decline was observed among participants with more severe fall-related characteristics. Third, throughout the follow-up period, participants who experienced falls were more likely to belong to groups with less favorable joint trajectories across the three functional domains. Finally, less favorable multidomain functional trajectories were most commonly observed among participants who experienced more severe falls.

The present findings extend previous evidence on bidirectional associations between falls and ADL disability, depressive symptoms, and cognitive performance, which has largely relied on longitudinal time-to-event analyses reflecting between-person differences. Our findings showed that participants who experienced falls had poorer multidomain functioning 5–7 years before the fall, with these differences persisting for up to 5 years afterward. These findings are consistent with a Swedish cohort study reporting that increased ADL dependence over 6 years of follow-up was associated with higher odds of falls [28]. Similarly, in a study of older US adults aged ≥70 years, participants who experienced falls showed unfavorable trajectories of physical functioning before the fall and were less likely to recover afterwards, particularly those with hip fracture [29]. Our findings on depression symptoms align with results from the US Health and Retirement study, where a 0.5-SD increase in depression symptoms was associated with an ~30% higher risk of falls two years later [30]. In addition, individuals with less favorable trajectories may have a greater burden of risk factors [11], underscoring the importance of initiating fall prevention as early as possible and sustaining it after a fall. Regular assessments are important for identifying individuals at risk and implementing timely preventive interventions, such as strength, balance, and cognitive training [6,13], while continued post-fall management may help reduce the risk of recurrent falls [2,31]. Together, this highlights a long window for prevention and intervention.

Three findings may have implications for future prevention research. First, the most pronounced functional declines were observed among participants who experienced falls with hip fracture. These falls were associated with markedly poorer cognitive performance and a steep decline in physical function, but, unexpectedly, some improvement in depressive symptoms after the fall. Whether survivor bias contributed to this pattern remains uncertain [32]. In addition, similar to our findings, a cross-sectional study from Germany found that individuals with higher educational attainment had better post-fall physical and cognitive functioning, with no difference in mental health [33]. These findings suggest that personal and social resources in older adults may be related to the risk and recovery of severe fall-related outcomes [6].

Second, the associations with cognitive performance were smaller than those for ADL disability and depressive symptoms and should therefore be interpreted cautiously. Our spline-based mixed-effects models suggested that post-fall cognitive decline may be delayed relative to changes in physical function and depressive symptoms, raising a hypothesis that physical and psychological functioning may partly link falls with cognitive decline. A Swedish community-based study of 2,267 community-dwelling older adults showed that physical performance and depressive symptoms mediated ~26% and 8% of this association, respectively [34]. Further studies are needed to clarify the temporal interplay among these functional trajectories before and after falls.

Third, joint trajectory analyses further indicated that participant who experienced falls were more likely to experience long-term decline in at least one functional domain both before and after the fall, with the highest likelihood of simultaneous decline across all three domains observed among those with more severe fall-related characteristics. These findings support the use of multifactorial approaches in fall prevention and post-fall management, particularly in vulnerable older adults [4]. Interventions targeting physical functioning for older adults who experienced falls may also have benefits for depressive symptoms and cognitive health or broader well-being [31,35]. Nevertheless, interventional research is needed to test whether strategies targeting multiple functional domains outperform those focused on physical functioning only.

These findings have important implications for fall prevention and public health. Short assessments that are validated and feasible for clinical practice may help identify older adults at higher risk of falls [2,4,6]. The choice of instrument should depend on the specific functional domain being assessed. Examples include questionnaires for depressive symptoms; gait speed, the Timed Up and Go test, or the Short Physical Performance Battery for physical function; and validated cognitive screening tools [2,4,6]. When decline in these functional domains is identified, individualized support tailored to functional needs may be warranted to help reduce falls and their more severe consequences [2,6]. Regular screening that tracks functional changes may facilitate early detection of decline. For example, our multi-trajectory modeling suggests that even modest deterioration, ~0.25 SD in any of the three domains relative to the baseline distribution, may be associated with higher odds of falls. Once a fall occurs, older adults’ support systems and the healthcare system should provide comprehensive support to promote recovery across these domains and prevent further decline, while older adults themselves should be encouraged to actively engage in post-fall assessment, rehabilitation, and self-management as well [2]. Health professionals should adopt multidimensional screening and intervention strategies, with priority given to vulnerable populations at higher risk of falls and with fewer socioeconomic resources [2]. Policy leaders should coordinate multidisciplinary interventions and promote community-based programs aimed at improving multidimensional functioning for fall prevention [2,4].

Further mechanistic research is warranted. Physical limitations, depression, and cognitive impairment may increase fall risk through impaired gait and balance, while fall-related injuries and concerns about falling may further restrict physical and social activities, potentially creating a vicious cycle [5,6,36–38]. Declines in one functional domain may also affect other domains [5,6], which may explain why participants who experienced falls showed persistently less favorable joint trajectories across all three domains. Shared determinants, such as socioeconomic adversity, may further contribute through preexisting vulnerabilities and unmet healthcare needs.

The strengths of this study include the use of large-scale longitudinal data on older adults from three countries over a 10-year period and a matched-group design that enabled examination of trajectories of physical, psychological, and cognitive functioning before and after falls. In contrast to earlier studies that focused on single outcomes assessed at one time point, we examined multidomain trajectories and considered differed different fall-related characteristics. In addition, by accounting for falls and related characteristics across the full follow-up period during participant inclusion and exclusion, we reduced the likelihood of misclassification.

There are also several limitations to this study. First, physical and psychological functioning were self-reported and may therefore be subject to reporting bias. Second, fall history may be affected by recall bias, particularly when a fall occurred a long time ago and had no severe consequences. In addition, falls before cohort entry could not be fully assessed. Therefore, some participants with earlier falls may have been misclassified into the no-fall group, which may have led to underestimation of the observed associations. Third, the study design did not allow detailed characterization of functional changes with repeated falls across multiple waves. Fourth, severe falls and hip fractures may be fatal in older adults; participants who died shortly after a fall or were lost to follow-up may introduce survivor bias, and this may also have attenuated the observed associations. Although sensitivity analyses required follow-up data both before and after the fall event and at least three waves of follow-up, the impact of competing risk of death could not be fully excluded. Fifth, between-cohort differences (e.g., mean age and the instruments used to assess functioning) should be taken into account. Therefore, cohort-specific interpretations may still be more appropriate in certain contexts. In addition, although z-scores are used to harmonize such heterogeneity, some valuable original information may have been lost. Sixth, despite matching and adjustment for some key covariates, residual confounding may remain. Seventh, data unavailability limited our ability to assess additional fall severity indicators suggested by the World Falls Guidelines, such as frailty and loss of consciousness after a fall. Eighth, the ADL measures used to assess physical functioning are relatively coarse and may primarily reflect underlying frailty. Ninth, data on medication use and surgery were not available. Lastly, because participants were community-dwelling older adults from three countries, the findings may not generalize to hospitalized populations or other settings.

In summary, falls at older ages are often associated with long-term multidomain functional deterioration, particularly in relation to more severe fall events. These findings support multifactorial risk assessment, prevention, and post-fall management strategies that comprehensively consider physical, psychological, and cognitive domains, rather than focusing solely on physical functioning.

### Ethics approval and informed consent

The CHARLS was approved by the Ethical Review Committee at Peking University. The ELSA was approved by the South Central-Berkshire Research Ethics Committee. The HRS was approved by the Institute for Social Research and the Survey Research Center at the University of Michigan.

## Supporting information

S1 Checklist STROBE Checklist. The STROBE checklist is best used in conjunction with this article (freely available on the websites of PLoS Medicine at http://www.plosmedicine.org/, Annals of Internal Medicine at http://www.annals.org/, and Epidemiology at http://www.epidem.com/). Information on the STROBE Initiative is available at www.strobe-statement.org.
(DOCX)

S1 Appendix Tables A–FF and Figures A–E. **Table A.** Harmonized strategies for key variables included in the study. **Table B.** Baseline characteristics of participants by cohort studies. **Table C.** Baseline characteristics of participants by injuries from falls. **Table D.** Baseline characteristics of participants by recurrent falls. **Table E.** Baseline characteristics of participants by falls with hip fracture. **Table F.** Differences in physical function, depressive symptoms, and cognitive performance before and after falls. **Table G.** Differences in physical function, depressive symptoms, and cognitive performance before and after falls with injuries. **Table H.** Differences in physical function, depressive symptoms, and cognitive performance before and after recurrent falls. **Table I.** Differences in physical function, depressive symptoms, and cognitive performance before and after falls with hip fracture. **Table J.** Estimated changes in physical function, depressive symptoms, and cognitive performance before and after falls by cohort. **Table K.** Estimated changes in physical function, depressive symptoms, and cognitive performance before and after injuries from falls by cohort. **Table L.** Estimated changes in physical function, depressive symptoms, and cognitive performance before and after recurrent falls. **Table M.** Estimated changes in physical function, depressive symptoms, and cognitive performance before and after falls with hip fracture. **Table N.** Subgroup analyses of estimated changes in physical function, depressive symptoms, and cognitive performance before and after falls. **Table O.** Subgroup analyses of estimated changes in physical function, depressive symptoms, and cognitive performance before and after injuries from falls. **Table P.** Subgroup analyses of estimated changes in physical function, depressive symptoms, and cognitive performance before and after recurrent falls. **Table Q.** Subgroup analyses of estimated changes in physical function, depressive symptoms, and cognitive performance before and after hip fracture. **Table R.** Sensitivity analysis 1: including participants with at least one measurement both before and after the fall (matching) time point. **Table S.** Sensitivity analysis 2: including participants with ≥ 3 measurements of physical function, depressive symptoms, and cognitive performance. **Table T.** Sensitivity analysis 3: conducting spline-based mixed-effects models with natural cubic splines of 2 degrees of freedom. **Table U.** Sensitivity analysis 4: redefining ADL using five core items common to all cohorts. **Table V.** Sensitivity analysis 5: imputing missing covariates using multiple imputation. **Table W.** Baseline characteristics of participants stratified by whether included in the analysis of joint trajectories. **Table X.** Joint trajectory models’ results of model fitting process. **Table Y.** Characteristics of the study population included in the analysis of joint trajectories of physical function, depressive symptoms, and cognitive performance by identified trajectories. **Table Z.** Associations between falls and joint trajectories of physical function, depressive symptoms, and cognitive performance by cohort. **Table AA.** Subgroup analyses of the associations between falls and joint trajectories of physical function, depressive symptoms, and cognitive performance. **Table BB.** Subgroup analyses of the associations between falls with injuries and joint trajectories of physical function, depressive symptoms, and cognitive performance. **Table CC.** Subgroup analyses of the associations between recurrent falls and joint trajectories of physical function, depressive symptoms, and cognitive performance. **Table DD.** Subgroup analyses of the associations between falls with hip fracture and joint trajectories of physical function, depressive symptoms, and cognitive performance. **Table EE.** Sensitivity analysis 1 for joint trajectory analyses: conducting inverse probability weighting analysis. **Table FF.** Sensitivity analysis 2 for joint trajectory analyses: imputing missing covariates using multiple imputation. **Fig A.** Physical function, depressive symptoms, and cognitive performance trajectories fitted using local polynomial regression by fall status. **Fig B.** Physical function, depressive symptoms, and cognitive performance trajectories fitted using local polynomial regression by injuries from falls. **Fig C.** Physical function, depressive symptoms, and cognitive performance trajectories fitted using local polynomial regression by recurrent falls. **Fig D.** Physical function, depressive symptoms, and cognitive performance trajectories fitted using local polynomial regression by recurrent falls. **Fig E.** Trajectories of physical function, depressive symptoms, and cognitive performance before and after falls and fall-related characterizations using spline-based mixed-effects models.
(DOCX)

## Abbreviations

ADL: activities of daily living
BIC: Bayesian Information Criterion
CHARLS: China Health and Retirement Longitudinal Study
CIs: confidence intervals
ELSA: English Longitudinal Study of Ageing
GBMTM: group-based multi-trajectory modeling
HRS: Health and Retirement Study
ORs: odds ratios
SD: standard deviation.
