# Supplementary material for: Trajectories of physical function, depressive symptoms, and cognitive performance before and after falls in older adults: A matched multicohort study
Source: PLoS Med. 2026 Aug 3;23(8):e1005083. doi: 10.1371/journal.pmed.1005083 (PMC13446737; doi:10.1371/journal.pmed.1005083)
Supplement: Supplementary file 2 [file pmed.1005083.s002.docx]

**S1 appendix**

**Table A.** Harmonized strategies for key variables included in the study

**Table B.** Baseline characteristics of participants by cohort studies

**Table C.** Baseline characteristics of participants by injuries from falls

**Table D.** Baseline characteristics of participants by recurrent falls

**Table E.** Baseline characteristics of participants by falls with hip fracture

**Table F.** Differences in physical function, depressive symptoms, and cognitive performance before and after falls

**Table G.** Differences in physical function, depressive symptoms, and cognitive performance before and after falls with injuries

**Table H.** Differences in physical function, depressive symptoms, and cognitive performance before and after recurrent falls

**Table I.** Differences in physical function, depressive symptoms, and cognitive performance before and after falls with hip fracture

**Table J.** Estimated changes in physical function, depressive symptoms, and cognitive performance before and after falls by cohort

**Table K.** Estimated changes in physical function, depressive symptoms, and cognitive performance before and after injuries from falls by cohort

**Table L.** Estimated changes in physical function, depressive symptoms, and cognitive performance before and after recurrent falls

**Table M.** Estimated changes in physical function, depressive symptoms, and cognitive performance before and after falls with hip fracture

**Table N.** Subgroup analyses of estimated changes in physical function, depressive symptoms, and cognitive performance before and after falls

**Table O.** Subgroup analyses of estimated changes in physical function, depressive symptoms, and cognitive performance before and after injuries from falls

**Table P.** Subgroup analyses of estimated changes in physical function, depressive symptoms, and cognitive performance before and after recurrent falls

**Table Q.** Subgroup analyses of estimated changes in physical function, depressive symptoms, and cognitive performance before and after hip fracture

**Table R.** Sensitivity analysis 1: Including participants with at least one measurement both before and after the fall (matching) time point

**Table S.** Sensitivity analysis 2: Including participants with ≥ 3 measurements of physical function, depressive symptoms, and cognitive performance

**Table T.** Sensitivity analysis 3: Conducting spline-based mixed-effects models with natural cubic splines of 2 degrees of freedom

**Table U.** Sensitivity analysis 4: Redefining ADL using five core items common to all cohorts

**Table V.** Sensitivity analysis 5: Imputing missing covariates using multiple imputation

**Table W.** Baseline characteristics of participants stratified by whether included in the analysis of joint trajectories

**Table X.** Joint trajectory models’ results of model fitting process

**Table Y.** Characteristics of the study population included in the analysis of joint trajectories of physical function, depressive symptoms, and cognitive performance by identified trajectories

**Table Z.** Associations between falls and joint trajectories of physical function, depressive symptoms, and cognitive performance by cohort

**Table AA.** Subgroup analyses of the associations between falls and joint trajectories of physical function, depressive symptoms, and cognitive performance

**Table BB.** Subgroup analyses of the associations between falls with injuries and joint trajectories of physical function, depressive symptoms, and cognitive performance

**Table CC.** Subgroup analyses of the associations between recurrent falls and joint trajectories of physical function, depressive symptoms, and cognitive performance

**Table DD.** Subgroup analyses of the associations between falls with hip fracture and joint trajectories of physical function, depressive symptoms, and cognitive performance

**Table EE.** Sensitivity analysis 1 for joint trajectory analyses: Conducting inverse probability weighting analysis

**Table FF.** Sensitivity analysis 2 for joint trajectory analyses: Imputing missing covariates using multiple imputation

**Fig A.** Physical function, depressive symptoms, and cognitive performance trajectories fitted using local polynomial regression by fall status.

**Fig B.** Physical function, depressive symptoms, and cognitive performance trajectories fitted using local polynomial regression by injuries from falls.

**Fig C.** Physical function, depressive symptoms, and cognitive performance trajectories fitted using local polynomial regression by recurrent falls.

**Fig D.** Physical function, depressive symptoms, and cognitive performance trajectories fitted using local polynomial regression by recurrent falls.

**Fig E.** Trajectories of physical function, depressive symptoms, and cognitive performance before and after falls and fall-related characterizations using spline-based mixed-effects models.

**Table A.** Harmonized strategies for key variables included in the study

| **Variables** | **Harmonized values** | **CHARLS** | **ELSA** | **HRS** |
| --- | --- | --- | --- | --- |
| **Fall status** | Yes | Self-reported falls | | |
|  | No | No falls during the follow-up | | |
| **Falls with injuries** | Falls with injuries | Falls requiring medical attention at the first fall report | | |
|  | Falls without injuries | Falls not requiring medical attention at the first fall report and no subsequent fall requiring medical attention during follow-up | | |
|  | No falls | No falls | | |
| **Recurrent falls** | Multiple falls | NA | Multiple falls at the first fall report | |
|  | Single fall |  | A single fall at the first fall report and no multiple falls during subsequent follow-up | |
|  | No falls |  | No falls | |
| **Hip fracture** | Falls with hip fracture | Falls with incident hip fracture at the first fall report | | |
|  | Falls without hip fracture | Falls at the first fall report with no hip fracture history up to the last follow-up | | |
|  | No falls | No falls | | |
| **Physical function** | Standardized inverse z-score | The total score of difficulties in performing ADL, including  “Have any difficulty with dressing because of health and memory problem”  “Have any difficulty with bathing or showering”  “Have any difficulty with eating because of health and memory problem”  “Have any difficulty with getting into or out of bed”  “Have any difficulty with using the toilet, getting up and down”  “Have any difficulty with controlling urination and defecation” | The total score of difficulties in performing ADL, including walking across a room, dressing, bathing or showering, eating, getting in and out of bed, and using toilet.  “Have any difficulty with walking across a room”  “Have any difficulty with dressing because of health and memory problem”  “Have any difficulty with bathing or showering”  “Have any difficulty with eating because of health and memory problem”  “Have any difficulty with getting into or out of bed”  “Have any difficulty with using the toilet, getting up and down” | |
| **Depressive symptoms** | Standardized inverse z-score | The total score of CESD-10 | The total score of CESD-8 | |
| **Cognitive performance** | Standardized z-score | The summed score for the following domains: immediate word recall, delayed word recall, serial 7’ subtraction test, orientation, and picture drawing task. | The summed score for the following domains: immediate word recall, delayed word recall, and orientation. | The summed score for the following domains: immediate word recall, delayed word recall, and serial 7’ subtraction test, orientation. |
| **Education level** | Primary | Less than upper secondary education | | |
|  | Secondary | Upper secondary & vocational training | | |
|  | Tertiary | Tertiary education | | |
| **Total household wealth** | Q1 (lowest) | The sum of all wealth components (including residence, vehicles, saving accounts, etc.) minus other debts at the couple level (the respondent and spouse, if any) in local currencies. | | |
|  | Q2 |  |  |  |
|  | Q3 |  |  |  |
|  | Q4 (highest) |  |  |  |
| **Marital status** | Married or partnered | Married or partnered | | |
|  | Widowed | Widowed | | |
|  | Separated or divorced or single | Separated or divorced or single | | |
| **Smoking status** | Yes | Smokes at the present time | | |
|  | No | Does not smoke at the present time | | |
| **Alcohol consumption** | Weekly drinking or more | The frequency of drinking behavior during the last year: Once a week/2 to 3 days a week/4 to 6 days a week/Daily/ Twice a day / More than twice a day | The frequency of drinking:1-7 days/week | |
|  | Less than weekly drinking | None or doesn’t drink / Once a month / 2 to 3 days a month | 0 day/week | |
| **Physical activity** | Yes | The number of days of vigorous/moderate physical activity for at least 10 minutes every week: 1-7 days | Frequency of taking part in vigorous/moderate physical activity: everyday / more than once a week / once a week / one to three times a month | |
|  | No | None | Hardly ever or never taking part in vigorous/moderate physical activity | |
| **Stroke** | Yes | Self-reported stroke ever diagnosed by a doctor | | |
|  | No | Otherwise | | |
| **Arthritis** | Yes | Self-reported stroke ever diagnosed by a doctor | | |
|  | No | Otherwise | | |
| **Memory-related diseases** | Yes | Self-reported Alzheimer, dementia, or Parkinson’s disease ever diagnosed by a doctor | | Self-reported Alzheimer or dementia ever diagnosed by a doctor |
|  | No | Otherwise | | Otherwise |
| **Eyesight** | Good or better | Self-rated eyesight: excellent, very good, good | | |
|  | Fair or worse | Self-rated eyesight: fair, poor, blind | | |
| **Hearing** | Good or better | Self-rated hearing: excellent, very good, good | | |
|  | Fair or worse | Self-rated hearing: fair, poor | | |
| **Number of other comorbidities** | 0 | Free of hypertension, diabetes, heart problem, chronic lung disease, and cancer | | |
|  | 1 | Having one of hypertension, diabetes, heart problem, chronic lung disease, and cancer | | |
|  | 2 | Having at least two of hypertension, diabetes, heart problem, chronic lung disease, and cancer | | |

Abbreviations: CHARLS, China Health and Retirement Longitudinal Study; ELSA, English longitudinal study of ageing; HRS, Health and Retirement Study; ADL, Activities of Daily Living; CESD, Center for Epidemiologic Studies Depression Scale; NA, not applicable.

**Table B.** Baseline characteristics of participants by cohort studies

|  | **Total (n = 10,990)** | **CHARLS (n = 3,038)** | **ELSA (n = 3,540)** | **HRS (n = 4,412)** |
| --- | --- | --- | --- | --- |
| **Age, mean (SD)** | 71.0 (7.2) | 66.8 (5.7) | 69.7 (7.1) | 74.9 (6.2) |
| **Sex, n (%)** |  |  |  |  |
| Male | 5010 (45.6) | 1428 (47.0) | 1588 (44.9) | 1994 (45.2) |
| Female | 5980 (54.4) | 1610 (53.0) | 1952 (55.1) | 2418 (54.8) |
| **Education level, n (%)** |  |  |  |  |
| Primary | 4802 (43.7) | 2799 (92.1) | 1089 (30.8) | 914 (20.7) |
| Secondary | 4366 (39.7) | 176 (5.8) | 1619 (45.7) | 2571 (58.3) |
| Tertiary | 1525 (13.9) | 63 (2.1) | 536 (15.1) | 926 (21.0) |
| Unknown | 297 (2.7) | 0 (0.0) | 296 (8.4) | 1 (0.0) |
| **Total household wealth, n (%)** |  |  |  |  |
| Q1 (lowest) | 2722 (24.8) | 746 (24.6) | 873 (24.7) | 1103 (25.0) |
| Q2 | 2721 (24.8) | 745 (24.5) | 873 (24.7) | 1103 (25.0) |
| Q3 | 2721 (24.8) | 745 (24.5) | 873 (24.7) | 1103 (25.0) |
| Q4 (highest) | 2720 (24.7) | 745 (24.5) | 872 (24.6) | 1103 (25.0) |
| Unknown | 106 (1.0) | 57 (1.9) | 49 (1.4) | 0 (0.0) |
| **Marital status, n (%)** |  |  |  |  |
| Married or partnered | 7683 (69.9) | 2453 (80.7) | 2473 (69.9) | 2757 (62.5) |
| Widowed | 2217 (20.2) | 532 (17.5) | 598 (16.9) | 1087 (24.6) |
| Separated or divorced or single | 1088 (9.9) | 53 (1.7) | 469 (13.2) | 566 (12.8) |
| Unknown | 2 (0.0) | 0 (0.0) | 0 (0.0) | 2 (0.0) |
| **Current smoking status, n (%)** |  |  |  |  |
| No | 9352 (85.1) | 2155 (70.9) | 3199 (90.4) | 3998 (90.6) |
| Yes | 1608 (14.6) | 883 (29.1) | 341 (9.6) | 384 (8.7) |
| Unknown | 30 (0.3) | 0 (0.0) | 0 (0.0) | 30 (0.7) |
| **Alcohol consumption, n (%)** |  |  |  |  |
| Less than weekly drinking | 6403 (58.3) | 2451 (80.7) | 1110 (31.4) | 2842 (64.4) |
| Weekly drinking or more | 4176 (38.0) | 441 (14.5) | 2180 (61.6) | 1555 (35.2) |
| Unknown | 411 (3.7) | 146 (4.8) | 250 (7.1) | 15 (0.3) |
| **Physical activity, n (%)** |  |  |  |  |
| Physical inactive | 1848 (16.8) | 521 (17.1) | 503 (14.2) | 824 (18.7) |
| Physical active | 7360 (67.0) | 739 (24.3) | 3037 (85.8) | 3584 (81.2) |
| Unknown | 1782 (16.2) | 1778 (58.5) | 0 (0.0) | 4 (0.1) |
| **Eyesight, n (%)** |  |  |  |  |
| Good or better | 7259 (66.1) | 636 (20.9) | 3139 (88.7) | 3484 (79.0) |
| Fair or worse | 3715 (33.8) | 2400 (79.0) | 401 (11.3) | 914 (20.7) |
| Unknown | 16 (0.1) | 2 (0.1) | 0 (0.0) | 14 (0.3) |
| **Hearing, n (%)** |  |  |  |  |
| Good or better | 7394 (67.3) | 1217 (40.1) | 2799 (79.1) | 3378 (76.6) |
| Fair or worse | 3590 (32.7) | 1820 (59.9) | 740 (20.9) | 1030 (23.3) |
| Unknown | 6 (0.1) | 1 (0.0) | 1 (0.0) | 4 (0.1) |
| **Stroke, n (%)** |  |  |  |  |
| No | 10406 (94.7) | 2931 (96.5) | 3415 (96.5) | 4060 (92.0) |
| Yes | 579 (5.3) | 102 (3.4) | 125 (3.5) | 352 (8.0) |
| Unknown | 5 (0.0) | 5 (0.2) | 0 (0.0) | 0 (0.0) |
| **Arthritis, n (%)** |  |  |  |  |
| No | 5660 (51.5) | 1888 (62.1) | 2180 (61.6) | 1592 (36.1) |
| Yes | 5324 (48.4) | 1144 (37.7) | 1360 (38.4) | 2820 (63.9) |
| Unknown | 6 (0.1) | 6 (0.2) | 0 (0.0) | 0 (0.0) |
| **Memory-related diseases, n (%)** |  |  |  |  |
| No | 10824 (98.5) | 2973 (97.9) | 3507 (99.1) | 4344 (98.5) |
| Yes | 160 (1.5) | 59 (1.9) | 33 (0.9) | 68 (1.5) |
| Unknown | 6 (0.1) | 6 (0.2) | 0 (0.0) | 0 (0.0) |
| **Number of other comorbidities, n (%)** |  |  |  |  |
| 0 | 3719 (33.8) | 1512 (49.8) | 1387 (39.2) | 820 (18.6) |
| 1 | 4017 (36.6) | 1018 (33.5) | 1353 (38.2) | 1646 (37.3) |
| ≥ 2 | 3207 (29.2) | 461 (15.2) | 800 (22.6) | 1946 (44.1) |
| Unknown | 47 (0.4) | 47 (1.5) | 0 (0.0) | 0 (0.0) |
| **Injuries from falls, n (%)** |  |  |  |  |
| No falls | 5495 (52.7) | 1519 (52.5) | 1770 (52.6) | 2206 (53.0) |
| Falls without injuries | 3131 (30.0) | 741 (25.6) | 1094 (32.5) | 1296 (31.1) |
| Falls with injuries | 1795 (17.2) | 633 (21.9) | 501 (14.9) | 661 (15.9) |
| **Recurrent falls, n (%)** |  |  |  |  |
| No falls | 3976 (53.8) | NA | 1770 (53.0) | 2206 (54.4) |
| Single fall | 1901 (25.7) | NA | 1044 (31.3) | 857 (21.1) |
| Multiple falls | 1516 (20.5) | NA | 523 (15.7) | 993 (24.5) |
| **Falls with hip fracture, n (%)** |  |  |  |  |
| No falls | 5371 (50.5) | 1461 (50.5) | 1739 (50.4) | 2171 (50.7) |
| Falls without hip fracture | 5120 (48.2) | 1379 (47.7) | 1690 (49.0) | 2051 (47.9) |
| Falls with hip fracture | 135 (1.3) | 54 (1.9) | 22 (0.6) | 59 (1.4) |
| **Number of falls among participants with multiple falls, mean** | NA | NA | 2.83 | 3.40 |

Abbreviations: CHARLS, China Health and Retirement Longitudinal Study; ELSA, English Longitudinal Study on Ageing; HRS, US Health and Retirement Study; NA, not applicable.

**Table C.** Baseline characteristics of participants by injuries from falls

|  | **Total (n = 10,421)** | **No falls (n = 5,495)** | **Falls without injuries (n = 3,131)** | **Falls with injuries (n = 1,795)** | ***P*** |
| --- | --- | --- | --- | --- | --- |
| **Cohort study, n (%)** |  |  |  |  | <0.001 |
| CHARLS | 2893 (27.8) | 1519 (27.6) | 741 (23.7) | 633 (35.3) |  |
| ELSA | 3365 (32.3) | 1770 (32.2) | 1094 (34.9) | 501 (27.9) |  |
| HRS | 4163 (39.9) | 2206 (40.1) | 1296 (41.4) | 661 (36.8) |  |
| **Age, mean (SD)** | 70.9 (7.2) | 70.5 (7.0) | 71.1 (7.1) | 71.7 (7.8) | <0.001 |
| **Sex, n (%)** |  |  |  |  | <0.001 |
| Male | 4781 (45.9) | 2505 (45.6) | 1581 (50.5) | 695 (38.7) |  |
| Female | 5640 (54.1) | 2990 (54.4) | 1550 (49.5) | 1100 (61.3) |  |
| **Education level, n (%)** |  |  |  |  | <0.001 |
| Primary | 4557 (43.7) | 2413 (43.9) | 1252 (40.0) | 892 (49.7) |  |
| Secondary | 4138 (39.7) | 2184 (39.7) | 1312 (41.9) | 642 (35.8) |  |
| Tertiary | 1438 (13.8) | 742 (13.5) | 476 (15.2) | 220 (12.3) |  |
| Unknown | 288 (2.8) | 156 (2.8) | 91 (2.9) | 41 (2.3) |  |
| **Total household wealth, n (%)** |  |  |  |  | 0.689 |
| Q1 (lowest) | 2574 (24.7) | 1344 (24.5) | 757 (24.2) | 473 (26.4) |  |
| Q2 | 2586 (24.8) | 1359 (24.7) | 773 (24.7) | 454 (25.3) |  |
| Q3 | 2591 (24.9) | 1384 (25.2) | 774 (24.7) | 433 (24.1) |  |
| Q4 (highest) | 2569 (24.7) | 1355 (24.7) | 797 (25.5) | 417 (23.2) |  |
| Unknown | 101 (1.0) | 53 (1.0) | 30 (1.0) | 18 (1.0) |  |
| **Marital status, n (%)** |  |  |  |  | <0.001 |
| Married or partnered | 7296 (70.0) | 3898 (70.9) | 2218 (70.8) | 1180 (65.7) |  |
| Widowed | 2075 (19.9) | 1054 (19.2) | 579 (18.5) | 442 (24.6) |  |
| Separated or divorced or single | 1048 (10.1) | 543 (9.9) | 333 (10.6) | 172 (9.6) |  |
| Unknown | 2 (0.0) | 0 (0.0) | 1 (0.0) | 1 (0.1) |  |
| **Current smoking status, n (%)** |  |  |  |  | 0.104 |
| No | 8861 (85.0) | 4643 (84.5) | 2700 (86.2) | 1518 (84.6) |  |
| Yes | 1531 (14.7) | 835 (15.2) | 421 (13.4) | 275 (15.3) |  |
| Unknown | 29 (0.3) | 17 (0.3) | 10 (0.3) | 2 (0.1) |  |
| **Alcohol consumption, n (%)** |  |  |  |  | 0.041 |
| Less than weekly drinking | 6055 (58.1) | 3188 (58.0) | 1770 (56.5) | 1097 (61.1) |  |
| Weekly drinking or more | 3980 (38.2) | 2101 (38.2) | 1241 (39.6) | 638 (35.5) |  |
| Unknown | 386 (3.7) | 206 (3.7) | 120 (3.8) | 60 (3.3) |  |
| **Physical activity, n (%)** |  |  |  |  | <0.001 |
| Physical inactive | 1737 (16.7) | 834 (15.2) | 568 (18.1) | 335 (18.7) |  |
| Physical active | 6991 (67.1) | 3766 (68.5) | 2143 (68.4) | 1082 (60.3) |  |
| Unknown | 1693 (16.2) | 895 (16.3) | 420 (13.4) | 378 (21.1) |  |
| **Eyesight, n (%)** |  |  |  |  | <0.001 |
| Good or better | 6887 (66.1) | 3726 (67.8) | 2092 (66.8) | 1069 (59.6) |  |
| Fair or worse | 3519 (33.8) | 1760 (32.0) | 1035 (33.1) | 724 (40.3) |  |
| Unknown | 15 (0.1) | 9 (0.2) | 4 (0.1) | 2 (0.1) |  |
| **Hearing, n (%)** |  |  |  |  | <0.001 |
| Good or better | 7029 (67.5) | 3822 (69.6) | 2082 (66.5) | 1125 (62.7) |  |
| Fair or worse | 3387 (32.5) | 1671 (30.4) | 1046 (33.4) | 670 (37.3) |  |
| Unknown | 5 (0.0) | 2 (0.0) | 3 (0.1) | 0 (0.0) |  |
| **Stroke, n (%)** |  |  |  |  | <0.001 |
| No | 9887 (94.9) | 5252 (95.6) | 2940 (93.9) | 1695 (94.4) |  |
| Yes | 529 (5.1) | 242 (4.4) | 191 (6.1) | 96 (5.3) |  |
| Unknown | 5 (0.0) | 1 (0.0) | 0 (0.0) | 4 (0.2) |  |
| **Arthritis, n (%)** |  |  |  |  | <0.001 |
| No | 5426 (52.1) | 3081 (56.1) | 1502 (48.0) | 843 (47.0) |  |
| Yes | 4989 (47.9) | 2410 (43.9) | 1628 (52.0) | 951 (53.0) |  |
| Unknown | 6 (0.1) | 4 (0.1) | 1 (0.0) | 1 (0.1) |  |
| **Memory-related diseases, n (%)** |  |  |  |  | 0.068 |
| No | 10273 (98.6) | 5432 (98.9) | 3082 (98.4) | 1759 (98.0) |  |
| Yes | 142 (1.4) | 60 (1.1) | 48 (1.5) | 34 (1.9) |  |
| Unknown | 6 (0.1) | 3 (0.1) | 1 (0.0) | 2 (0.1) |  |
| **Number of other comorbidities, n (%)** |  |  |  |  | <0.001 |
| 0 | 3545 (34.0) | 1961 (35.7) | 966 (30.9) | 618 (34.4) |  |
| 1 | 3836 (36.8) | 2061 (37.5) | 1127 (36.0) | 648 (36.1) |  |
| ≥ 2 | 2996 (28.7) | 1448 (26.4) | 1025 (32.7) | 523 (29.1) |  |
| Unknown | 44 (0.4) | 25 (0.5) | 13 (0.4) | 6 (0.3) |  |

Abbreviations: CHARLS, China Health and Retirement Longitudinal Study; ELSA, English Longitudinal Study on Ageing; HRS, US Health and Retirement Study.

**Table D.** Baseline characteristics of participants by recurrent falls

|  | **Total (n = 7,393)** | **No falls (n = 3,976)** | **Single fall (n = 1,901)** | **Multiple falls (n = 1,516)** | ***P*** |
| --- | --- | --- | --- | --- | --- |
| **Cohort study, n (%)** |  |  |  |  | <0.001 |
| ELSA | 3337 (45.1) | 1770 (44.5) | 1044 (54.9) | 523 (34.5) |  |
| HRS | 4056 (54.9) | 2206 (55.5) | 857 (45.1) | 993 (65.5) |  |
| **Age, mean (SD)** | 72.5 (7.1) | 72.0 (6.9) | 72.2 (7.2) | 74.0 (7.2) | <0.001 |
| **Sex, n (%)** |  |  |  |  | 0.011 |
| Male | 3336 (45.1) | 1791 (45.0) | 816 (42.9) | 729 (48.1) |  |
| Female | 4057 (54.9) | 2185 (55.0) | 1085 (57.1) | 787 (51.9) |  |
| **Education level, n (%)** |  |  |  |  | 0.003 |
| Primary | 1860 (25.2) | 1015 (25.5) | 460 (24.2) | 385 (25.4) |  |
| Secondary | 3922 (53.1) | 2098 (52.8) | 994 (52.3) | 830 (54.7) |  |
| Tertiary | 1334 (18.0) | 707 (17.8) | 357 (18.8) | 270 (17.8) |  |
| Unknown | 277 (3.7) | 156 (3.9) | 90 (4.7) | 31 (2.0) |  |
| **Total household wealth, n (%)** |  |  |  |  | 0.108 |
| Q1 (lowest) | 1810 (24.5) | 967 (24.3) | 462 (24.3) | 381 (25.1) |  |
| Q2 | 1845 (25.0) | 1003 (25.2) | 456 (24.0) | 386 (25.5) |  |
| Q3 | 1848 (25.0) | 1024 (25.8) | 462 (24.3) | 362 (23.9) |  |
| Q4 (highest) | 1843 (24.9) | 961 (24.2) | 501 (26.4) | 381 (25.1) |  |
| Unknown | 47 (0.6) | 21 (0.5) | 20 (1.1) | 6 (0.4) |  |
| **Marital status, n (%)** |  |  |  |  | 0.015 |
| Married or partnered | 4877 (66.0) | 2664 (67.0) | 1219 (64.1) | 994 (65.6) |  |
| Widowed | 1559 (21.1) | 795 (20.0) | 418 (22.0) | 346 (22.8) |  |
| Separated or divorced or single | 955 (12.9) | 517 (13.0) | 262 (13.8) | 176 (11.6) |  |
| Unknown | 2 (0.0) | 0 (0.0) | 2 (0.1) | 0 (0.0) |  |
| **Current smoking status, n (%)** |  |  |  |  | 0.361 |
| No | 6695 (90.6) | 3576 (89.9) | 1737 (91.4) | 1382 (91.2) |  |
| Yes | 671 (9.1) | 383 (9.6) | 158 (8.3) | 130 (8.6) |  |
| Unknown | 27 (0.4) | 17 (0.4) | 6 (0.3) | 4 (0.3) |  |
| **Alcohol consumption, n (%)** |  |  |  |  | <0.001 |
| Less than weekly drinking | 3650 (49.4) | 1952 (49.1) | 880 (46.3) | 818 (54.0) |  |
| Weekly drinking or more | 3501 (47.4) | 1896 (47.7) | 956 (50.3) | 649 (42.8) |  |
| Unknown | 242 (3.3) | 128 (3.2) | 65 (3.4) | 49 (3.2) |  |
| **Physical activity, n (%)** |  |  |  |  | <0.001 |
| Physical inactive | 1221 (16.5) | 583 (14.7) | 294 (15.5) | 344 (22.7) |  |
| Physical active | 6168 (83.4) | 3390 (85.3) | 1606 (84.5) | 1172 (77.3) |  |
| Unknown | 4 (0.1) | 3 (0.1) | 1 (0.1) | 0 (0.0) |  |
| **Eyesight, n (%)** |  |  |  |  | <0.001 |
| Good or better | 6170 (83.5) | 3376 (84.9) | 1598 (84.1) | 1196 (78.9) |  |
| Fair or worse | 1209 (16.4) | 592 (14.9) | 301 (15.8) | 316 (20.8) |  |
| Unknown | 14 (0.2) | 8 (0.2) | 2 (0.1) | 4 (0.3) |  |
| **Hearing, n (%)** |  |  |  |  |  |
| Good or better | 5766 (78.0) | 3176 (79.9) | 1477 (77.7) | 1113 (73.4) |  |
| Fair or worse | 1622 (21.9) | 798 (20.1) | 423 (22.3) | 401 (26.5) |  |
| Unknown | 5 (0.1) | 2 (0.1) | 1 (0.1) | 2 (0.1) |  |
| **Stroke, n (%)** |  |  |  |  | <0.001 |
| No | 6957 (94.1) | 3768 (94.8) | 1806 (95.0) | 1383 (91.2) |  |
| Yes | 436 (5.9) | 208 (5.2) | 95 (5.0) | 133 (8.8) |  |
| **Arthritis, n (%)** |  |  |  |  |  |
| No | 3560 (48.2) | 2049 (51.5) | 958 (50.4) | 553 (36.5) | <0.001 |
| Yes | 3833 (51.8) | 1927 (48.5) | 943 (49.6) | 963 (63.5) |  |
| **Memory-related diseases, n (%)** |  |  |  |  | <0.001 |
| No | 7302 (98.8) | 3939 (99.1) | 1881 (98.9) | 1482 (97.8) |  |
| Yes | 91 (1.2) | 37 (0.9) | 20 (1.1) | 34 (2.2) |  |
| **Number of other comorbidities, n (%)** |  |  |  |  | <0.001 |
| 0 | 2095 (28.3) | 1172 (29.5) | 599 (31.5) | 324 (21.4) |  |
| 1 | 2805 (37.9) | 1555 (39.1) | 730 (38.4) | 520 (34.3) |  |
| ≥ 2 | 2493 (33.7) | 1249 (31.4) | 572 (30.1) | 672 (44.3) |  |

Abbreviations: CHARLS, China Health and Retirement Longitudinal Study; ELSA, English Longitudinal Study on Ageing; HRS, US Health and Retirement Study.

**Table E.** Baseline characteristics of participants by falls with hip fracture

|  | **Total (n = 10,626)** | **No falls (n = 5,371)** | **Falls without hip fracture (n = 5,120)** | **Falls with hip fracture (n = 135)** | ***P*** |
| --- | --- | --- | --- | --- | --- |
| **Cohort study, n (%)** |  |  |  |  | <0.001 |
| CHARLS | 2894 (27.2) | 1461 (27.2) | 1379 (26.9) | 54 (40.0) |  |
| ELSA | 3451 (32.5) | 1739 (32.4) | 1690 (33.0) | 22 (16.3) |  |
| HRS | 4281 (40.3) | 2171 (40.4) | 2051 (40.1) | 59 (43.7) |  |
| **Age, mean (SD)** | 70.9 (7.1) | 70.5 (7.0) | 71.2 (7.3) | 74.6 (7.8) | <0.001 |
| **Sex, n (%)** |  |  |  |  | 0.070 |
| Male | 4873 (45.9) | 2456 (45.7) | 2368 (46.2) | 49 (36.3) |  |
| Female | 5753 (54.1) | 2915 (54.3) | 2752 (53.8) | 86 (63.7) |  |
| **Education level, n (%)** |  |  |  |  | 0.020 |
| Primary | 4595 (43.2) | 2333 (43.4) | 2184 (42.7) | 78 (57.8) |  |
| Secondary | 4250 (40.0) | 2153 (40.1) | 2056 (40.2) | 41 (30.4) |  |
| Tertiary | 1493 (14.1) | 731 (13.6) | 748 (14.6) | 14 (10.4) |  |
| Unknown | 288 (2.7) | 154 (2.9) | 132 (2.6) | 2 (1.5) |  |
| **Total household wealth, n (%)** |  |  |  |  | <0.001 |
| Q1 (lowest) | 2611 (24.6) | 1304 (24.3) | 1269 (24.8) | 38 (28.1) |  |
| Q2 | 2638 (24.8) | 1337 (24.9) | 1267 (24.7) | 34 (25.2) |  |
| Q3 | 2631 (24.8) | 1350 (25.1) | 1247 (24.4) | 34 (25.2) |  |
| Q4 (highest) | 2643 (24.9) | 1328 (24.7) | 1288 (25.2) | 27 (20.0) |  |
| Unknown | 103 (1.0) | 52 (1.0) | 49 (1.0) | 2 (1.5) |  |
| **Marital status, n (%)** |  |  |  |  | <0.001 |
| Married or partnered | 7459 (70.2) | 3811 (71.0) | 3571 (69.7) | 77 (57.0) |  |
| Widowed | 2104 (19.8) | 1024 (19.1) | 1028 (20.1) | 52 (38.5) |  |
| Separated or divorced or single | 1061 (10.0) | 536 (10.0) | 519 (10.1) | 6 (4.4) |  |
| Unknown | 2 (0.0) | 0 (0.0) | 2 (0.0) | 0 (0.0) |  |
| **Current smoking status, n (%)** |  |  |  |  | 0.015 |
| No | 9052 (85.2) | 4544 (84.6) | 4404 (86.0) | 104 (77.0) |  |
| Yes | 1546 (14.5) | 811 (15.1) | 704 (13.8) | 31 (23.0) |  |
| Unknown | 28 (0.3) | 16 (0.3) | 12 (0.2) | 0 (0.0) |  |
| **Alcohol consumption, n (%)** |  |  |  |  | 0.004 |
| Less than weekly drinking | 6152 (57.9) | 3094 (57.6) | 2960 (57.8) | 98 (72.6) |  |
| Weekly drinking or more | 4077 (38.4) | 2075 (38.6) | 1972 (38.5) | 30 (22.2) |  |
| Unknown | 397 (3.7) | 202 (3.8) | 188 (3.7) | 7 (5.2) |  |
| **Physical activity, n (%)** |  |  |  |  | <0.001 |
| Physical inactive | 1755 (16.5) | 801 (14.9) | 917 (17.9) | 37 (27.4) |  |
| Physical active | 7177 (67.5) | 3713 (69.1) | 3398 (66.4) | 66 (48.9) |  |
| Unknown | 1694 (15.9) | 857 (16.0) | 805 (15.7) | 32 (23.7) |  |
| **Eyesight, n (%)** |  |  |  |  | <0.001 |
| Good or better | 7074 (66.6) | 3664 (68.2) | 3345 (65.3) | 65 (48.1) |  |
| Fair or worse | 3537 (33.3) | 1698 (31.6) | 1769 (34.6) | 70 (51.9) |  |
| Unknown | 15 (0.1) | 9 (0.2) | 6 (0.1) | 0 (0.0) |  |
| **Hearing, n (%)** |  |  |  |  | <0.001 |
| Good or better | 7203 (67.8) | 3758 (70.0) | 3374 (65.9) | 71 (52.6) |  |
| Fair or worse | 3417 (32.2) | 1611 (30.0) | 1742 (34.0) | 64 (47.4) |  |
| Unknown | 6 (0.1) | 2 (0.0) | 4 (0.1) | 0 (0.0) |  |
| **Stroke, n (%)** |  |  |  |  | 0.001 |
| No | 10075 (94.8) | 5138 (95.7) | 4812 (94.0) | 125 (92.6) |  |
| Yes | 546 (5.1) | 232 (4.3) | 304 (5.9) | 10 (7.4) |  |
| Unknown | 5 (0.0) | 1 (0.0) | 4 (0.1) | 0 (0.0) |  |
| **Arthritis, n (%)** |  |  |  |  | <0.001 |
| No | 5495 (51.7) | 3015 (56.1) | 2419 (47.2) | 61 (45.2) |  |
| Yes | 5126 (48.2) | 2353 (43.8) | 2699 (52.7) | 74 (54.8) |  |
| Unknown | 5 (0.0) | 3 (0.1) | 2 (0.0) | 0 (0.0) |  |
| **Memory-related diseases, n (%)** |  |  |  |  | 0.084 |
| No | 10471 (98.5) | 5310 (98.9) | 5029 (98.2) | 132 (97.8) |  |
| Yes | 150 (1.4) | 59 (1.1) | 88 (1.7) | 3 (2.2) |  |
| Unknown | 5 (0.0) | 2 (0.0) | 3 (0.1) | 0 (0.0) |  |
| **Number of other comorbidities, n (%)** |  |  |  |  | <0.001 |
| 0 | 3599 (33.9) | 1914 (35.6) | 1634 (31.9) | 51 (37.8) |  |
| 1 | 3891 (36.6) | 2020 (37.6) | 1828 (35.7) | 43 (31.9) |  |
| ≥ 2 | 3091 (29.1) | 1413 (26.3) | 1637 (32.0) | 41 (30.4) |  |
| Unknown | 45 (0.4) | 24 (0.4) | 21 (0.4) | 0 (0.0) |  |

Abbreviations: CHARLS, China Health and Retirement Longitudinal Study; ELSA, English Longitudinal Study on Ageing; HRS, US Health and Retirement Study.

**Table F.** Differences in physical function, depressive symptoms, and cognitive performance before and after falls

| **Year** | **No. of no-falls** | **No. of falls** | **Physical function Difference (95% CI)** | **Depressive symptoms Difference (95% CI)** | **Cognitive performance Difference (95% CI)** |
| --- | --- | --- | --- | --- | --- |
| < -7 | 564 | 569 | -0.02 (-0.11, 0.07) | -0.06 (-0.17, 0.05) | 0.01 (-0.10, 0.13) |
| -7 ~ < -5 | 1271 | 1281 | -0.06 (-0.13, 0.01) | **-0.15 (-0.23, -0.08)** | -0.03 (-0.11, 0.04) |
| -5 ~ < -3 | 2639 | 2707 | **-0.14 (-0.19, -0.09)** | **-0.17 (-0.22, -0.12)** | **-0.07 (-0.12, -0.01)** |
| -3 ~ < -1 | 5300 | 5418 | **-0.15 (-0.19, -0.11)** | **-0.20 (-0.24, -0.17)** | **-0.06 (-0.10, -0.02)** |
| -1 ~ < 1 | 5310 | 5499 | **-0.38 (-0.44, -0.33)** | **-0.29 (-0.33, -0.25)** | **-0.09 (-0.13, -0.04)** |
| 1 ~ < 3 | 3782 | 3955 | **-0.47 (-0.54, -0.40)** | **-0.27 (-0.31, -0.22)** | **-0.06 (-0.11, -0.01)** |
| 3 ~ < 5 | 2523 | 2680 | **-0.44 (-0.53, -0.36)** | **-0.27 (-0.33, -0.21)** | -0.03 (-0.10, 0.03) |
| 5 ~ | 2179 | 2369 | **-0.55 (-0.64, -0.45)** | **-0.25 (-0.31, -0.18)** | **-0.10 (-0.17, -0.03)** |

The difference was calculated as the mean of each measure in the falls group minus that in the no-falls group;

**Bolded text indicates statistically significant after FDR-correction**;

Abbreviations: CI, confidence interval.

**Table G.** Differences in physical function, depressive symptoms, and cognitive performance before and after falls with injuries

| **Year** | **No. of no-falls** | **No. of falls without injuries** | **No. of falls with injuries** | **Physical function Difference (95% CI)** | | **Depressive symptoms Difference (95% CI)** | | **Cognitive performance Difference (95% CI)** | |
| --- | --- | --- | --- | --- | --- | --- | --- | --- | --- |
|  |  |  |  | No-falls vs. falls without injuries | No-falls vs. falls with injuries | No-falls vs. falls without injuries | No-falls vs. falls with injuries | No-falls vs. falls without injuries | No-falls vs. falls with injuries |
| < -7 | 564 | 380 | 187 | -0.00 (-0.10, 0.09) | -0.07 (-0.20, 0.07) | -0.04 (-0.16, 0.08) | -0.11 (-0.27, 0.06) | -0.05 (-0.17, 0.08) | 0.15 (-0.03, 0.32) |
| -7 ~ < -5 | 1271 | 805 | 431 | -0.04 (-0.12, 0.04) | -0.07 (-0.18, 0.03) | **-0.15 (-0.23, -0.06)** | **-0.15 (-0.26, -0.05)** | -0.02 (-0.10, 0.07) | -0.05 (-0.16, 0.06) |
| -5 ~ < -3 | 2639 | 1650 | 872 | **-0.13 (-0.19, -0.07)** | **-0.14 (-0.22, -0.07)** | **-0.14 (-0.20, -0.08)** | **-0.21 (-0.29, -0.14)** | -0.05 (-0.11, 0.01) | **-0.10 (-0.18, -0.02)** |
| -3 ~ < -1 | 5300 | 3097 | 1757 | **-0.13 (-0.18, -0.09)** | **-0.17 (-0.23, -0.11)** | **-0.16 (-0.20, -0.11)** | **-0.24 (-0.30, -0.18)** | -0.04 (-0.08, 0.01) | **-0.13 (-0.19, -0.07)** |
| -1 ~ < 1 | 5310 | 3134 | 1796 | **-0.28 (-0.34, -0.21)** | **-0.58 (-0.67, -0.48)** | **-0.23 (-0.27, -0.18)** | **-0.37 (-0.43, -0.30)** | **-0.07 (-0.12, -0.02)** | **-0.13 (-0.20, -0.07)** |
| 1 ~ < 3 | 3782 | 2184 | 1251 | **-0.40 (-0.48, -0.32)** | **-0.49 (-0.60, -0.38)** | **-0.19 (-0.25, -0.13)** | **-0.32 (-0.39, -0.25)** | -0.04 (-0.10, 0.02) | -0.09 (-0.16, -0.01) |
| 3 ~ < 5 | 2523 | 1456 | 822 | **-0.32 (-0.42, -0.22)** | **-0.45 (-0.58, -0.32)** | **-0.17 (-0.24, -0.11)** | **-0.35 (-0.44, -0.25)** | 0.05 (-0.02, 0.13) | -0.15 (-0.24, -0.05) |
| 5 ~ | 2179 | 1151 | 830 | **-0.37 (-0.48, -0.25)** | **-0.59 (-0.73, -0.44)** | **-0.15 (-0.22, -0.07)** | **-0.31 (-0.41, -0.22)** | -0.04 (-0.12, 0.05) | **-0.14 (-0.26, -0.03)** |

The difference was calculated as the mean of each measure in the falls group minus that in the no-fall group;

**Bolded text indicates statistically significant after FDR-correction**;

Abbreviations: CI, confidence interval.

**Table H.** Differences in physical function, depressive symptoms, and cognitive performance before and after recurrent falls

| **Year** | **No. of no-falls** | **No. of single fall** | **No. of multiple falls** | **Physical function Difference (95% CI)** | | **Depressive symptoms Difference (95% CI)** | | **Cognitive performance Difference (95% CI)** | |
| --- | --- | --- | --- | --- | --- | --- | --- | --- | --- |
|  |  |  |  | No-falls vs. single fall | No-falls vs. multiple falls | No-falls vs. single fall | No-falls vs. multiple falls | No-falls vs. single fall | No-falls vs. multiple falls |
| < -7 | 314 | 209 | 101 | 0.07 (-0.07, 0.20) | -0.08 (-0.30, 0.14) | -0.08 (-0.24, 0.09) | -0.08 (-0.30, 0.15) | 0.01 (-0.15, 0.16) | -0.17 (-0.38, 0.03) |
| -7 ~ < -5 | 848 | 536 | 270 | 0.00 (-0.10, 0.10) | -0.07 (-0.20, 0.06) | **-0.11 (-0.21, -0.02)** | **-0.26 (-0.39, -0.12)** | -0.00 (-0.10, 0.09) | **-0.21 (-0.33, -0.09)** |
| -5 ~ < -3 | 1813 | 1007 | 656 | -0.04 (-0.11, 0.03) | **-0.16 (-0.25, -0.08)** | **-0.07 (-0.15, 0.00)** | **-0.19 (-0.28, -0.10)** | -0.02 (-0.09, 0.05) | **-0.17 (-0.26, -0.08)** |
| -3 ~ < -1 | 3901 | 1897 | 1509 | -0.03 (-0.08, 0.03) | **-0.23 (-0.30, -0.16)** | **-0.09 (-0.14, -0.04)** | **-0.31 (-0.38, -0.25)** | 0.04 (-0.02, 0.09) | **-0.18 (-0.24, -0.12)** |
| -1 ~ < 1 | 3862 | 1902 | 1517 | **-0.08 (-0.15, -0.01)** | **-0.68 (-0.79, -0.57)** | **-0.12 (-0.18, -0.07)** | **-0.41 (-0.48, -0.34)** | -0.03 (-0.09, 0.03) | **-0.24 (-0.31, -0.17)** |
| 1 ~ < 3 | 2887 | 1394 | 1114 | **-0.12 (-0.20, -0.04)** | **-0.83 (-0.97, -0.69)** | **-0.10 (-0.16, -0.04)** | **-0.37 (-0.45, -0.29)** | -0.01 (-0.08, 0.06) | **-0.16 (-0.24, -0.08)** |
| 3 ~ < 5 | 2007 | 941 | 795 | -0.09 (-0.18, 0.01) | **-0.70 (-0.85, -0.55)** | **-0.10 (-0.18, -0.02)** | **-0.32 (-0.41, -0.23)** | 0.06 (-0.02, 0.15) | -0.11 (-0.21, -0.02) |
| 5 ~ | 1127 | 509 | 433 | **-0.13 (-0.26, -0.00)** | **-0.82 (-1.03, -0.61)** | **-0.14 (-0.24, -0.04)** | **-0.36 (-0.48, -0.24)** | -0.07 (-0.18, 0.05) | **-0.16 (-0.29, -0.03)** |

The difference was calculated as the mean of each measure in the falls group minus that in the no-fall group;

**Bolded text indicates statistically significant after FDR-correction**;

Abbreviations: CI, confidence interval.

**Table I.** Differences in physical function, depressive symptoms, and cognitive performance before and after falls with hip fracture

| **Year** | **No. of no-falls** | **No. of falls without hip fracture** | **No. of falls with hip fracture** | **Physical function Difference (95% CI)** | | **Depressive symptoms Difference (95% CI)** | | **Cognitive performance Difference (95% CI)** | |
| --- | --- | --- | --- | --- | --- | --- | --- | --- | --- |
|  |  |  |  | No-falls vs. fall without hip fracture | No-falls vs. fall with hip fracture | No-falls vs. fall without hip fracture | No-falls vs. fall with hip fracture | No-falls vs. fall without hip fracture | No-falls vs. fall with hip fracture |
| < -7 | 549 | 532 | 17 | -0.03 (-0.12, 0.06) | NA | -0.07 (-0.19, 0.04) | NA | 0.01 (-0.11, 0.12) | NA |
| -7 ~ < -5 | 1261 | 1203 | 34 | -0.06 (-0.13, 0.01) | -0.07 (-0.29, 0.16) | **-0.16 (-0.23, -0.08)** | -0.06 (-0.37, 0.25) | -0.03 (-0.10, 0.05) | -0.28 (-0.63, 0.08) |
| -5 ~ < -3 | 2589 | 2537 | 74 | **-0.14 (-0.19, -0.09)** | **-0.27 (-0.48, -0.05)** | **-0.16 (-0.21, -0.11)** | **-0.37 (-0.63, -0.11)** | -0.05 (-0.11, 0.00) | **-0.57 (-0.82, -0.32)** |
| -3 ~ < -1 | 5179 | 5053 | 131 | **-0.14 (-0.18, -0.10)** | **-0.48 (-0.77, -0.20)** | **-0.20 (-0.24, -0.16)** | **-0.43 (-0.67, -0.19)** | **-0.05 (-0.09, -0.01)** | **-0.37 (-0.57, -0.17)** |
| -1 ~ < 1 | 5190 | 5124 | 135 | **-0.35 (-0.40, -0.29)** | **-1.78 (-2.28, -1.28)** | **-0.28 (-0.32, -0.24)** | **-0.66 (-0.91, -0.41)** | **-0.07 (-0.12, -0.03)** | **-0.51 (-0.73, -0.30)** |
| 1 ~ < 3 | 3700 | 3690 | 74 | **-0.42 (-0.49, -0.35)** | **-1.59 (-2.22, -0.96)** | **-0.27 (-0.32, -0.22)** | -0.27 (-0.59, 0.04) | -0.05 (-0.10, 0.00) | **-0.56 (-0.83, -0.28)** |
| 3 ~ < 5 | 2476 | 2504 | 56 | **-0.38 (-0.46, -0.30)** | **-1.39 (-2.08, -0.70)** | **-0.26 (-0.32, -0.20)** | **-0.50 (-0.87, -0.12)** | -0.01 (-0.08, 0.05) | **-0.59 (-0.91, -0.27)** |
| 5 ~ | 2122 | 2198 | 41 | **-0.48 (-0.57, -0.39)** | **-1.56 (-2.59, -0.52)** | **-0.24 (-0.31, -0.17)** | **0.04 (-0.40, 0.48)** | **-0.09 (-0.16, -0.01)** | **-0.55 (-1.13, 0.02)** |

The difference was calculated as the mean of each measure in the falls group minus that in the no-fall group;

**Bolded text indicates statistically significant after FDR-correction**;

Abbreviations: CI, confidence interval; NA, not applicable.

**Table J.** Estimated changes in physical function, depressive symptoms, and cognitive performance before and after falls by cohort

|  | **CHARLS β (95% CI)** | **ELSA β (95% CI)** | **HRS β (95% CI)** |
| --- | --- | --- | --- |
| **Physical function** | | | |
| Falls | **-0.381 (-0.468, -0.294)** | **-0.109 (-0.176, -0.042)** | **-0.270 (-0.342, -0.197)** |
| Falls × pre_time | **-0.068 (-0.087, -0.049)** | -0.011 (-0.028, 0.006) | **-0.081 (-0.099, -0.063)** |
| Falls × post_time | **-0.029 (-0.049, -0.010)** | **-0.034 (-0.050, -0.018)** | **-0.076 (-0.094, -0.058)** |
| **Depressive symptoms** | | | |
| Falls | **-0.251 (-0.315, -0.186)** | **-0.180 (-0.235, -0.126)** | **-0.216 (-0.269, -0.162)** |
| Falls × pre_time | **-0.023 (-0.037, -0.009)** | -0.006 (-0.020, 0.008) | **-0.025 (-0.039, -0.012)** |
| Falls × post_time | 0.009 (-0.006, 0.023) | -0.003 (-0.017, 0.010) | -0.010 (-0.024, 0.003) |
| **Cognitive performance** | | | |
| Falls | -0.038 (-0.107, 0.031) | -0.006 (-0.060, 0.049) | -0.034 (-0.084, 0.015) |
| Falls × pre_time | -0.001 (-0.016, 0.013) | -0.005 (-0.018, 0.008) | **-0.015 (-0.026, -0.003)** |
| Falls × post_time | -0.002 (-0.020, 0.015) | -0.007 (-0.020, 0.006) | -0.012 (-0.024, 0.000) |

Models were adjusted age, sex, education level, total household wealth, marital status, current smoking status, current alcohol consumption, physical activity, stroke, arthritis, memory-related diseases, eyesight, hearing, and number of other comorbidities;

**Bolded text indicates statistically significant;**

Abbreviations: CHARLS, China Health and Retirement Longitudinal Study; ELSA, English longitudinal study of ageing; HRS, Health and Retirement Study; CI, confidence interval.

**Table K.** Estimated changes in physical function, depressive symptoms, and cognitive performance before and after injuries from falls by cohort

|  | **CHARLS β (95% CI)** | **ELSA β (95% CI)** | **HRS β (95% CI)** |
| --- | --- | --- | --- |
| **Physical function** | | | |
| Falls without injuries | **-0.333 (-0.439, -0.227)** | **-0.078 (-0.153, -0.003)** | **-0.226 (-0.308, -0.144)** |
| Falls with injuries | **-0.484 (-0.595, -0.373)** | **-0.149 (-0.248, -0.050)** | **-0.409 (-0.514, -0.305)** |
| Falls without injuries × pre_time | **-0.054 (-0.076, -0.031)** | -0.004 (-0.023, 0.014) | **-0.067 (-0.086, -0.047)** |
| Falls with injuries × pre_time | **-0.091 (-0.115, -0.067)** | -0.019 (-0.044, 0.006) | **-0.122 (-0.148, -0.097)** |
| Falls without injuries × post_time | -0.012 (-0.039, 0.009) | **-0.031 (-0.049, -0.012)** | **-0.044 (-0.065, -0.024)** |
| Falls with injuries × post_time | -0.007 (-0.032, 0.017) | -0.023 (-0.047, 0.001) | **-0.076 (-0.102, -0.051)** |
| **Depressive symptoms** | | | |
| Falls without injuries | **-0.222 (-0.300, -0.144)** | **-0.148 (-0.209, -0.086)** | **-0.153 (-0.214, -0.093)** |
| Falls with injuries | **-0.259 (-0.342, -0.176)** | **-0.229 (-0.310, -0.148)** | **-0.291 (-0.368, -0.213)** |
| Falls without injuries × pre_time | **-0.023 (-0.039, -0.007)** | 0.001 (-0.014, 0.017) | -0.013 (-0.028, 0.002) |
| Falls with injuries × pre_time | **-0.025 (-0.042, -0.007)** | -0.020 (-0.040, 0.001) | **-0.042 (-0.062, -0.023)** |
| Falls without injuries × post_time | **0.019 (0.001, 0.037)** | 0.001 (-0.015, 0.016) | -0.008 (-0.024, 0.008) |
| Falls with injuries × post_time | 0.002 (-0.016, 0.021) | 0.007 (-0.013, 0.027) | -0.015 (-0.035, 0.004) |
| **Cognitive performance** | | | |
| Falls without injuries | -0.054 (-0.139, 0.032) | -0.029 (-0.091, 0.033) | -0.028 (-0.085, 0.029) |
| Falls with injuries | -0.054 (-0.145, 0.037) | 0.001 (-0.081, 0.083) | **-0.085 (-0.159, -0.012)** |
| Falls without injuries × pre_time | -0.003 (-0.020, 0.014) | 0.000 (-0.015, 0.014) | -0.012 (-0.025, 0.001) |
| Falls with injuries × pre_time | -0.002 (-0.021, 0.018) | -0.015 (-0.035, 0.004) | **-0.021 (-0.038, -0.004)** |
| Falls without injuries × post_time | 0.006 (-0.016, 0.028) | 0.003 (-0.012, 0.017) | -0.007 (-0.020, 0.007) |
| Falls with injuries × post_time | -0.001 (-0.025, 0.022) | -0.019 (-0.037, 0.000) | -0.017 (-0.035, 0.000) |

Models were adjusted age, sex, education level, total household wealth, marital status, current smoking status, current alcohol consumption, physical activity, stroke, arthritis, memory-related diseases, eyesight, hearing, and number of other comorbidities;

**Bolded text indicates statistically significant;**

Abbreviations: CHARLS, China Health and Retirement Longitudinal Study; ELSA, English longitudinal study of ageing; HRS, Health and Retirement Study; CI, confidence interval.

**Table L.** Estimated changes in physical function, depressive symptoms, and cognitive performance before and after recurrent falls

|  | **ELSA β (95% CI)** | **HRS β (95% CI)** |
| --- | --- | --- |
| **Physical function** |  |  |
| Single fall | 0.004 (-0.073, 0.080) | **-0.106 (-0.199, -0.013)** |
| Multiple falls | **-0.375 (-0.473, -0.276)** | **-0.487 (-0.577, -0.398)** |
| Single fall × pre_time | 0.009 (-0.010, 0.027) | **-0.042 (-0.063, -0.020)** |
| Multiple falls × pre_time | **-0.068 (-0.093, -0.042)** | **-0.135 (-0.157, -0.113)** |
| Single fall × post_time | -0.006 (-0.025, 0.013) | -0.019 (-0.043, 0.005) |
| Multiple falls × post_time | **-0.051 (-0.074, -0.028)** | **-0.083 (-0.105, -0.061)** |
| **Depressive symptoms** |  |  |
| Single fall | **-0.079 (-0.140, -0.018)** | **-0.118 (-0.187, -0.050)** |
| Multiple falls | **-0.356 (-0.435, -0.278)** | **-0.263 (-0.330, -0.197)** |
| Single fall × pre_time | 0.000 (-0.016, 0.015) | -0.012 (-0.028, 0.004) |
| Multiple falls × pre_time | -0.017 (-0.038, 0.004) | **-0.031 (-0.048, -0.014)** |
| Single fall × post_time | -0.002 (-0.018, 0.013) | -0.008 (-0.026, 0.010) |
| Multiple falls × post_time | 0.005 (-0.014, 0.025) | -0.004 (-0.021, 0.013) |
| **Cognitive performance** |  |  |
| Single fall | 0.002 (-0.062, 0.065) | 0.042 (-0.024, 0.107) |
| Multiple falls | **-0.111 (-0.192, -0.029)** | **-0.133 (-0.196, -0.069)** |
| Single fall × pre_time | -0.005 (-0.019, 0.010) | -0.007 (-0.021, 0.008) |
| Multiple falls × pre_time | -0.013 (-0.033, 0.007) | **-0.027 (-0.042, -0.012)** |
| Single fall × post_time | -0.001 (-0.016, 0.013) | -0.010 (-0.027, 0.006) |
| Multiple falls × post_time | -0.002 (-0.020, 0.017) | -0.010 (-0.025, 0.006) |

Models were adjusted age, sex, education level, total household wealth, marital status, current smoking status, current alcohol consumption, physical activity, stroke, arthritis, memory-related diseases, eyesight, hearing, and number of other comorbidities;

**Bolded text indicates statistically significant;**

Abbreviations: ELSA, English longitudinal study of ageing; HRS, Health and Retirement Study; CI, confidence interval.

**Table M.** Estimated changes in physical function, depressive symptoms, and cognitive performance before and after falls with hip fracture

|  | **CHARLS β (95% CI)** | **ELSA β (95% CI)** | **HRS β (95% CI)** |
| --- | --- | --- | --- |
| **Physical function** | | | |
| Falls without hip fracture | **-0.336 (-0.422, -0.249)** | **-0.110 (-0.177, -0.043)** | **-0.261 (-0.333, -0.189)** |
| Falls with hip fracture | **-1.344 (-1.669, -1.019)** | **-0.575 (-0.999, -0.150)** | **-1.454 (-1.765, -1.142)** |
| Falls without hip fracture × pre_time | **-0.061 (-0.080, -0.042)** | -0.010 (-0.027, 0.007) | **-0.076 (-0.094, -0.058)** |
| Falls with hip fracture × pre_time | **-0.329 (-0.412, -0.247)** | -0.105 (-0.211, 0.001) | **-0.383 (-0.465, -0.300)** |
| Falls without hip fracture × post_time | **-0.029 (-0.049, -0.010)** | **-0.026 (-0.042, -0.010)** | **-0.060 (-0.078, -0.043)** |
| Falls with hip fracture × post_time | 0.082 (-0.001, 0.165) | -0.098 (-0.223, 0.027) | **-0.159 (-0.250, -0.068)** |
| **Depressive symptoms** | | | |
| Falls without hip fracture | **-0.235 (-0.302, -0.169)** | **-0.194 (-0.249, -0.139)** | **-0.209 (-0.263, -0.155)** |
| Falls with hip fracture | **-0.581 (-0.843, -0.318)** | -0.211 (-0.564, 0.143) | **-0.474 (-0.717, -0.231)** |
| Falls without hip fracture × pre_time | **-0.021 (-0.035, -0.007)** | -0.010 (-0.024, 0.004) | **-0.023 (-0.036, -0.009)** |
| Falls with hip fracture × pre_time | **-0.108 (-0.173, -0.044)** | -0.046 (-0.134, 0.041) | -0.045 (-0.112, 0.021) |
| Falls without hip fracture × post_time | 0.006 (-0.009, 0.021) | -0.001 (-0.015, 0.013) | -0.014 (-0.027, 0.000) |
| Falls with hip fracture × post_time | **0.106 (0.037, 0.175)** | 0.088 (-0.020, 0.197) | 0.061 (-0.013, 0.134) |
| **Cognitive performance** | | | |
| Falls without hip fracture | -0.025 (-0.097, 0.046) | -0.015 (-0.071, -0.040) | -0.029 (-0.080, 0.021) |
| Falls with hip fracture | -0.261 (-0.550, 0.028) | -0.030 (-0.382, 0.322) | **-0.271 (-0.496, -0.046)** |
| Falls without hip fracture × pre_time | -0.001 (-0.016, 0.014) | -0.006 (-0.019, 0.007) | **-0.015 (-0.027, -0.003)** |
| Falls with hip fracture × pre_time | -0.013 (-0.085, 0.060) | -0.028 (-0.109, 0.055) | -0.045 (-0.102, 0.013) |
| Falls without hip fracture × post_time | -0.002 (-0.020, 0.016) | -0.006 (-0.019, 0.006) | -0.009 (-0.021, 0.003) |
| Falls with hip fracture × post_time | -0.034 (-0.115, 0.048) | 0.021 (-0.081, 0.122) | -0.055 (-0.120, 0.009) |

Models were adjusted age, sex, education level, total household wealth, marital status, current smoking status, current alcohol consumption, physical activity, stroke, arthritis, memory-related diseases, eyesight, hearing, and number of other comorbidities;

**Bolded text indicates statistically significant;**

Abbreviations: CHARLS, China Health and Retirement Longitudinal Study; ELSA, English longitudinal study of ageing; HRS, Health and Retirement Study; CI, confidence interval.

**Table N.** Subgroup analyses of estimated changes in physical function, depressive symptoms, and cognitive performance before and after falls

|  | **Physical function β (95% CI)** | ***P*-interaction** | **Depressive symptoms β (95% CI)** | ***P*-interaction** | **Cognitive performance β (95% CI)** | ***P*-interaction** |
| --- | --- | --- | --- | --- | --- | --- |
| **Age (< 75 years)** |  |  |  |  |  |  |
| Falls | **-0.236 (-0.282, -0.190)** |  | **-0.206 (-0.244, -0.167)** |  | **-0.043 (-0.082, -0.003)** |  |
| Falls × pre_time | **-0.042 (-0.053, -0.032)** |  | **-0.015 (-0.023, -0.006)** |  | -0.007 (-0.016, 0.001) |  |
| Falls × post_time | **-0.031 (-0.042, -0.021)** |  | 0.000 (-0.008, 0.009) |  | -0.003 (-0.012, 0.006) |  |
| **Age (≥ 75 years)** |  |  |  |  |  |  |
| Falls | **-0.321 (-0.420, -0.221)** | **0.050** | **-0.235 (-0.299, -0.171)** | 0.454 | -0.029 (-0.094, 0.037) | 0.669 |
| Falls × pre_time | **-0.095 (-0.123, -0.067)** | **<0.001** | **-0.028 (-0.046, -0.010)** | 0.186 | 0.002 (-0.014, 0.017) | 0.310 |
| Falls × post_time | **-0.077 (-0.104, -0.050)** | **<0.001** | -0.011 (-0.029, 0.007) | 0.261 | **-0.021 (-0.036, -0.005)** | 0.056 |
| **Male** |  |  |  |  |  |  |
| Falls | **-0.295 (-0.357, -0.233)** |  | **-0.212 (-0.257, -0.168)** |  | -0.023 (-0.069, 0.023) |  |
| Falls × pre_time | **-0.062 (-0.077, -0.047)** |  | **-0.018 (-0.028, -0.007)** |  | -0.002 (-0.012, 0.008) |  |
| Falls × post_time | **-0.045 (-0.060, -0.030)** |  | 0.006 (-0.005, 0.017) |  | -0.005 (-0.017, 0.006) |  |
| **Female** |  |  |  |  |  |  |
| Falls | **-0.217 (-0.278, -0.156)** | 0.134 | **-0.224 (-0.271, -0.176)** | 0.739 | -0.034 (-0.081, 0.012) | 0.591 |
| Falls × pre_time | **-0.050 (-0.065, -0.036)** | 0.257 | **-0.019 (-0.031, -0.007)** | 0.862 | **-0.011 (-0.022, -0.001)** | 0.206 |
| Falls × post_time | **-0.048 (-0.062, -0.034)** | 0.800 | -0.008 (-0.020, 0.003) | 0.072 | -0.011 (-0.021, 0.000) | 0.578 |
| **Primary education level** |  |  |  |  |  |  |
| Falls | **-0.312 (-0.386, -0.238)** |  | **-0.290 (-0.344, -0.236)** |  | **-0.055 (-0.109, 0.000)** |  |
| Falls × pre_time | **-0.066 (-0.083, -0.049)** |  | **-0.027 (-0.039, -0.015)** |  | -0.007 (-0.019, 0.005) |  |
| Falls × post_time | **-0.043 (-0.060, -0.026)** |  | 0.004 (-0.009, 0.017) |  | -0.009 (-0.022, 0.005) |  |
| **Secondary education level** |  |  |  |  |  |  |
| Falls | **-0.216 (-0.280, -0.151)** | **0.015** | **-0.147 (-0.197, -0.097)** | **<0.001** | -0.019 (-0.068, 0.030) | 0.466 |
| Falls × pre_time | **-0.056 (-0.072, -0.040)** | 0.367 | -0.011 (-0.023, 0.002) | 0.068 | -0.006 (-0.017, 0.006) | 0.883 |
| Falls × post_time | **-0.058 (-0.073, -0.042)** | 0.217 | -0.008 (-0.020, 0.004) | 0.179 | **-0.013 (-0.024, -0.001)** | 0.600 |
| **Tertiary education level** |  |  |  |  |  |  |
| Falls | **-0.194 (-0.284, -0.103)** | **0.023** | **-0.168 (-0.253, -0.094)** | **0.022** | 0.003 (-0.076, 0.082) | 0.529 |
| Falls × pre_time | **-0.037 (-0.061, -0.014)** | 0.079 | -0.010 (-0.029, 0.009) | 0.191 | -0.014 (-0.033, 0.004) | 0.457 |
| Falls × post_time | -0.020 (-0.043, 0.002) | 0.190 | -0.002 (-0.020, -0.016) | 0.602 | -0.001 (-0.019, 0.018) | 0.550 |
| **Lower wealth** |  |  |  |  |  |  |
| Falls | **-0.288 (-0.359, -0.216)** |  | **-0.242 (-0.294, -0.190)** |  | 0.010 (-0.039, 0.059) |  |
| Falls × pre_time | **-0.064 (-0.081, -0.047)** |  | **-0.020 (-0.032, -0.008)** |  | -0.006 (-0.017, 0.005) |  |
| Falls × post_time | **-0.066 (-0.083, -0.049)** |  | -0.002 (-0.015, 0.010) |  | **-0.013 (-0.025, -0.002)** |  |
| **Upper wealth** |  |  |  |  |  |  |
| Falls | **-0.217 (-0.268, -0.166)** | **0.035** | **-0.190 (-0.231, -0.148)** | 0.057 | **-0.060 (-0.105, -0.014)** | **0.020** |
| Falls × pre_time | **-0.048 (-0.060, -0.035)** | 0.127 | **-0.018 (-0.028, -0.008)** | 0.758 | -0.007 (-0.018, 0.003) | 0.859 |
| Falls × post_time | **-0.030 (-0.043, -0.018)** | **0.001** | -0.002 (-0.012, 0.008) | 0.930 | -0.003 (-0.014, 0.007) | 0.217 |

Models were adjusted age, sex, cohort, education level, total household wealth, marital status, current smoking status, current alcohol consumption, physical activity, stroke, arthritis, memory-related diseases, eyesight, hearing, and number of other comorbidities;

**Bolded text indicates statistically significant.**

**Table O.** Subgroup analyses of estimated changes in physical function, depressive symptoms, and cognitive performance before and after injuries from falls

|  | **Physical function β (95% CI)** | ***P*-interaction** | **Depressive symptoms β (95% CI)** | ***P*-interaction** | **Cognitive performance β (95% CI)** | ***P*-interaction** |
| --- | --- | --- | --- | --- | --- | --- |
| **Age (< 75 years)** |  |  |  |  |  |  |
| Falls without injuries | **-0.181 (-0.234, -0.129)** |  | **-0.165 (-0.209, -0.120)** |  | **-0.052 (-0.097, -0.006)** |  |
| Falls with injuries | **-0.364 (-0.429, -0.298)** |  | **-0.244 (-0.300, -0.189)** |  | -0.048 (-0.106, 0.010) |  |
| Falls without injuries × pre_time | **-0.030 (-0.042, -0.019)** |  | -0.007 (-0.017, 0.003) |  | -0.005 (-0.015, 0.005) |  |
| Falls with injuries × pre_time | **-0.066 (-0.080, -0.051)** |  | **-0.027 (-0.040, -0.015)** |  | -0.012 (-0.024, 0.001) |  |
| Falls without injuries × post_time | **-0.022 (-0.035, -0.010)** |  | 0.006 (-0.004, 0.017) |  | 0.006 (-0.005, 0.016) |  |
| Falls with injuries × post_time | -0.011 (-0.025, 0.003) |  | -0.005 (-0.018, 0.007) |  | **-0.015 (-0.028, -0.001)** |  |
| **Age (≥ 75 years)** |  |  |  |  |  |  |
| Falls without injuries | **-0.275 (-0.392, -0.157)** | 0.065 | **-0.191 (-0.265, -0.116)** | 0.496 | -0.000 (-0.077, 0.077) | 0.187 |
| Falls with injuries | **-0.430 (-0.563, -0.296)** | 0.180 | **-0.296 (-0.382, -0.211)** | 0.415 | **-0.120 (-0.208, -0.032)** | 0.149 |
| Falls without injuries × pre_time | **-0.074 (-0.106, -0.043)** | **0.002** | **-0.024 (-0.044, -0.004)** | 0.130 | 0.004 (-0.014, 0.022) | 0.362 |
| Falls with injuries × pre_time | **-0.139 (-0.177, -0.102)** | **<0.001** | **-0.030 (-0.054, -0.006)** | 0.846 | -0.007 (-0.028, 0.015) | 0.659 |
| Falls without injuries × post_time | **-0.057 (-0.089, -0.024)** | **0.019** | -0.007 (-0.028, 0.015) | 0.287 | **-0.021 (-0.040, -0.002)** | **0.018** |
| Falls with injuries × post_time | **-0.064 (-0.099, -0.029)** | **<0.001** | -0.003 (-0.027, 0.020) | 0.896 | -0.007 (-0.029, 0.014) | 0.056 |
| **Male** |  |  |  |  |  |  |
| Falls without injuries | **-0.242 (-0.311, -0.173)** |  | **-0.200 (-0.250, -0.150)** |  | -0.031 (-0.083, 0.021) |  |
| Falls with injuries | **-0.438 (-0.530, -0.345)** |  | **-0.228 (-0.295, -0.161)** |  | -0.045 (-0.115, 0.025) |  |
| Falls without injuries × pre_time | **-0.047 (-0.064, -0.031)** |  | **-0.015 (-0.027, -0.004)** |  | -0.001 (-0.013, 0.010) |  |
| Falls with injuries × pre_time | **-0.095 (-0.117, -0.073)** |  | **-0.024 (-0.039, -0.008)** |  | -0.008 (-0.024, 0.008) |  |
| Falls without injuries × post_time | **-0.028 (-0.045, -0.011)** |  | **0.016 (0.004, 0.029)** |  | 0.006 (-0.007, 0.019) |  |
| Falls with injuries × post_time | **-0.042 (-0.065, -0.020)** |  | -0.006 (-0.022, 0.011) |  | -0.012 (-0.029, 0.005) |  |
| **Female** |  |  |  |  |  |  |
| Falls without injuries | **-0.173 (-0.245, -0.101)** | 0.235 | **-0.149 (-0.206, -0.092)** | 0.149 | -0.038 (-0.094, 0.017) | 0.734 |
| Falls with injuries | **-0.312 (-0.393, -0.230)** | 0.064 | **-0.289 (-0.353, -0.225)** | 0.171 | **-0.065 (-0.129, -0.002)** | 0.131 |
| Falls without injuries × pre_time | **-0.036 (-0.053, -0.019)** | 0.341 | -0.007 (-0.021, 0.006) | 0.375 | -0.007 (-0.020, 0.005) | 0.460 |
| Falls with injuries × pre_time | **-0.076 (-0.095, -0.057)** | 0.186 | **-0.031 (-0.046, -0.015)** | 0.250 | **-0.017 (-0.031, -0.002)** | 0.458 |
| Falls without injuries × post_time | **-0.034 (-0.051, -0.017)** | 0.617 | -0.008 (-0.022, 0.006) | **0.011** | -0.006 (-0.019, 0.007) | 0.228 |
| Falls with injuries × post_time | **-0.027 (-0.045, -0.008)** | 0.239 | -0.004 (-0.019, 0.012) | 0.878 | **-0.017 (-0.032, -0.002)** | 0.736 |
| **Primary education level** |  |  |  |  |  |  |
| Falls without injuries | **-0.234 (-0.322, -0.146)** |  | **-0.252 (-0.317, -0.188)** |  | -0.057 (-0.122, 0.008) |  |
| Falls with injuries | **-0.445 (-0.544, -0.347)** |  | **-0.299 (-0.372, -0.226)** |  | **-0.086 (-0.160, -0.012)** |  |
| Falls without injuries × pre_time | **-0.048 (-0.067, -0.029)** |  | **-0.023 (-0.037, -0.009)** |  | -0.007 (-0.020, 0.007) |  |
| Falls with injuries × pre_time | **-0.093 (-0.115, -0.071)** |  | **-0.029 (-0.045, -0.012)** |  | -0.008 (-0.024, 0.009) |  |
| Falls without injuries × post_time | **-0.033 (-0.054, -0.013)** |  | 0.014 (-0.002, 0.030) |  | -0.010 (-0.026, 0.006) |  |
| Falls with injuries × post_time | -0.021 (-0.043, 0.001) |  | -0.008 (-0.025, 0.009) |  | -0.001 (-0.019, 0.018) |  |
| **Secondary education level** |  |  |  |  |  |  |
| Falls without injuries | **-0.200 (-0.272, -0.128)** | 0.287 | **-0.094 (-0.151, -0.037)** | **<0.001** | -0.037 (-0.093, 0.020) | 0.832 |
| Falls with injuries | **-0.274 (-0.367, -0.181)** | **0.006** | **-0.240 (-0.313, -0.167)** | 0.183 | -0.032 (-0.104, 0.041) | 0.115 |
| Falls without injuries × pre_time | **-0.045 (-0.062, -0.028)** | 0.782 | 0.001 (-0.013, 0.015) | **0.015** | -0.003 (-0.016, 0.010) | 0.713 |
| Falls with injuries × pre_time | **-0.077 (-0.100, -0.054)** | 0.298 | **-0.031 (-0.049, -0.012)** | 0.885 | -0.014 (-0.031, 0.003) | 0.601 |
| Falls without injuries × post_time | **-0.035 (-0.052, -0.017)** | 0.927 | -0.002 (-0.017, 0.012) | 0.117 | 0.001 (-0.013, 0.014) | 0.336 |
| Falls with injuries × post_time | **-0.054 (-0.076, -0.032)** | **0.048** | 0.001 (-0.018, 0.019) | 0.476 | **-0.026 (-0.043, -0.009)** | **0.043** |
| **Tertiary education level** |  |  |  |  |  |  |
| Falls without injuries | **-0.136 (-0.236, -0.035)** | 0.083 | **-0.159 (-0.243, -0.076)** | 0.126 | 0.019 (-0.070, 0.108) | 0.468 |
| Falls with injuries | **-0.384 (-0.517, -0.251)** | 0.289 | **-0.208 (-0.319, -0.096)** | 0.277 | -0.034 (-0.153, 0.085) | 0.747 |
| Falls without injuries × pre_time | **-0.029 (-0.054, -0.004)** | 0.276 | -0.007 (-0.027, 0.013) | 0.250 | -0.006 (-0.027, 0.014) | 0.991 |
| Falls with injuries × pre_time | **-0.068 (-0.102, -0.034)** | 0.285 | -0.018 (-0.046, 0.009) | 0.597 | **-0.032 (-0.060, -0.004)** | 0.121 |
| Falls without injuries × post_time | -0.006 (-0.032, 0.019) | 0.151 | -0.002 (-0.023, 0.019) | 0.226 | 0.003 (-0.019, 0.024) | 0.405 |
| Falls with injuries × post_time | 0.005 (-0.029, 0.039) | 0.311 | 0.002 (-0.026, 0.029) | 0.598 | -0.014 (-0.043, 0.015) | 0.459 |
| **Lower wealth** |  |  |  |  |  |  |
| Falls without injuries | **-0.232 (-0.314, -0.149)** |  | **-0.182 (-0.242, -0.121)** |  | -0.001 (-0.059, 0.056) |  |
| Falls with injuries | **-0.390 (-0.488, -0.292)** |  | **-0.296 (-0.368, -0.224)** |  | -0.012 (-0.081, 0.057) |  |
| Falls without injuries × pre_time | **-0.048 (-0.067, -0.029)** |  | -0.013 (-0.027, 0.001) |  | -0.004 (-0.017, 0.008) |  |
| Falls with injuries × pre_time | **-0.093 (-0.116, -0.070)** |  | **-0.028 (-0.045, -0.011)** |  | -0.012 (-0.028, 0.003) |  |
| Falls without injuries × post_time | **-0.038 (-0.058, -0.018)** |  | 0.006 (-0.010, 0.021) |  | -0.010 (-0.024, 0.004) |  |
| Falls with injuries × post_time | **-0.067 (-0.090, -0.044)** |  | -0.010 (-0.028, 0.007) |  | **-0.019 (-0.035, -0.002)** |  |
| **Upper wealth** |  |  |  |  |  |  |
| Falls without injuries | **-0.178 (-0.236, -0.119)** | 0.166 | **-0.163 (-0.211, -0.116)** | 0.479 | **-0.054 (-0.107, -0.002)** | 0.107 |
| Falls with injuries | **-0.345 (-0.418, -0.272)** | 0.254 | **-0.228 (-0.287, -0.169)** | 0.062 | **-0.101 (-0.167, -0.035)** | 0.059 |
| Falls without injuries × pre_time | **-0.036 (-0.050, -0.022)** | 0.324 | -0.010 (-0.021, 0.001) | 0.700 | -0.005 (-0.016, 0.007) | 0.951 |
| Falls with injuries × pre_time | **-0.074 (-0.091, -0.056)** | 0.197 | **-0.031 (-0.045, -0.016)** | 0.820 | -0.012 (-0.027, 0.003) | 0.982 |
| Falls without injuries × post_time | **-0.025 (-0.039, -0.010)** | 0.295 | 0.003 (-0.009, 0.015) | 0.741 | 0.009 (-0.003, 0.021) | 0.053 |
| Falls with injuries × post_time | -0.001 (-0.018, 0.016) | **<0.001** | 0.001 (-0.013, 0.016) | 0.323 | -0.013 (-0.028, 0.003) | 0.660 |

Models were adjusted age, sex, cohort, education level, total household wealth, marital status, current smoking status, current alcohol consumption, physical activity, stroke, arthritis, memory-related diseases, eyesight, hearing, and number of other comorbidities;

**Bolded text indicates statistically significant.**

**Table P.** Subgroup analyses of estimated changes in physical function, depressive symptoms, and cognitive performance before and after recurrent falls

|  | **Physical function β (95% CI)** | ***P*-interaction** | **Depressive symptoms β (95% CI)** | ***P*-interaction** | **Cognitive performance β (95% CI)** | ***P*-interaction** |
| --- | --- | --- | --- | --- | --- | --- |
| **Age (< 75 years)** |  |  |  |  |  |  |
| Single fall | -0.035 (-0.097, -0.027) |  | -0.053 (-0.108, 0.002) |  | -0.020 (-0.075, 0.036) |  |
| Multiple falls | **-0.327 (-0.398, -0.256)** |  | **-0.276 (-0.339, -0.213)** |  | **-0.095 (-0.159, -0.032)** |  |
| Single fall × pre_time | 0.001 (-0.012, 0.014) |  | 0.002 (-0.011, 0.014) |  | -0.009 (-0.021, 0.003) |  |
| Multiple falls × pre_time | **-0.074 (-0.091, -0.057)** |  | **-0.023 (-0.039, -0.007)** |  | -0.012 (-0.027, 0.002) |  |
| Single fall× post_time | -0.008 (-0.022, 0.006) |  | -0.007 (-0.021, 0.006) |  | -0.003 (-0.016, 0.010) |  |
| Multiple falls × post_time | **-0.048 (-0.064, -0.032)** |  | 0.002 (-0.013, 0.017) |  | 0.000 (-0.014, 0.015) |  |
| **Age (≥ 75 years)** |  |  |  |  |  |  |
| Single fall | -0.091 (-0.219, 0.038) | 0.330 | **-0.164 (-0.246, -0.081)** | **0.028** | 0.058 (-0.027, 0.142) | 0.208 |
| Multiple falls | **-0.648 (-0.779, -0.516)** | **<0.001** | **-0.329 (-0.415, -0.244)** | 0.286 | **-0.200 (-0.287, -0.113)** | 0.150 |
| Single fall × pre_time | **-0.044 (-0.079, -0.010)** | **0.005** | -0.019 (-0.041, 0.004) | 0.111 | 0.006 (-0.013, 0.025) | 0.173 |
| Multiple falls × pre_time | **-0.164 (-0.201, -0.127)** | **<0.001** | **-0.030 (-0.054, -0.006)** | 0.582 | -0.017 (-0.038, 0.004) | 0.803 |
| Single fall× post_time | -0.009 (-0.044, 0.027) | 0.950 | 0.004 (-0.020, 0.027) | 0.398 | -0.013 (-0.033, 0.007) | 0.397 |
| Multiple falls × post_time | **-0.102 (-0.138, -0.066)** | **0.001** | -0.007 (-0.031, 0.018) | 0.546 | -0.016 (-0.038, 0.005) | 0.238 |
| **Male** |  |  |  |  |  |  |
| Single fall | **-0.086 (-0.169, -0.003)** |  | **-0.091 (-0.153, -0.028)** |  | -0.031 (-0.098, 0.035) |  |
| Multiple falls | **-0.454 (-0.542, -0.366)** |  | **-0.261 (-0.327, -0.195)** |  | **-0.109 (-0.180, -0.039)** |  |
| Single fall × pre_time | -0.016 (-0.036, 0.003) |  | -0.005 (-0.020, 0.009) |  | -0.007 (-0.021, 0.008) |  |
| Multiple falls × pre_time | **-0.112 (-0.135, -0.090)** |  | **-0.025 (-0.042, -0.008)** |  | **-0.021 (-0.037, -0.004)** |  |
| Single fall× post_time | -0.012 (-0.033, 0.010) |  | -0.000 (-0.017, 0.016) |  | 0.001 (-0.015, 0.018) |  |
| Multiple falls × post_time | **-0.067 (-0.090, -0.045)** |  | 0.002 (-0.015, 0.019) |  | 0.002 (-0.015, 0.019) |  |
| **Female** |  |  |  |  |  |  |
| Single fall | -0.008 (-0.093, 0.078) | 0.256 | **-0.093 (-0.159, -0.027)** | 0.774 | 0.059 (-0.004, 0.121) | **0.032** |
| Multiple falls | **-0.440 (-0.538, -0.343)** | 0.673 | **-0.340 (-0.415, -0.265)** | 0.100 | **-0.137 (-0.208, -0.066)** | 0.321 |
| Single fall × pre_time | -0.010 (-0.030, 0.011) | 0.666 | -0.004 (-0.021, 0.012) | 0.943 | -0.005 (-0.020, 0.009) | 0.879 |
| Multiple falls × pre_time | **-0.116 (-0.141, -0.092)** | 0.801 | **-0.029 (-0.049, -0.009)** | 0.745 | **-0.020 (-0.038, -0.003)** | 0.972 |
| Single fall× post_time | -0.010 (-0.030, 0.010) | 0.884 | -0.006 (-0.023, 0.010) | 0.606 | -0.012 (-0.027, 0.002) | 0.238 |
| Multiple falls × post_time | **-0.070 (-0.093, -0.048)** | 0.962 | -0.003 (-0.022, 0.016) | 0.698 | -0.011 (-0.028, 0.005) | 0.287 |
| **Primary education level** |  |  |  |  |  |  |
| Single fall | -0.015 (-0.164, 0.134) |  | **-0.167 (-0.273, -0.061)** |  | -0.063 (-0.159, 0.033) |  |
| Multiple falls | **-0.565 (-0.727, -0.404)** |  | **-0.450 (-0.565, -0.334)** |  | **-0.163 (-0.268, -0.059)** |  |
| Single fall × pre_time | -0.022 (-0.058, 0.014) |  | -0.013 (-0.040, 0.014) |  | -0.016 (-0.038, 0.006) |  |
| Multiple falls × pre_time | **-0.144 (-0.186, -0.101)** |  | **-0.046 (-0.078, -0.014)** |  | -0.021 (-0.047, 0.005) |  |
| Single fall× post_time | 0.025 (-0.013, 0.063) |  | 0.002 (-0.027, 0.031) |  | -0.007 (-0.031, 0.016) |  |
| Multiple falls × post_time | **-0.065 (-0.104, -0.025)** |  | -0.009 (-0.039, 0.022) |  | -0.009 (-0.034, 0.016) |  |
| **Secondary education level** |  |  |  |  |  |  |
| Single fall | -0.056 (-0.135, 0.023) | 0.559 | -0.055 (-0.117, 0.007) | 0.067 | 0.030 (-0.032, 0.092) | 0.204 |
| Multiple falls | **-0.413 (-0.498, -0.327)** | **0.001** | **-0.248 (-0.316, -0.181)** | **<0.001** | **-0.112 (-0.180, -0.045)** | 0.306 |
| Single fall × pre_time | -0.017 (-0.035, 0.002) | 0.758 | 0.002 (-0.013, 0.017) | 0.314 | -0.005 (-0.019, 0.009) | 0.419 |
| Multiple falls × pre_time | **-0.108 (-0.129, -0.087)** | 0.071 | **-0.020 (-0.037, -0.003)** | 0.121 | **-0.018 (-0.034, -0.002)** | 0.842 |
| Single fall× post_time | **-0.026 (-0.045, -0.006)** | **0.008** | -0.003 (-0.019, 0.013) | 0.729 | -0.009 (-0.023, 0.006) | 0.884 |
| Multiple falls × post_time | **-0.092 (-0.112, -0.071)** | 0.182 | -0.004 (-0.021, 0.013) | 0.782 | **-0.018 (-0.035, -0.002)** | 0.518 |
| **Tertiary education level** |  |  |  |  |  |  |
| Single fall | -0.066 (-0.172, 0.040) | 0.613 | **-0.095 (-0.183, -0.007)** | 0.462 | 0.076 (-0.023, 0.176) | 0.264 |
| Multiple falls | **-0.365 (-0.482, -0.247)** | **0.002** | **-0.188 (-0.286, -0.090)** | **<0.001** | **-0.124 (-0.235, -0.013)** | 0.437 |
| Single fall × pre_time | -0.008 (-0.034, 0.019) | 0.550 | -0.003 (-0.025, 0.018) | 0.630 | 0.000 (-0.022, 0.022) | 0.340 |
| Multiple falls × pre_time | **-0.099 (-0.132, -0.066)** | 0.083 | -0.015 (-0.041, 0.011) | 0.145 | **-0.030 (-0.058, -0.003)** | 0.648 |
| Single fall× post_time | 0.012 (-0.016, 0.040) | 0.552 | -0.003 (-0.026, 0.020) | 0.755 | -0.005 (-0.029, 0.019) | 0.984 |
| Multiple falls × post_time | -0.014 (-0.044, 0.016) | 0.061 | 0.013 (-0.012, 0.037) | 0.307 | 0.020 (-0.006, 0.047) | 0.109 |
| **Lower wealth** |  |  |  |  |  |  |
| Single fall | -0.024 (-0.127, 0.078) |  | **-0.107 (-0.181, -0.033)** |  | 0.059 (-0.008, 0.127) |  |
| Multiple falls | **-0.522 (-0.634, -0.411)** |  | **-0.340 (-0.421, -0.259)** |  | **-0.104 (-0.177, -0.030)** |  |
| Single fall × pre_time | -0.022 (-0.046, 0.002) |  | -0.006 (-0.023, 0.012) |  | 0.001 (-0.014, 0.016) |  |
| Multiple falls × pre_time | **-0.126 (-0.154, -0.099)** |  | **-0.035 (-0.056, -0.014)** |  | **-0.018 (-0.036, -0.001)** |  |
| Single fall× post_time | -0.021 (-0.047, 0.004) |  | -0.011 (-0.031, 0.008) |  | **-0.022 (-0.038, -0.005)** |  |
| Multiple falls × post_time | **-0.097 (-0.124, -0.070)** |  | -0.009 (-0.029, 0.012) |  | -0.011 (-0.029, 0.007) |  |
| **Upper wealth** |  |  |  |  |  |  |
| Single fall | **-0.066 (-0.132, 0.000)** | 0.537 | **-0.081 (-0.137, -0.026)** | 0.586 | -0.013 (-0.075, 0.049) | **0.032** |
| Multiple falls | **-0.370 (-0.443, -0.297)** | **0.003** | **-0.250 (-0.312, -0.189)** | **0.042** | **-0.136 (-0.204, -0.067)** | 0.464 |
| Single fall × pre_time | -0.005 (-0.021, 0.012) | 0.231 | -0.006 (-0.020, 0.008) | 0.993 | -0.012 (-0.026, 0.002) | 0.185 |
| Multiple falls × pre_time | **-0.101 (-0.120, -0.081)** | 0.138 | **-0.020 (-0.037, -0.003)** | 0.234 | **-0.021 (-0.038, -0.005)** | 0.785 |
| Single fall× post_time | -0.002 (-0.019, 0.014) | 0.223 | 0.003 (-0.011, 0.017) | 0.243 | 0.004 (-0.010, 0.019) | **0.022** |
| Multiple falls × post_time | **-0.045 (-0.063, -0.026)** | **0.002** | 0.004 (-0.011, 0.020) | 0.335 | -0.001 (-0.017, 0.015) | 0.414 |

Models were adjusted age, sex, cohort, education level, total household wealth, marital status, current smoking status, current alcohol consumption, physical activity, stroke, arthritis, memory-related diseases, eyesight, hearing, and number of other comorbidities;

**Bolded text indicates statistically significant.**

**Table Q.** Subgroup analyses of estimated changes in physical function, depressive symptoms, and cognitive performance before and after hip fracture

|  | **Physical function β (95% CI)** | ***P*-interaction** | **Depressive symptoms β (95% CI)** | ***P*-interaction** | **Cognitive performance β (95% CI)** | ***P*-interaction** |
| --- | --- | --- | --- | --- | --- | --- |
| **Age (< 75 years)** |  |  |  |  |  |  |
| Falls without hip fracture | **-0.215 (-0.260, -0.169)** |  | **-0.202 (-0.241, -0.162)** |  | **-0.042 (-0.082, -0.002)** |  |
| Falls with hip fracture | **-1.364 (-1.614, -1.115)** |  | **-0.445 (-0.664, -0.225)** |  | **-0.283 (-0.509, -0.057)** |  |
| Falls without hip fracture × pre_time | **-0.039 (-0.049, -0.028)** |  | **-0.014 (-0.023, -0.005)** |  | -0.008 (-0.017, 0.000) |  |
| Falls with hip fracture × pre_time | **-0.304 (-0.364, -0.244)** |  | **-0.096 (-0.148, -0.044)** |  | -0.023 (-0.075, 0.030) |  |
| Falls without hip fracture × post_time | **-0.028 (-0.038, -0.017)** |  | 0.000 (-0.009, 0.009) |  | -0.002 (-0.011, 0.007) |  |
| Falls with hip fracture × post_time | 0.031 (-0.031, 0.092) |  | **0.068 (0.010, 0.126)** |  | -0.045 (-0.104, 0.014) |  |
| **Age (≥ 75 years)** |  |  |  |  |  |  |
| Falls without hip fracture | **-0.307 (-0.408, -0.207)** | **0.042** | **-0.240 (-0.305, -0.174)** | 0.337 | -0.021 (-0.088, 0.046) | 0.549 |
| Falls with hip fracture | **-1.275 (-1.617, -0.932)** | 0.888 | **-0.432 (-0.670, -0.195)** | 0.929 | **-0.266 (-0.508, -0.024)** | 0.974 |
| Falls without hip fracture × pre_time | **-0.088 (-0.116, -0.060)** | **<0.001** | **-0.027 (-0.046, -0.009)** | 0.189 | 0.002 (-0.014, 0.018) | 0.250 |
| Falls with hip fracture × pre_time | **-0.317 (-0.412, -0.222)** | 0.839 | -0.033 (-0.100, 0.033) | 0.134 | -0.015 (-0.076, 0.046) | 0.794 |
| Falls without hip fracture × post_time | **-0.061 (-0.088, -0.034)** | **0.006** | -0.014 (-0.032, 0.004) | 0.166 | **-0.019 (-0.035, -0.003)** | 0.075 |
| Falls with hip fracture × post_time | -0.089 (-0.194, 0.016) | **0.030** | **0.096 (0.021, 0.170)** | 0.549 | -0.011 (-0.082, 0.060) | 0.480 |
| **Male** |  |  |  |  |  |  |
| Falls without hip fracture | **-0.285 (-0.346, -0.223)** |  | **-0.216 (-0.261, -0.171)** |  | -0.024 (-0.070, 0.023) |  |
| Falls with hip fracture | **-1.098 (-1.410, -0.786)** |  | **-0.344 (-0.580, -0.108)** |  | **-0.264 (-0.506, -0.022)** |  |
| Falls without hip fracture × pre_time | **-0.059 (-0.074, -0.044)** |  | **-0.019 (-0.029, -0.008)** |  | -0.003 (-0.014, 0.007) |  |
| Falls with hip fracture × pre_time | **-0.237 (-0.321, -0.153)** |  | **-0.075 (-0.137, -0.012)** |  | -0.015 (-0.078, 0.047) |  |
| Falls without hip fracture × post_time | **-0.038 (-0.054, -0.023)** |  | 0.006 (-0.005, 0.017) |  | -0.005 (-0.016, 0.006) |  |
| Falls with hip fracture × post_time | **-0.130 (-0.215, -0.045)** |  | **0.080 (0.011, 0.149)** |  | -0.004 (-0.076, 0.067) |  |
| **Female** |  |  |  |  |  |  |
| Falls without hip fracture | **-0.189 (-0.250, -0.129)** | **0.042** | **-0.216 (-0.264, -0.167)** | 0.998 | -0.031 (-0.078, 0.016) | 0.765 |
| Falls with hip fracture | **-1.393 (-1.646, -1.139)** | 0.084 | **-0.519 (-0.733, -0.304)** | 0.244 | -0.200 (-0.411, 0.010) | 0.773 |
| Falls without hip fracture × pre_time | **-0.044 (-0.058, -0.029)** | 0.134 | **-0.017 (-0.029, -0.006)** | 0.883 | **-0.011 (-0.022, 0.000)** | 0.282 |
| Falls with hip fracture × pre_time | **-0.353 (-0.416, -0.290)** | **0.030** | **-0.070 (-0.124, -0.016)** | 0.911 | -0.036 (-0.087, 0.016) | 0.631 |
| Falls without hip fracture × post_time | **-0.040 (-0.053, -0.026)** | 0.957 | -0.010 (-0.022, 0.001) | **0.046** | -0.009 (-0.020, 0.002) | 0.703 |
| Falls with hip fracture × post_time | 0.035 (-0.033, 0.103) | **0.002** | **0.081 (0.021, 0.142)** | 0.992 | -0.053 (-0.112, 0.006) | 0.298 |
| **Primary education level** |  |  |  |  |  |  |
| Falls without hip fracture | **-0.291 (-0.364, -0.217)** |  | **-0.287 (-0.342, -0.231)** |  | -0.049 (-0.105, 0.007) |  |
| Falls with hip fracture | **-1.307 (-1.594, -1.020)** |  | **-0.519 (-0.747, -0.290)** |  | **-0.235 (-0.466, -0.004)** |  |
| Falls without hip fracture × pre_time | **-0.061 (-0.078, -0.044)** |  | **-0.026 (-0.039, -0.013)** |  | -0.008 (-0.020, 0.004) |  |
| Falls with hip fracture × pre_time | **-0.319 (-0.392, -0.246)** |  | **-0.083 (-0.141, -0.025)** |  | -0.013 (-0.071, 0.044) |  |
| Falls without hip fracture × post_time | **-0.038 (-0.055, -0.021)** |  | 0.003 (-0.010, 0.016) |  | -0.008 (-0.022, 0.006) |  |
| Falls with hip fracture × post_time | -0.007 (-0.082, 0.068) |  | **0.090 (0.027, 0.153)** |  | -0.033 (-0.098, 0.032) |  |
| **Secondary education level** |  |  |  |  |  |  |
| Falls without hip fracture | **-0.206 (-0.270, -0.143)** | **0.032** | **-0.147 (-0.198, -0.096)** | **<0.001** | -0.026 (-0.075, 0.024) | 0.732 |
| Falls with hip fracture | **-1.293 (-1.628, -0.957)** | 0.799 | **-0.388 (-0.660, -0.117)** | 0.470 | -0.240 (-0.504, 0.025) | 0.939 |
| Falls without hip fracture × pre_time | **-0.052 (-0.068, -0.037)** | 0.409 | -0.010 (-0.023, 0.002) | 0.079 | -0.007 (-0.019, 0.005) | 0.926 |
| Falls with hip fracture × pre_time | **-0.337 (-0.429, -0.246)** | 0.798 | -0.042 (-0.118, 0.034) | 0.371 | **-0.084 (-0.154, -0.014)** | 0.103 |
| Falls without hip fracture × post_time | **-0.044 (-0.059, -0.028)** | 0.706 | -0.009 (-0.021, 0.004) | 0.189 | -0.011 (-0.023, 0.001) | 0.677 |
| Falls with hip fracture × post_time | -0.059 (-0.150, 0.031) | 0.412 | 0.073 (-0.004, 0.149) | 0.718 | -0.009 (-0.081, 0.062) | 0.635 |
| **Tertiary education level** |  |  |  |  |  |  |
| Falls without hip fracture | **-0.168 (-0.259, -0.077)** | **0.022** | **-0.172 (-0.247, -0.096)** | **0.033** | 0.010 (-0.070, 0.090) | 0.514 |
| Falls with hip fracture | **-1.081 (-1.559, -0.604)** | 0.352 | -0.164 (-0.574, 0.245) | 0.139 | -0.111 (-0.542, 0.320) | 0.628 |
| Falls without hip fracture × pre_time | **-0.030 (-0.054, -0.007)** | 0.060 | -0.009 (-0.028, 0.009) | 0.195 | -0.013 (-0.032, 0.006) | 0.610 |
| Falls with hip fracture × pre_time | **-0.217 (-0.335, -0.099)** | 0.181 | -0.055 (-0.150, 0.040) | 0.622 | 0.021 (-0.074, 0.117) | 0.633 |
| Falls without hip fracture × post_time | **-0.023 (-0.046, 0.000)** | 0.368 | -0.004 (-0.022, 0.015) | 0.565 | 0.002 (-0.017, 0.021) | 0.469 |
| Falls with hip fracture × post_time | -0.023 (-0.206, 0.159) | 0.844 | 0.033 (-0.116, 0.183) | 0.624 | -0.131 (-0.281, 0.019) | 0.261 |
| **Lower wealth** |  |  |  |  |  |  |
| Falls without hip fracture | **-0.277 (-0.349, -0.206)** |  | **-0.237 (-0.290, -0.184)** |  | 0.010 (-0.039, 0.060) |  |
| Falls with hip fracture | **-1.218 (-1.526, -0.909)** |  | **-0.501 (-0.736, -0.265)** |  | -0.199 (-0.422, 0.025) |  |
| Falls without hip fracture × pre_time | **-0.061 (-0.079, -0.044)** |  | **-0.020 (-0.033, -0.008)** |  | -0.007 (-0.019, 0.004) |  |
| Falls with hip fracture × pre_time | **-0.280 (-0.356, -0.204)** |  | **-0.059 (-0.118, -0.001)** |  | -0.012 (-0.065, 0.041) |  |
| Falls without hip fracture × post_time | **-0.052 (-0.069, -0.035)** |  | -0.003 (-0.016, 0.010) |  | -0.011 (-0.023, 0.001) |  |
| Falls with hip fracture × post_time | -0.082 (-0.165, 0.002) |  | **0.074 (0.004, 0.144)** |  | -0.051 (-0.118, 0.016) |  |
| **Upper wealth** |  |  |  |  |  |  |
| Falls without hip fracture | **-0.192 (-0.242, -0.142)** | **0.017** | **-0.191 (-0.233, -0.148)** | 0.099 | **-0.058 (-0.104, -0.012)** | **0.022** |
| Falls with hip fracture | **-1.447 (-1.687, -1.208)** | 0.408 | **-0.430 (-0.645, -0.215)** | 0.606 | **-0.293 (-0.528, -0.058)** | 0.586 |
| Falls without hip fracture × pre_time | **-0.041 (-0.053, -0.028)** | 0.055 | **-0.017 (-0.027, -0.006)** | 0.631 | -0.008 (-0.018, 0.003) | 0.931 |
| Falls with hip fracture × pre_time | **-0.369 (-0.435, -0.303)** | 0.093 | **-0.091 (-0.149, -0.033)** | 0.466 | -0.054 (-0.115, 0.008) | 0.282 |
| Falls without hip fracture × post_time | **-0.028 (-0.040, -0.016)** | **0.031** | -0.003 (-0.013, 0.007) | 0.921 | -0.003 (-0.013, 0.008) | 0.297 |
| Falls with hip fracture × post_time | 0.047 (-0.018, 0.113) | **0.022** | **0.089 (0.031, 0.148)** | 0.740 | -0.012 (-0.074, 0.050) | 0.396 |

Models were adjusted age, sex, cohort, education level, total household wealth, marital status, current smoking status, current alcohol consumption, physical activity, stroke, arthritis, memory-related diseases, eyesight, hearing, and number of other comorbidities;

**Bolded text indicates statistically significant.**

**Table R.** Sensitivity analysis 1: Including participants with at least one measurement both before and after the fall (matching) time point

|  | **Physical function β (95% CI)** | **Depressive symptoms β (95% CI)** | **Cognitive performance  β (95% CI)** |
| --- | --- | --- | --- |
| **Fall status ^a^** |  |  |  |
| Falls *(n = 4,721)* | **-0.136 (-0.178, -0.094)** | **-0.193 (-0.226, -0.159)** | **0.038 (0.004, 0.071)** |
| Falls × pre_time | **-0.028 (-0.038, -0.017)** | **-0.015 (-0.023, -0.007)** | **-0.009 (-0.017, -0.001)** |
| Falls × post_time | **-0.046 (-0.056, -0.036)** | -0.002 (-0.011, 0.006) | **-0.010 (-0.018, -0.002)** |
| **Injuries from falls ^a^** |  |  |  |
| Falls without injuries *(n = 2,755)* | **-0.113 (-0.162, -0.065)** | **-0.145 (-0.184, -0.106)** | 0.025 (-0.014, 0.064) |
| Falls with injuries *(n = 1,459)* | **-0.192 (-0.253, -0.130)** | **-0.245 (-0.294, -0.196)** | 0.030 (-0.019, 0.079) |
| Falls without injuries × pre_time | **-0.021 (-0.032, -0.009)** | -0.007 (-0.016, 0.002) | -0.006 (-0.015, 0.002) |
| Falls with injuries × pre_time | **-0.042 (-0.057, -0.027)** | **-0.027 (-0.039, -0.015)** | **-0.016 (-0.027, -0.004)** |
| Falls without injuries × post_time | **-0.030 (-0.042, -0.019)** | 0.002 (-0.007, 0.012) | -0.003 (-0.013, 0.006) |
| Falls with injuries × post_time | **-0.035 (-0.049, -0.021)** | -0.004 (-0.016, 0.008) | **-0.015 (-0.027, -0.004)** |
| **Recurrent falls ^b^** |  |  |  |
| Single fall *(n = 1,832)* | -0.018 (-0.078, 0.041) | **-0.084 (-0.131, -0.038)** | 0.036 (-0.010, 0.081) |
| Multiple falls *(n = 1,382)* | **-0.322 (-0.388, -0.257)** | **-0.282 (-0.333, -0.230)** | **-0.082 (-0.133, -0.031)** |
| Single fall × pre_time | -0.005 (-0.019, 0.009) | -0.004 (-0.015, 0.007) | -0.005 (-0.015, 0.005) |
| Multiple falls × pre_time | **-0.077 (-0.093, -0.060)** | **-0.022 (-0.036, -0.009)** | **-0.021 (-0.033, -0.008)** |
| Single fall× post_time | -0.012 (-0.027, 0.002) | -0.004 (-0.016, 0.008) | -0.008 (-0.019, 0.003) |
| Multiple falls× post_time | **-0.070 (-0.086, -0.055)** | -0.002 (-0.015, 0.011) | -0.007 (-0.019, 0.005) |
| **Falls with hip fracture ^c^** |  |  |  |
| Falls without hip fracture *(n = 4,443)* | **-0.140 (-0.182, -0.097)** | **-0.192 (-0.226, -0.157)** | **0.035 (0.001, 0.069)** |
| Falls with hip fracture *(n = 93)* | **-0.661 (-0.880, -0.441)** | **-0.416 (-0.594, -0.238)** | -0.090 (-0.264, 0.083) |
| Falls without hip fracture × pre_time | **-0.027 (-0.037, -0.017)** | **-0.015 (-0.023, -0.007)** | **-0.010 (-0.017, 0.002)** |
| Falls with hip fracture × pre_time | **-0.153 (-0.211, -0.094)** | **-0.057 (-0.104, -0.009)** | -0.038 (-0.081, 0.005) |
| Falls without hip fracture × post_time | **-0.037 (-0.047, -0.027)** | -0.003 (-0.012, 0.005) | **-0.009 (-0.017, -0.001)** |
| Falls with hip fracture × post_time | **-0.104 (-0.161, -0.048)** | **0.061 (0.011, 0.112)** | -0.039 (-0.086, 0.008) |

Models were adjusted age, sex, cohort, education level, total household wealth, marital status, current smoking status, current alcohol consumption, physical activity, stroke, arthritis, memory-related diseases, eyesight, hearing, and number of other comorbidities;

NA: not applicable for the quadratic time term;

**Bolded text indicates statistically significant;**

a. Available for CHARLS, ELSA, and HRS, Ref: No falls (n = 5,495);

b. Available for ELSA, and HRS, Ref: No falls (n = 3,976);

c. Available for CHARLS, ELSA, and HRS, Ref: No falls (n = 5,371).

**Table S.** Sensitivity analysis 2: Including participants with ≥ 3 measurements of physical function, depressive symptoms, and cognitive performance

|  | **Physical function β (95% CI)** | **Depressive symptoms β (95% CI)** | **Cognitive performance  β (95% CI)** |
| --- | --- | --- | --- |
| **Fall status ^a^** |  |  |  |
| Falls *(n = 4,366)* | **-0.188 (-0.229, -0.146)** | **-0.203 (-0.238, -0.167)** | -0.017 (-0.051, 0.018) |
| Falls × pre_time | **-0.039 (-0.049, -0.029)** | **-0.018 (-0.026, -0.009)** | -0.005 (-0.013, 0.002) |
| Falls × post_time | **-0.039 (-0.048, -0.030)** | -0.003 (-0.011, 0.005) | **-0.009 (-0.017, -0.001)** |
| **Injuries from falls ^a^** |  |  |  |
| Falls without injuries *(n = 2,550)* | **-0.167 (-0.214, -0.120)** | **-0.160 (-0.200, -0.119)** | -0.027 (-0.067, 0.013) |
| Falls with injuries *(n = 1,335)* | **-0.238 (-0.297, -0.179)** | **-0.245 (-0.296, -0.194)** | -0.025 (-0.075, 0.025) |
| Falls without injuries × pre_time | **-0.033 (-0.043, -0.022)** | **-0.009 (-0.019, 0.000)** | -0.003 (-0.012, 0.006) |
| Falls with injuries × pre_time | **-0.053 (-0.066, -0.039)** | **-0.029 (-0.040, -0.017)** | -0.011 (-0.022, 0.000) |
| Falls without injuries × post_time | **-0.025 (-0.036, -0.015)** | 0.001 (-0.009, 0.011) | -0.001 (-0.010, 0.008) |
| Falls with injuries × post_time | **-0.029 (-0.042, -0.016)** | -0.004 (-0.015, 0.008) | **-0.017 (-0.028, -0.005)** |
| **Recurrent falls ^b^** |  |  |  |
| Single fall *(n = 1,706)* | **-0.056 (-0.112, 0.000)** | **-0.086 (-0.133, -0.039)** | 0.005 (-0.042, 0.052) |
| Multiple falls *(n = 1,231)* | **-0.358 (-0.422, -0.295)** | **-0.274 (-0.328, -0.221)** | **-0.110 (-0.163, -0.057)** |
| Single fall × pre_time | -0.012 (-0.025, 0.001) | -0.004 (-0.015, 0.007) | -0.005 (-0.016, 0.005) |
| Multiple falls × pre_time | **-0.080 (-0.095, 0.065)** | **-0.022 (-0.035, -0.008)** | **-0.020 (-0.032, -0.007)** |
| Single fall× post_time | **-0.016 (-0.029, -0.003)** | -0.005 (-0.016, 0.007) | -0.006 (-0.017, 0.004) |
| Multiple falls× post_time | **-0.061 (-0.076, -0.047)** | -0.005 (-0.017, 0.008) | -0.006 (-0.018, 0.006) |
| **Falls with hip fracture ^c^** |  |  |  |
| Falls without hip fracture *(n = 4,120)* | **-0.186 (-0.227, -0.145)** | **-0.203 (-0.239, -0.167)** | -0.018 (-0.035, 0.017) |
| Falls with hip fracture *(n = 85)* | **-0.715 (-0.923, -0.506)** | **-0.363 (-0.546, -0.180)** | -0.161 (-0.339, 0.017) |
| Falls without hip fracture × pre_time | **-0.038 (-0.047, -0.028)** | **-0.017 (-0.026, -0.009)** | -0.006 (-0.014, 0.001) |
| Falls with hip fracture × pre_time | **-0.168 (-0.218, -0.117)** | **-0.067 (-0.112, -0.022)** | -0.032 (-0.074, 0.010) |
| Falls without hip fracture × post_time | **-0.034 (-0.043, -0.024)** | -0.004 (-0.013, 0.004) | -0.008 (-0.016, 0.000) |
| Falls with hip fracture × post_time | **-0.093 (-0.146, -0.041)** | **0.066 (0.016, 0.115)** | -0.041 (-0.087, 0.005) |

Models were adjusted age, sex, cohort, education level, total household wealth, marital status, current smoking status, current alcohol consumption, physical activity, stroke, arthritis, memory-related diseases, eyesight, hearing, and number of other comorbidities;

NA: not applicable for the quadratic time term;

**Bolded text indicates statistically significant;**

a. Available for CHARLS, ELSA, and HRS, Ref: No falls (n = 4,309);

b. Available for ELSA, and HRS, Ref: No falls (n = 3,379);

c. Available for CHARLS, ELSA, and HRS, Ref: No falls (n = 4,227).

**Table T.** Sensitivity analysis 3: Conducting spline-based mixed-effects models with natural cubic splines of 2 degrees of freedom

|  | **Physical function β (95% CI)** | **Depressive symptoms β (95% CI)** | **Cognitive performance  β (95% CI)** |
| --- | --- | --- | --- |
| **Fall status ^a^** |  |  |  |
| Falls *(n = 5,495)* | **-0.293 (-0.342, -0.244)** | **-0.234 (-0.271, -0.197)** | **-0.044 (-0.080, -0.007)** |
| Falls × pre_spline1 | -0.079 (-0.342, -0.185) | -0.009 (-0.205, 0.186) | -0.013 (-0.197, 0.171) |
| Falls × pre_spline2 | **-0.362 (-0.428, -0.295)** | **-0.124 (-0.174, -0.073)** | **-0.056 (-0.103, -0.009)** |
| Falls × post_spline1 | **-0.286 (-0.388, -0.184)** | 0.254 (-0.052, 0.103) | -0.027 (-0.100, 0.045) |
| Falls × post_spline2 | **-0.250 (-0.355, -0.144)** | -0.020 (-0.103, 0.062) | **-0.108 (-0.192, -0.024)** |
| **Injuries from falls ^a^** |  |  |  |
| Falls without injuries *(n = 3,131)* | **-0.220 (-0.275, -0.134)** | **-0.188 (-0.231, -0.145)** | **-0.049 (-0.092, -0.007)** |
| Falls with injuries *(n = 1,795)* | **-0.452 (-0.520, -0.385)** | **-0.291 (-0.343, -0.239)** | **-0.063 (-0.115, -0.011)** |
| Falls without injuries × pre_spline1 | -0.178 (-0.467, 0.112) | 0.022 (-0.196, 0.241) | 0.035 (-0.173, 0.243) |
| Falls with injuries × pre_spline1 | 0.020 (-0.338, 0.378) | -0.044 (-0.316, 0.227) | -0.198 (-0.459, 0.064) |
| Falls without injuries × pre_spline2 | **-0.255 (-0.329, -0.180)** | **-0.081 (-0.137, -0.024)** | -0.045 (-0.099, 0.008) |
| Falls with injuries × pre_spline2 | **-0.577 (-0.668, -0.486)** | **-0.193 (-0.263, -0.122)** | **-0.086 (-0.154, -0.019)** |
| Falls without injuries × post_spline1 | **-0.227 (-0.343, -0.110)** | 0.070 (-0.020, 0.159) | 0.039 (-0.046, 0.123) |
| Falls with injuries × post_spline1 | -0.054 (-0.192, 0.085) | 0.024 (-0.085, 0.132) | -0.094 (-0.198, 0.010) |
| Falls without injuries × post_spline2 | -0.091 (-0.218, 0.034) | -0.009 (-0.109, 0.091) | -0.056 (-0.156, 0.044) |
| Falls with injuries × post_spline2 | **-0.230 (-0.371, -0.089)** | -0.024 (-0.139, 0.090) | **-0.146 (-0.268, -0.024)** |
| **Recurrent falls ^b^** |  |  |  |
| Single fall *(n = 1,901)* | -0.059 (-0.126, 0.008) | **-0.102 (-0.153, -0.050)** | -0.003 (-0.052, 0.048) |
| Multiple falls *(n = 1,516)* | **-0.516 (-0.589, -0.443)** | **-0.326 (-0.383, -0.270)** | **-0.143 (-0.198, -0.088)** |
| Single fall × pre_spline1 | -0.006 (-0.381, 0.369) | 0.070 (-0.223, 0.363) | 0.118 (-0.149, -0.385) |
| Multiple falls × pre_spline1 | 0.252 (-0.204, 0.709) | 0.023 (-0.335, 0.381) | 0.013 (-0.312, 0.337) |
| Single fall × pre_spline2 | -0.088 (-0.177, 0.001) | -0.035 (-0.105, 0.035) | -0.055 (-0.119, 0.009) |
| Multiple falls × pre_spline2 | **-0.646 (-0.750, -0.542)** | **-0.173 (-0.256, -0.091)** | **-0.128 (-0.203, -0.053)** |
| Single fall × post_spline1 | -0.056 (-0.193, 0.081) | -0.009 (-0.117, 0.100) | -0.002 (-0.101, 0.097) |
| Multiple falls ×post _spline1 | **-0.411 (-0.558, -0.264)** | 0.062 (-0.056, 0.180) | 0.002 (-0.106, 0.109) |
| Single fall × post_spline2 | -0.076 (-0.239, 0.088) | -0.030 (-0.160, 0.101) | -0.087 (-0.207, 0.033) |
| Multiple falls × post_spline2 | **-0.233 (-0.408, -0.057)** | -0.070 (-0.212, 0.072) | -0.065 (-0.198, 0.068) |
| **Falls with hip fracture ^c^** |  |  |  |
| Falls without hip fracture *(n = 5,120)* | **-0.270 (-0.318, 0.221)** | **-0.232 (-0.370, -0.194)** | **-0.041 (-0.078, -0.003)** |
| Falls with hip fracture *(n = 135)* | **-1.522 (-1.736, -1.308)** | **-0.450 (-0.624, -0.277)** | **-0.241 (-0.413, -0.069)** |
| Falls without hip fracture × pre_spline1 | -0.088 (-0.350, -0.173) | 0.015 (-0.184, 0.214) | -0.040 (-0.228, 0.147) |
| Falls with hip fracture × pre_spline1 | 0.088 (-0.786, 2.537) | -0.710 (-2.003, -0.574) | -0.028 (-1.328, 1.272) |
| Falls without hip fracture × pre_spline2 | **-0.328 (-0.394, -0.261)** | **-0.120 (-0.171, -0.069)** | **-0.059 (-0.108, -0.011)** |
| Falls with hip fracture × pre_spline2 | **-1.747 (-2.060, -1.434)** | **-0.407 (-0.658, -0.156)** | -0.148 (-0.375, 0.098) |
| Falls without hip fracture × post_spline1 | **-0.234 (-0.334, -0.133)** | 0.016 (-0.063, 0.094) | -0.021 (-0.095, 0.053) |
| Falls with hip fracture × post_spline1 | 0.328 (-0.148, 0.805) | **0.612 (0.214, 1.010)** | -0.227 (-0.614, 0.160) |
| Falls without hip fracture × post_spline2 | **-0.207 (-0.312, -0.102)** | -0.020 (-0.103, 0.064) | **-0.099 (-0.184, -0.014)** |
| Falls with hip fracture × post_spline2 | -0.322 (-0.905, 0.261) | **0.064 (0.110, 1.163)** | -0.190 (-0.731, 0.351) |

Models were adjusted age, sex, cohort, education level, total household wealth, marital status, current smoking status, current alcohol consumption, physical activity, stroke, arthritis, memory-related diseases, eyesight, hearing, and number of other comorbidities;

**Bolded text indicates statistically significant;**

**Note:** pre/post_spline represents a centered natural cubic spline basis term for time before/after matching wave; Spline terms were used to flexibly model potential non-linearity in functional trajectories; individual spline coefficients are not directly interpreted. Pre_fall internal knot: time = -2.25; and post_fall internal knot: time = 2.92;

a. Available for CHARLS, ELSA, and HRS, Ref: No falls (n = 5,495);

b. Available for ELSA, and HRS, Ref: No falls (n = 3,976);

c. Available for CHARLS, ELSA, and HRS, Ref: No falls (n = 5,371).

**Table U.** Sensitivity analysis 4: Redefining ADL using five core items common to all cohorts

|  | **Physical function β (95% CI)** |
| --- | --- |
| **Fall status ^a^** |  |
| Falls *(n = 5,495)* | **-0.283 (-0.328, -0.238)** |
| Falls × pre-time | **-0.111 (-0.137, -0.085)** |
| Falls × pre-time^2^ | **-0.009 (-0.013, -0.005)** |
| Falls × post-time | **-0.034 (-0.045, -0.023)** |
| **Injuries from falls ^a^** |  |
| Falls without injuries *(n = 3,131)* | **-0.222 (-0.274, -0.170)** |
| Falls with injuries *(n = 1,795)* | **-0.418 (-0.481, -0.356)** |
| Falls without injuries × pre-time | **-0.072 (-0.101, -0.043)** |
| Falls with injuries × pre-time | **-0.184 (-0.219, -0.148)** |
| Falls without injuries × pre-time^2^ | **-0.005 (-0.009, -0.001)** |
| Falls with injuries × pre-time^2^ | **-0.016 (-0.022, -0.011)** |
| Falls without injuries × post-time | **-0.022 (-0.035, -0.010)** |
| Falls with injuries × post-time | **-0.017 (-0.031, -0.002)** |
| **Recurrent falls ^b^** |  |
| Single fall *(n = 1,901)* | **-0.064 (-0.126, -0.002)** |
| Multiple falls *(n = 1,516)* | **-0.517 (-0.585, -0.449)** |
| Single fall × pre-time | -0.028 (-0.064, 0.007) |
| Multiple falls × pre-time | **-0.253 (-0.293, -0.214)** |
| Single fall × pre-time^2^ | -0.002 (-0.007, 0.003) |
| Multiple falls × pre-time^2^ | **-0.025 (-0.031, 0.019)** |
| Single fall × post-time | -0.007 (-0.023, 0.008) |
| Multiple falls × post-time | **-0.040 (-0.057, -0.023)** |
| **Falls with hip fracture ^c^** |  |
| Falls without hip fracture *(n = 5,120)* | **-0.264 (-0.309, -0.219)** |
| Falls with hip fracture *(n = 135)* | **-1.358 (-1.559, -1.156)** |
| Falls without hip fracture × pre-time | **-0.102 (-0.127, -0.076)** |
| Falls with hip fracture × pre-time | **-0.625 (-0.759, -0.491)** |
| Falls without hip fracture × pre-time^2^ | **-0.008 (-0.012, -0.004)** |
| Falls with hip fracture × pre-time^2^ | **-0.063 (-0.086, -0.039)** |
| Falls without hip fracture × post-time | **-0.027 (-0.038, -0.016)** |
| Falls with hip fracture × post-time | 0.016 (-0.039, 0.071) |

Models were adjusted age, sex, cohort, education level, total household wealth, marital status, current smoking status, current alcohol consumption, physical activity, stroke, arthritis, memory-related diseases, eyesight, hearing, and number of other comorbidities;

**Bolded text indicates statistically significant;**

a. Available for CHARLS, ELSA, and HRS, Ref: No falls (n = 5,495);

b. Available for ELSA, and HRS, Ref: No falls (n = 3,976);

c. Available for CHARLS, ELSA, and HRS, Ref: No falls (n = 5,371).

**Table U.** Sensitivity analysis 5: Imputing missing covariates using multiple imputation

|  | **Physical function β (95% CI)** | **Depressive symptoms β (95% CI)** | **Cognitive performance  β (95% CI)** |
| --- | --- | --- | --- |
| **Fall status ^a^** |  |  |  |
| Falls *(n = 5,495)* | **-0.256 (-0.301, -0.211)** | **-0.212 (-0.246, -0.179)** | -0.029 (-0.062, 0.004) |
| Falls × pre_time | **-0.108 (-0.133, -0.082)** | **-0.018 (-0.026, -0.010)** | -0.006 (-0.014, 0.001) |
| Falls × pre_time^2^ | **-0.010 (-0.014, -0.006)** | NA | NA |
| Falls × post_time | **-0.039 (-0.050, -0.028)** | -0.002 (-0.011, 0.006) | -0.008 (-0.015, 0.000) |
| **Injuries from falls ^a^** |  |  |  |
| Falls without injuries *(n = 3,131)* | **-0.200 (-0.251, -0.149)** | **-0.167 (-0.205, -0.128)** | -0.034 (-0.072, 0.005) |
| Falls with injuries *(n = 1,795)* | **-0.394 (-0.456, -0.332)** | **-0.259 (-0.306, -0.212)** | **-0.056 (-0.103, -0.008)** |
| Falls without injuries × pre_time | **-0.074 (-0.102, -0.046)** | **-0.010 (-0.019, -0.001)** | -0.004 (-0.012, 0.005) |
| Falls with injuries × pre_time | **-0.178 (-0.213, -0.143)** | **-0.028 (-0.039, -0.016)** | **-0.012 (-0.023, -0.002)** |
| Falls without injuries × pre_time^2^ | **-0.006 (-0.010, -0.002)** | NA | NA |
| Falls with injuries × pre_time^2^ | **-0.017 (-0.022, -0.012)** | NA | NA |
| Falls without injuries × post_time | **-0.026 (-0.039, -0.014)** | 0.002 (-0.008, 0.012) | 0.001 (-0.010, 0.009) |
| Falls with injuries × post_time | **-0.017 (-0.032, -0.002)** | -0.002 (-0.014, 0.010) | **-0.013 (-0.025, -0.002)** |
| **Recurrent falls ^b^** |  |  |  |
| Single fall *(n = 1,901)* | -0.052 (-0.115, 0.010) | **-0.090 (-0.136, -0.044)** | 0.020 (-0.026, 0.065) |
| Multiple falls *(n = 1,516)* | **-0.510 (-0.579, -0.442)** | **-0.297 (-0.348, -0.247)** | **-0.122 (-0.172, -0.072)** |
| Single fall × pre_time | -0.026 (-0.061, 0.010) | -0.005 (-0.016, 0.007) | -0.006 (-0.016, 0.004) |
| Multiple falls × pre_time | **-0.250 (-0.290, -0.210)** | **-0.026 (-0.040, -0.013)** | **-0.020 (-0.032, -0.008)** |
| Single fall × pre_time^2^ | -0.002 (-0.008, 0.003) | NA | NA |
| Multiple falls × pre_time^2^ | **-0.025 (-0.031, -0.018)** | NA | NA |
| Single fall× post_time | -0.010 (-0.026, 0.006) | -0.004 (-0.016, 0.007) | -0.006 (-0.017, 0.005) |
| Multiple falls× post_time | **-0.047 (-0.064, -0.031)** | -0.001 (-0.014, 0.012) | -0.005 (-0.017, 0.007) |
| **Falls with hip fracture ^c^** |  |  |  |
| Falls without hip fracture *(n = 5,120)* | **-0.237 (-0.281, -0.192)** | **-0.211 (-0.245, -0.177)** | -0.028 (-0.061, 0.006) |
| Falls with hip fracture *(n = 135)* | **-1.357 (-1.561, -1.152)** | **-0.465 (-0.627, -0.303)** | **-0.214 (-0.375, -0.052)** |
| Falls without hip fracture × pre_time | **-0.096 (-0.121, -0.070)** | **-0.018 (-0.026, -0.010)** | -0.007 (-0.014, 0.001) |
| Falls with hip fracture × pre_time | **-0.614 (-0.751, -0.477)** | **-0.074 (-0.116, -0.032)** | -0.026 (-0.065, 0.014) |
| Falls without hip fracture × pre_time^2^ | **-0.009 (-0.012, -0.005)** | NA | NA |
| Falls with hip fracture × pre_time^2^ | **-0.063 (-0.088, -0.039)** | NA | NA |
| Falls without hip fracture × post_time | **-0.032 (-0.043, -0.021)** | -0.003 (-0.011, -0.006) | -0.007 (-0.015, 0.001) |
| Falls with hip fracture × post_time | -0.043 (-0.100, 0.015) | **0.076 (0.028, -0.123)** | -0.032 (-0.078, 0.015) |

Models were adjusted age, sex, cohort, education level, total household wealth, marital status, current smoking status, current alcohol consumption, physical activity, stroke, arthritis, memory-related diseases, eyesight, hearing, and number of other comorbidities;

NA: not applicable for the quadratic time term;

**Bolded text indicates statistically significant;**

a. Available for CHARLS, ELSA, and HRS, Ref: No falls (n = 5,495);

b. Available for ELSA, and HRS, Ref: No falls (n = 3,976);

c. Available for CHARLS, ELSA, and HRS, Ref: No falls (n = 5,371).

**Table W.** Baseline characteristics of participants stratified by whether included in the analysis of joint trajectories

|  | **Total (n = 10,990)** | **Not included (n = 2,315)** | **Included (n = 8,675)** | ***P*** |
| --- | --- | --- | --- | --- |
| **Cohort study** |  |  |  | <0.001 |
| CHARLS | 3038 (27.6) | 1196 (51.7) | 1842 (21.2) |  |
| ELSA | 3540 (32.2) | 372 (16.1) | 3168 (36.5) |  |
| HRS | 4412 (40.1) | 747 (32.3) | 3665 (42.2) |  |
| **Age, mean (SD)** | 70.98 (7.22) | 72.93 (8.46) | 70.45 (6.75) | <0.001 |
| **Sex, n (%)** |  |  |  | <0.001 |
| Male | 5010 (45.6) | 935 (40.4) | 4075 (47.0) |  |
| Female | 5980 (54.4) | 1380 (59.6) | 4600 (53.0) |  |
| **Education level, n (%)** |  |  |  | <0.001 |
| Primary | 4802 (43.7) | 1506 (65.1) | 3296 (38.0) |  |
| Secondary | 4366 (39.7) | 600 (25.9) | 3766 (43.4) |  |
| Tertiary | 1525 (13.9) | 175 (7.6) | 1350 (15.6) |  |
| Unknown | 297 (2.7) | 34 (1.5) | 263 (3.0) |  |
| **Total household wealth, n (%)** |  |  |  | <0.001 |
| Q1 (lowest) | 2722 (24.8) | 728 (31.4) | 1994 (23.0) |  |
| Q2 | 2721 (24.8) | 587 (25.4) | 2134 (24.6) |  |
| Q3 | 2721 (24.8) | 534 (23.1) | 2187 (25.2) |  |
| Q4 (highest) | 2720 (24.7) | 440 (19.0) | 2280 (26.3) |  |
| Unknown | 106 (1.0) | 26 (1.1) | 80 (0.9) |  |
| **Marital status, n (%)** |  |  |  | <0.001 |
| Married or partnered | 7683 (69.9) | 1514 (65.4) | 6169 (71.1) |  |
| Widowed | 2217 (20.2) | 645 (27.9) | 1572 (18.1) |  |
| Separated or divorced or single | 1088 (9.9) | 155 (6.7) | 933 (10.8) |  |
| Unknown | 2 (0.0) | 1 (0.0) | 1 (0.0) |  |
| **Current smoking status, n (%)** |  |  |  | <0.001 |
| No | 9352 (85.1) | 1885 (81.4) | 7467 (86.1) |  |
| Yes | 1608 (14.6) | 424 (18.3) | 1184 (13.6) |  |
| Unknown | 30 (0.3) | 6 (0.3) | 24 (0.3) |  |
| **Alcohol consumption, n (%)** |  |  |  | <0.001 |
| Less than weekly drinking | 6403 (58.3) | 1702 (73.5) | 4701 (54.2) |  |
| Weekly drinking or more | 4176 (38.0) | 500 (21.6) | 3676 (42.4) |  |
| Unknown | 411 (3.7) | 113 (4.9) | 298 (3.4) |  |
| **Physical activity, n (%)** |  |  |  | <0.001 |
| Physical inactive | 1848 (16.8) | 637 (27.5) | 1211 (14.0) |  |
| Physical active | 7360 (67.0) | 989 (42.7) | 6371 (73.4) |  |
| Unknown | 1782 (16.2) | 689 (29.8) | 1093 (12.6) |  |
| **Eyesight, n (%)** |  |  |  | <0.001 |
| Good or better | 7259 (66.1) | 1045 (45.1) | 6214 (71.6) |  |
| Fair or worse | 3715 (33.8) | 1263 (54.6) | 2452 (28.3) |  |
| Unknown | 16 (0.1) | 7 (0.3) | 9 (0.1) |  |
| **Hearing, n (%)** |  |  |  | <0.001 |
| Good or better | 7394 (67.3) | 1262 (54.5) | 6132 (70.7) |  |
| Fair or worse | 3590 (32.7) | 1052 (45.4) | 2538 (29.3) |  |
| Unknown | 6 (0.1) | 1 (0.0) | 5 (0.1) |  |
| **Stroke, n (%)** |  |  |  | <0.001 |
| No | 10406 (94.7) | 2126 (91.8) | 8280 (95.4) |  |
| Yes | 579 (5.3) | 186 (8.0) | 393 (4.5) |  |
| Unknown | 5 (0.0) | 3 (0.1) | 2 (0.0) |  |
| **Arthritis, n (%)** |  |  |  |  |
| No | 5660 (51.5) | 1171 (50.6) | 4489 (51.7) | 0.001 |
| Yes | 5324 (48.4) | 1139 (49.2) | 4185 (48.2) |  |
| Unknown | 6 (0.1) | 5 (0.2) | 1 (0.0) |  |
| **Memory-related diseases, n (%)** |  |  |  | <0.001 |
| No | 10824 (98.5) | 2228 (96.2) | 8596 (99.1) |  |
| Yes | 160 (1.5) | 85 (3.7) | 75 (0.9) |  |
| Unknown | 6 (0.1) | 2 (0.1) | 4 (0.0) |  |
| **Number of comorbidities, n (%)** |  |  |  | <0.001 |
| 0 | 3719 (33.8) | 806 (34.8) | 2913 (33.6) |  |
| 1 | 4017 (36.6) | 807 (34.9) | 3210 (37.0) |  |
| ≥ 2 | 3207 (29.2) | 679 (29.3) | 2528 (29.1) |  |
| Unknown | 47 (0.4) | 23 (1.0) | 24 (0.3) |  |

*P* values were calculated by analysis of variance or chi-square test when appropriate;

Abbreviations: CHARLS, China Health and Retirement Longitudinal Study; ELSA, English Longitudinal Study on Ageing; HRS, US Health and Retirement Study.

**Table X.** Joint trajectory models’ results of model fitting process

| **Number of classes** | **Polynomial degree** | **BIC** | **Group membership (%)** | | | | | **Ave PP (%)** | | | | |
| --- | --- | --- | --- | --- | --- | --- | --- | --- | --- | --- | --- | --- |
| 2 | (1 1), (1 1), (1 1) | -158811.47 | 8.83 | 91.17 |  |  |  | 96.65 | 99.62 |  |  |  |
| 2 | (2 2), (2 2), (2 2) | -158809.62 | 8.90 | 91.10 |  |  |  | 96.81 | 99.65 |  |  |  |
| 2 | (3 3), (3 3), (3 3) | -158806.53 | 8.93 | 91.07 |  |  |  | 96.57 | 99.64 |  |  |  |
| 3 | (1 1 1), (1 1 1), (1 1 1) | -153459.08 | 6.79 | 31.80 | 61.41 |  |  | 96.79 | 91.52 | 95.08 |  |  |
| 3 | (2 2 2), (2 2 2), (2 2 2) | -153461.38 | 6.89 | 31.80 | 61.30 |  |  | 96.70 | 91.37 | 95.17 |  |  |
| 3 | (3 3 3), (3 3 3), (3 3 3) | -153565.73 | 6.84 | 31.57 | 61.59 |  |  | 96.69 | 91.54 | 95.08 |  |  |
| 4 | (1 1 1 1), (1 1 1 1), (1 1 1 1) | -149679.38 | 5.48 | 11.09 | 27.10 | 56.33 |  | 96.77 | 92.22 | 89.93 | 94.64 |  |
| **4** | **(2 2 2 2), (2 2 2 2), (2 2 2 2)** | **-149677.36** | **5.56** | **11.09** | **27.00** | **56.36** |  | **96.83** | **92.14** | **89.98** | **94.60** |  |
| 4 | (3 3 3 3), (3 3 3 3), (3 3 3 3) | -149694.53 | 5.49 | 11.08 | 27.00 | 56.44 |  | 96.81 | 92.36 | 89.96 | 94.56 |  |
| 5 | (1 1 1 1 1), (1 1 1 1 1), (1 1 1 1 1) | -148134.75 | 5.16 | 10.55 | 10.54 | 40.21 | 33.54 | 97.11 | 91.62 | 89.51 | 87.51 | 89.90 |
| 5 | (2 2 2 2 2), (2 2 2 2 2), (2 2 2 2 2) | -147205.81 | 2.21 | 10.26 | 6.95 | 25.72 | 54.86 | 98.13 | 92.13 | 93.66 | 89.73 | 94.15 |
| 5 | (3 3 3 3 3), (3 3 3 3 3), (3 3 3 3 3) | -147230.91 | 2.54 | 10.48 | 6.25 | 25.74 | 55.00 | 87.04 | 92.37 | 93.45 | 89.71 | 94.13 |

Abbreviations: BIC, Bayesian information criteria; Ave PP, average posterior probability.

**Table Y.** Characteristics of the study population included in the analysis of joint trajectories of physical function, depressive symptoms, and cognitive performance by identified trajectories

|  | **Total**  **(n = 8675)** | **Identified joint trajectories** | | | | ***P*** |
| --- | --- | --- | --- | --- | --- | --- |
|  |  | **Favorable trajectories of physical function, depressive symptoms, and cognitive performance (n = 4889)** | **Favorable trajectories of physical function and depressive symptoms, combined with low and declining cognitive performance  (n = 2342)** | **Favorable trajectory of physical function, but poor and deteriorating depressive symptoms and cognitive performance  (n = 962)** | **Rapidly declining physical function, combined with poor and worsening depressive symptoms and cognitive performance (n = 482)** |  |
| **Cohort, n (%)** |  |  |  |  |  | <0.001 |
| CHARLS | 1842 (21.2) | 1061 (21.7) | 464 (19.8) | 230 (23.9) | 87 (18.0) |  |
| ELSA | 3168 (36.5) | 1770 (36.2) | 930 (39.7) | 308 (32.0) | 160 (33.2) |  |
| HRS | 3665 (42.2) | 2058 (42.1) | 948 (40.5) | 424 (44.1) | 235 (48.8) |  |
| **Age at baseline, mean (SD)** | 70.5 (6.8) | 69.1 (6.1) | 72.5 (7.0) | 70.8 (7.1) | 73.4 (7.7) | <0.001 |
| **Sex** |  |  |  |  |  |  |
| Male | 4075 (47.0) | 2306 (47.2) | 1226 (52.3) | 333 (34.6) | 210 (43.6) |  |
| Female | 4600 (53.0) | 2583 (52.8) | 1116 (47.7) | 629 (65.4) | 272 (56.4) |  |
| **Education level, n (%)** |  |  |  |  |  | <0.001 |
| Primary | 3296 (38.0) | 1375 (28.1) | 1218 (52.0) | 460 (47.8) | 243 (50.4) |  |
| Secondary | 3766 (43.4) | 2310 (47.2) | 881 (37.6) | 381 (39.6) | 194 (40.2) |  |
| Tertiary | 1350 (15.6) | 1066 (21.8) | 156 (6.7) | 92 (9.6) | 36 (7.5) |  |
| Unknown | 263 (3.0) | 138 (2.8) | 87 (3.7) | 29 (3.0) | 9 (1.9) |  |
| **Total household wealth, n (%)** |  |  |  |  |  | <0.001 |
| Q1 (lowest) | 1994 (23.0) | 752 (15.4) | 702 (30.0) | 340 (35.3) | 200 (41.5) |  |
| Q2 | 2134 (24.6) | 1057 (21.6) | 677 (28.9) | 276 (28.7) | 124 (25.7) |  |
| Q3 | 2187 (25.2) | 1367 (28.0) | 539 (23.0) | 189 (19.6) | 92 (19.1) |  |
| Q4 (highest) | 2280 (26.3) | 1656 (33.9) | 414 (17.7) | 147 (15.3) | 63 (13.1) |  |
| Unknown | 80 (0.9) | 57 (1.2) | 10 (0.4) | 10 (1.0) | 3 (0.6) |  |
| **Marital status, n (%)** |  |  |  |  |  | <0.001 |
| Married or partnered | 6169 (71.1) | 3773 (77.2) | 1546 (66.0) | 572 (59.5) | 278 (57.7) |  |
| Widowed | 1572 (18.1) | 665 (13.6) | 499 (21.3) | 266 (27.7) | 142 (29.5) |  |
| Separated or divorced or single | 933 (10.8) | 450 (9.2) | 297 (12.7) | 124 (12.9) | 62 (12.9) |  |
| Unknown | 1 (0.0) | 1 (0.0) | 0 (0.0) | 0 (0.0) | 0 (0.0) |  |
| **Current smoking status, n (%)** |  |  |  |  |  | 0.001 |
| No | 7467 (86.1) | 4258 (87.1) | 2009 (85.8) | 790 (82.1) | 410 (85.1) |  |
| Yes | 1184 (13.6) | 619 (12.7) | 328 (14.0) | 169 (17.6) | 68 (14.1) |  |
| Unknown | 24 (0.3) | 12 (0.2) | 5 (0.2) | 3 (0.3) | 4 (0.8) |  |
| **Alcohol consumption, n (%)** |  |  |  |  |  | <0.001 |
| Less than weekly drinking | 4701 (54.2) | 2418 (49.5) | 1341 (57.3) | 615 (63.9) | 327 (67.8) |  |
| Weekly drinking or more | 3676 (42.4) | 2336 (47.8) | 895 (38.2) | 316 (32.8) | 129 (26.8) |  |
| Unknown | 298 (3.4) | 135 (2.8) | 106 (4.5) | 31 (3.2) | 26 (5.4) |  |
| **Physical activity, n (%)** |  |  |  |  |  | <0.001 |
| Physical inactive | 1211 (14.0) | 481 (9.8) | 345 (14.7) | 195 (20.3) | 190 (39.4) |  |
| Physical active | 6371 (73.4) | 3790 (77.5) | 1720 (73.4) | 620 (64.4) | 241 (50.0) |  |
| Unknown | 1093 (12.6) | 618 (12.6) | 277 (11.8) | 147 (15.3) | 51 (10.6) |  |
| **Eyesight, n (%)** |  |  |  |  |  | <0.001 |
| Good or better | 6214 (71.6) | 3775 (77.2) | 1614 (68.9) | 560 (58.2) | 265 (55.0) |  |
| Fair or worse | 2452 (28.3) | 1112 (22.7) | 723 (30.9) | 401 (41.7) | 216 (44.8) |  |
| Unknown | 9 (0.1) | 2 (0.0) | 5 (0.2) | 1 (0.1) | 1 (0.2) |  |
| **Hearing, n (%)** |  |  |  |  |  | <0.001 |
| Good or better | 6132 (70.7) | 3712 (75.9) | 1557 (66.5) | 585 (60.8) | 278 (57.7) |  |
| Fair or worse | 2538 (29.3) | 1174 (24.0) | 785 (33.5) | 375 (39.0) | 204 (42.3) |  |
| Unknown | 5 (0.1) | 3 (0.1) | 0 (0.0) | 2 (0.2) | 0 (0.0) |  |
| **Stroke, n (%)** |  |  |  |  |  | <0.001 |
| No | 8280 (95.4) | 4727 (96.7) | 2214 (94.5) | 907 (94.3) | 432 (89.6) |  |
| Yes | 393 (4.5) | 160 (3.3) | 128 (5.5) | 55 (5.7) | 50 (10.4) |  |
| Unknown | 2 (0.0) | 2 (0.0) | 0 (0.0) | 0 (0.0) | 0 (0.0) |  |
| **Arthritis, n (%)** |  |  |  |  |  | <0.001 |
| No | 4489 (51.7) | 2709 (55.4) | 1280 (54.7) | 360 (37.4) | 140 (29.0) |  |
| Yes | 4185 (48.2) | 2180 (44.6) | 1062 (45.3) | 601 (62.5) | 342 (71.0) |  |
| Unknown | 1 (0.0) | 0 (0.0) | 0 (0.0) | 1 (0.1) | 0 (0.0) |  |
| **Memory-related diseases, n (%)** |  |  |  |  |  | <0.001 |
| No | 8596 (99.1) | 4874 (99.7) | 2309 (98.6) | 952 (99.0) | 461 (95.6) |  |
| Yes | 75 (0.9) | 13 (0.3) | 32 (1.4) | 9 (0.9) | 21 (4.4) |  |
| Unknown | 4 (0.0) | 2 (0.0) | 1 (0.0) | 1 (0.1) | 0 (0.0) |  |
| **Number of comorbidities, n (%)** |  |  |  |  |  | <0.001 |
| 0 | 2913 (33.6) | 1784 (36.5) | 779 (33.3) | 253 (26.3) | 97 (20.1) |  |
| 1 | 3210 (37.0) | 1844 (37.7) | 850 (36.3) | 345 (35.9) | 171 (35.5) |  |
| ≥ 2 | 2528 (29.1) | 1247 (25.5) | 708 (30.2) | 359 (37.3) | 214 (44.4) |  |
| Unknown | 24 (0.3) | 14 (0.3) | 5 (0.2) | 5 (0.5) | 0 (0.0) |  |
| **Falls status, n (%)** |  |  |  |  |  | <0.001 |
| No falls | 4309 (49.7) | 2621 (53.6) | 1197 (51.1) | 347 (36.1) | 144 (29.9) |  |
| Falls | 4366 (50.3) | 2268 (46.4) | 1145 (48.9) | 615 (63.9) | 338 (70.1) |  |
| **Injuries from falls** |  |  |  |  |  | <0.001 |
| No falls | 4309 (52.6) | 2621 (56.0) | 1197 (53.9) | 347 (40.5) | 144 (33.0) |  |
| Falls without injuries | 2550 (31.1) | 1396 (29.8) | 667 (30.0) | 305 (35.6) | 182 (41.6) |  |
| Falls with injuries | 1335 (16.3) | 663 (14.2) | 356 (16.0) | 205 (23.9) | 111 (25.4) |  |
| **Recurrent falls** |  |  |  |  |  | <0.001 |
| No falls | 3379 (53.5) | 2052 (57.3) | 952 (54.3) | 261 (41.3) | 114 (32.6) |  |
| Single falls | 1706 (27.0) | 979 (27.3) | 465 (26.5) | 177 (28.0) | 85 (24.3) |  |
| Multiple falls | 1231 (19.5) | 551 (15.4) | 335 (19.1) | 194 (30.7) | 151 (43.1) |  |
| **Falls with hip fracture** |  |  |  |  |  | <0.001 |
| No falls | 4227 (50.1) | 2584 (53.9) | 1177 (51.8) | 335 (36.6) | 131 (29.4) |  |
| Falls without hip fracture | 4120 (48.9) | 2184 (45.5) | 1070 (47.1) | 567 (61.9) | 299 (67.0) |  |
| Falls with hip fracture | 85 (1.0) | 29 (0.6) | 26 (1.1) | 14 (1.5) | 16 (3.6) |  |

*P* values were calculated by analysis of variance or chi-square test when appropriate;

Abbreviations: CHARLS, China Health and Retirement Longitudinal Study; ELSA, English Longitudinal Study on Ageing; HRS, US Health and Retirement Study.

**Table Z.** Associations between falls and joint trajectories of physical function, depressive symptoms, and cognitive performance by cohort

|  | **Group 2** | | | **Group 3** | | | **Group 4** | | |
| --- | --- | --- | --- | --- | --- | --- | --- | --- | --- |
|  | **CHARLS OR (95% CI)** | **ELSA OR (95% CI)** | **HRS OR (95% CI)** | **CHARLS OR (95% CI)** | **ELSA OR (95% CI)** | **HRS OR (95% CI)** | **CHARLS OR (95% CI)** | **ELSA OR (95% CI)** | **HRS OR (95% CI)** |
| **Fall status** |  |  |  |  |  |  |  |  |  |
| No falls | Ref. | Ref. | Ref. | Ref. | Ref. | Ref. | Ref. | Ref. | Ref. |
| Falls | 1.02 (0.81-1.29) | 1.05 (0.87-1.26) | **1.25 (1.05-1.50)** | **1.69 (1.23-2.32)** | **1.96 (1.51-2.56)** | **2.23 (1.76-2.82)** | **1.91 (1.18-3.09)** | **2.44 (1.67-3.57)** | **2.93 (2.11-4.07)** |
| **Falls with injuries** |  |  |  |  |  |  |  |  |  |
| No falls | Ref. | Ref. | Ref. | Ref. | Ref. | Ref. | Ref. | Ref. | Ref. |
| Falls without injuries | 0.97 (0.73-1.30) | 1.02 (0.83-1.26) | **1.24 (1.01-1.52)** | 1.45 (0.99-2.13) | **1.78 (1.32-2.41)** | **1.82 (1.38-2.38)** | **1.82 (1.04-3.17)** | **2.15 (1.39-3.31)** | **2.67 (1.85-3.85)** |
| Falls with injuries | 0.98 (0.72-1.35) | 1.14 (0.86-1.50) | **1.34 (1.03-1.75)** | 1.50 (0.99-2.25) | **2.13 (1.47-3.09)** | **2.65 (1.93-3.65)** | **1.91 (1.05-3.46)** | **3.03 (1.83-5.03)** | **3.10 (2.01-4.78)** |
| **Recurrent falls** |  |  |  |  |  |  |  |  |  |
| No falls | Ref. | Ref. | Ref. | Ref. | Ref. | Ref. | Ref. | Ref. | Ref. |
| Single fall | NA | 1.05 (0.86-1.29) | 1.05 (0.93-1.32) | NA | **1.46 (1.06-2.00)** | **1.49 (1.10-2.03)** | NA | 1.33 (0.82-2.14) | **1.74 (1.14-2.65)** |
| Multiple falls | NA | 1.26 (0.95-1.68) | **1.51 (1.04-2.18)** | NA | **2.82 (1.96-4.07)** | **2.94 (2.20-3.93)** | NA | **4.85 (3.06-7.67)** | **4.77 (3.25-7.01)** |
| **Falls with hip fracture** |  |  |  |  |  |  |  |  |  |
| No falls | Ref. | Ref. | Ref. | Ref. | Ref. | Ref. | Ref. | Ref. | Ref. |
| Falls without hip fracture | 1.00 (0.79-1.27) | 1.03 (0.86-1.24) | **1.23 (1.02-1.47)** | **1.59 (1.14-2.20)** | **2.02 (1.54-2.65)** | **2.20 (1.73-2.80)** | **1.02 (1.20-3.41)** | **2.41 (1.63-3.58)** | **2.96 (2.10-4.17)** |
| Falls with hip fracture | 0.49 (0.15-1.61) | **3.53 (1.02-12.20)** | 2.29 (0.92-5.73) | 1.81 (0.51-6.40) | NA | **4.93 (1.87-13.03)** | **3.94 (0.97-15.93)** | **15.26 (3.12-74.67)** | **7.06 (2.26-22.10)** |

ORs were estimated using multinomial logistic regression models with Group 1 as the reference outcome category, adjusted for age, sex, education level, total household wealth, marital status, current smoking status, current alcohol consumption, physical activity, stroke, arthritis, memory-related diseases, eyesight, hearing, and number of other comorbidities;

Group 1: Favorable trajectories of physical function, depressive symptoms, and cognitive performance; Group 2: Favorable trajectories of physical function and depressive symptoms, combined with low and declining cognitive performance; Group 3: Favorable trajectory of physical function, but poor and deteriorating depressive symptoms and cognitive performance; Group 4: Rapidly declining physical function, combined with poor and worsening depressive symptoms and cognitive performance;

**Bolded text indicates statistically significant;**

Abbreviations: OR, odds ratio; CI, confidence interval; NA, not applicable.

**Table AA.** Subgroup analyses of the associations between falls and joint trajectories of physical function, depressive symptoms, and cognitive performance

|  | **Group 2** | | **Group 3** | | **Group 4** | |
| --- | --- | --- | --- | --- | --- | --- |
|  | **OR (95% CI)** | ***P*-interaction** | **OR (95% CI)** | ***P*-interaction** | **OR (95% CI)** | ***P*-interaction** |
| **Age (< 75 years)** |  |  |  |  |  |  |
| No falls | Ref. |  | Ref. |  | Ref. |  |
| Falls | 1.08 (0.95-1.23) |  | **1.81 (1.51-2.16)** |  | **2.16 (1.63-2.86)** |  |
| **Age (≥ 75 years)** |  |  |  |  |  |  |
| No falls | Ref. |  | Ref. |  | Ref. |  |
| Falls | 1.28 (1.05-1.57) | 0.159 | **2.61 (1.94-3.52)** | **0.042** | **3.21 (2.25-4.58)** | 0.129 |
| **Male** |  |  |  |  |  |  |
| No falls | Ref. |  | Ref. |  | Ref. |  |
| Falls | **1.18 (1.01-1.38)** |  | **1.91 (1.49-2.46)** |  | **2.92 (2.08-4.09)** |  |
| **Female** |  |  |  |  |  |  |
| No falls | Ref. |  | Ref. |  | Ref. |  |
| Falls | 1.06 (0.90-1.24) | 0.295 | **2.00 (1.65-2.42)** | 0.861 | **2.16 (1.62-1.89)** | 0.157 |
| **Primary education level** |  |  |  |  |  |  |
| No falls | Ref. |  | Ref. |  | Ref. |  |
| Falls | 1.16 (0.98-1.37) |  | **2.10 (1.67-2.64)** |  | **2.30 (1.68-3.15)** |  |
| **Secondary education level** |  |  |  |  |  |  |
| No falls | Ref. |  | Ref. |  | Ref. |  |
| Falls | 1.13 (0.95-1.34) | 0.916 | **1.77 (1.40-2.23)** | 0.342 | **2.65 (1.87-3.74)** | 0.544 |
| **Tertiary education level** |  |  |  |  |  |  |
| No falls | Ref. |  | Ref. |  | Ref. |  |
| Falls | 0.92 (0.64-1.32) | 0.267 | **3.35 (1.93-5.81)** | 0.091 | 2.18 (0.98-4.84) | 0.860 |
| **Lower wealth** |  |  |  |  |  |  |
| No falls | Ref. |  | Ref. |  | Ref. |  |
| Falls | 1.04 (0.89-1.21) |  | **1.86 (1.53-2.27)** |  | **2.22 (1.70-2.91)** |  |
| **Upper wealth** |  |  |  |  |  |  |
| No falls | Ref. |  | Ref. |  | Ref. |  |
| Falls | 1.16 (0.99-1.36) | 0.307 | **2.06 (1.61-2.63)** | 0.501 | **3.00 (2.02-4.45)** | 0.158 |

ORs were estimated using multinomial logistic regression models with Group 1 as the reference outcome category, adjusted for age, sex, cohort, education level, total household wealth, marital status, current smoking status, current alcohol consumption, physical activity, stroke, arthritis, memory-related diseases, eyesight, hearing, and number of other comorbidities;

Group 1: Favorable trajectories of physical function, depressive symptoms, and cognitive performance; Group 2: Favorable trajectories of physical function and depressive symptoms, combined with low and declining cognitive performance; Group 3: Favorable trajectory of physical function, but poor and deteriorating depressive symptoms and cognitive performance; Group 4: Rapidly declining physical function, combined with poor and worsening depressive symptoms and cognitive performance;

**Bolded text indicates statistically significant;**

Abbreviations: OR, odds ratio; CI, confidence interval.

**Table BB.** Subgroup analyses of the associations between falls with injuries and joint trajectories of physical function, depressive symptoms, and cognitive performance

|  | **Group 2** | | **Group 3** | | **Group 4** | |
| --- | --- | --- | --- | --- | --- | --- |
|  | **OR (95% CI)** | ***P*-interaction** | **OR (95% CI)** | ***P*-interaction** | **OR (95% CI)** | ***P*-interaction** |
| **Age (< 75 years)** |  |  |  |  |  |  |
| No falls | Ref. |  | Ref. |  | Ref. |  |
| Falls without injuries | 1.02 (0.88-1.19) |  | **1.56 (1.27-1.92)** |  | **1.94 (1.41-2.66)** |  |
| Falls with injuries | 1.17 (0.96-1.42) |  | **1.90 (1.48-2.43)** |  | **2.37 (1.63-3.44)** |  |
| **Age (≥ 75 years)** |  |  |  |  |  |  |
| No falls | Ref. |  | Ref. |  | Ref. |  |
| Falls without injuries | **1.29 (1.02-1.64)** | 0.110 | **2.21 (1.55-3.14)** | 0.117 | **3.07 (2.06-4.58)** | 0.116 |
| Falls with injuries | 1.25 (0.94-1.66) | 0.568 | **2.72 (1.86-3.98)** | 0.117 | **3.34 (2.14-5.23)** | 0.261 |
| **Male** |  |  |  |  |  |  |
| No falls | Ref. |  | Ref. |  | Ref. |  |
| Falls without injuries | **1.20 (1.00-1.43)** |  | **1.80 (1.36-2.38)** |  | **2.63 (1.82-3.80)** |  |
| Falls with injuries | 1.13 (0.88-1.44) |  | **1.72 (1.18-2.49)** |  | **3.44 (2.22-5.35)** |  |
| **Female** |  |  |  |  |  |  |
| No falls | Ref. |  | Ref. |  | Ref. |  |
| Falls without injuries | 0.97 (0.80-1.17) | 0.065 | **1.56 (1.24-1.96)** | 0.334 | **1.99 (1.42-2.79)** | 0.227 |
| Falls with injuries | 1.20 (0.97-1.49) | 0.469 | **2.32 (1.81-2.97)** | 0.128 | **2.22 (1.52-3.23)** | 0.134 |
| **Primary education level** |  |  |  |  |  |  |
| No falls | Ref. |  | Ref. |  | Ref. |  |
| Falls without injuries | 1.15 (0.94-1.40) |  | **1.81 (1.38-2.38)** |  | **2.10 (1.46-3.01)** |  |
| Falls with injuries | 1.08 (0.85-1.38) |  | **2.06 (1.51-2.81)** |  | **2.32 (1.54-3.51)** |  |
| **Secondary education level** |  |  |  |  |  |  |
| No falls | Ref. |  | Ref. |  | Ref. |  |
| Falls without injuries | 1.06 (0.88-1.29) | 0.940 | **1.43 (1.09-1.88)** | 0.288 | **2.46 (1.67-3.61)** | 0.552 |
| Falls with injuries | 1.29 (1.00-1.66) | 0.465 | **2.18 (1.59-2.99)** | 0.906 | **3.03 (1.93-4.75)** | 0.435 |
| **Tertiary education level** |  |  |  |  |  |  |
| No falls | Ref. |  | Ref. |  | Ref. |  |
| Falls without injuries | 0.99 (0.65-1.50) | 0.443 | **3.34 (1.83-6.09)** | 0.073 | 2.03 (0.82-5.07) | 0.692 |
| Falls with injuries | 0.96 (0.56-1.65) | 0.824 | **3.22 (1.60-6.49)** | 0.163 | 2.69 (0.98-7.42) | 0.807 |
| **Lower wealth** |  |  |  |  |  |  |
| No falls | Ref. |  | Ref. |  | Ref. |  |
| Falls without injuries | 1.03 (0.86-1.23) |  | **1.50 (1.19-1.89)** |  | **1.92 (1.41-2.61)** |  |
| Falls with injuries | 1.05 (0.83-1.31) |  | **2.08 (1.59-2.72)** |  | **2.40 (1.68-3.45)** |  |
| **Upper wealth** |  |  |  |  |  |  |
| No falls | Ref. |  | Ref. |  | Ref. |  |
| Falls without injuries | 1.08 (0.90-1.31) | 0.742 | **1.88 (1.42-2.49)** | 0.252 | **2.98 (1.94-1.40)** | 0.069 |
| Falls with injuries | **1.28 (1.02-1.61)** | 0.152 | **2.06 (1.48-2.87)** | 0.0.974 | **3.47 (2.13-5.65)** | 0.202 |

ORs were estimated using multinomial logistic regression models with Group 1 as the reference outcome category, adjusted for age, sex, cohort, education level, total household wealth, marital status, current smoking status, current alcohol consumption, physical activity, stroke, arthritis, memory-related diseases, eyesight, hearing, and number of other comorbidities;

Group 1: Favorable trajectories of physical function, depressive symptoms, and cognitive performance; Group 2: Favorable trajectories of physical function and depressive symptoms, combined with low and declining cognitive performance; Group 3: Favorable trajectory of physical function, but poor and deteriorating depressive symptoms and cognitive performance; Group 4: Rapidly declining physical function, combined with poor and worsening depressive symptoms and cognitive performance;

**Bolded text indicates statistically significant;**

Abbreviations: OR, odds ratio; CI, confidence interval.

**Table CC.** Subgroup analyses of the associations between recurrent falls and joint trajectories of physical function, depressive symptoms, and cognitive performance

|  | **Group 2** | | **Group 3** | | **Group 4** | |
| --- | --- | --- | --- | --- | --- | --- |
|  | **OR (95% CI)** | ***P*-interaction** | **OR (95% CI)** | ***P*-interaction** | **OR (95% CI)** | ***P*-interaction** |
| **Age (< 75 years)** |  |  |  |  |  |  |
| No falls | Ref. |  | Ref. |  | Ref. |  |
| Single fall | 1.00 (0.82-1.21) |  | **1.33 (1.01-1.74)** |  | 1.36 (0.87-2.14) |  |
| Multiple falls | **1.29 (1.03-1.61)** |  | **2.36 (1.53-4.25)** |  | **3.57 (2.37-5.38)** |  |
| **Age (≥ 75 years)** |  |  |  |  |  |  |
| No falls | Ref. |  | Ref. |  | Ref. |  |
| Single fall | 1.20 (0.93-1.54) | 0.137 | **1.76 (1.21-2.57)** | 0.237 | **2.00 (1.24-3.08)** | 0.203 |
| Multiple falls | **1.75 (1.31-2.34)** | 0.224 | **4.26 (2.91-6.24)** | **0.016** | **7.09 (4.58-10.97)** | 0.055 |
| **Male** |  |  |  |  |  |  |
| No falls | Ref. |  | Ref. |  | Ref. |  |
| Single fall | **1.23 (0.98-1.53)** |  | **1.51 (1.04-2.20)** |  | **2.10 (1.25-3.51)** |  |
| Multiple falls | **1.38 (1.07-1.77)** |  | **2.15 (1.47-3.14)** |  | **5.32 (3.33-8.51)** |  |
| **Female** |  |  |  |  |  |  |
| No falls | Ref. |  | Ref. |  | Ref. |  |
| Single fall | 0.90 (0.73-1.12) | **0.034** | **1.37 (1.04-1.79)** | 0.746 | 1.23 (0.82-1.84) | 0.079 |
| Multiple falls | **1.39 (1.07-1.80)** | 0.819 | **3.35 (2.52-4.46)** | 0.050 | **4.45 (3.03-6.52)** | 0.553 |
| **Primary education level** |  |  |  |  |  |  |
| No falls | Ref. |  | Ref. |  | Ref. |  |
| Single fall | 1.16 (0.86-1.57) |  | **1.75 (1.14-2.68)** |  | 1.50 (0.85-2.64) |  |
| Multiple falls | **2.12 (1.41-3.19)** |  | **4.65 (2.83-7.66)** |  | **7.20 (4.10-12.65)** |  |
| **Secondary education level** |  |  |  |  |  |  |
| No falls | Ref. |  | Ref. |  | Ref. |  |
| Single fall | 1.09 (0.89-1.34) | 0.937 | 1.33 (0.99-1.78) | 0.482 | **1.65 (1.07-2.55)** | 0.559 |
| Multiple falls | **1.33 (1.05-1.68)** | 0.076 | **2.55 (1.90-3.43)** | 0.060 | **4.54 (3.03-6.80)** | 0.257 |
| **Tertiary education level** |  |  |  |  |  |  |
| No falls | Ref. |  | Ref. |  | Ref. |  |
| Single fall | 0.68 (0.42-1.10) | 0.201 | **2.04 (1.04-3.98)** | 0.360 | 1.32 (0.46-3.78) | 0.975 |
| Multiple falls | 1.23 (0.75-2.04) | **0.049** | **3.52 (1.75-7.07)** | 0.436 | **3.11 (1.16-8.32)** | 0.092 |
| **Lower wealth** |  |  |  |  |  |  |
| No falls | Ref. |  | Ref. |  | Ref. |  |
| Single fall | 1.02 (0.82-1.26) |  | **1.51 (1.14-2.00)** |  | 1.22 (0.82-1.80) |  |
| Multiple falls | 1.22 (0.94-1.57) |  | **2.56 (1.89-3.45)** |  | **4.30 (3.01-6.15)** |  |
| **Upper wealth** |  |  |  |  |  |  |
| No falls | Ref. |  | Ref. |  | Ref. |  |
| Single fall | 1.06 (0.85-1.33) | 0.541 | 1.30 (0.89-1.88) | 0.599 | **2.70 (1.51-4.82)** | **0.011** |
| Multiple falls | **1.52 (1.18-1.96)** | 0.237 | **3.10 (2.18-4.40)** | 0.424 | **5.63 (3.20-9.91)** | 0.360 |

ORs were estimated using multinomial logistic regression models with Group 1 as the reference outcome category, adjusted for age, sex, cohort, education level, total household wealth, marital status, current smoking status, current alcohol consumption, physical activity, stroke, arthritis, memory-related diseases, eyesight, hearing, and number of other comorbidities;

Group 1: Favorable trajectories of physical function, depressive symptoms, and cognitive performance; Group 2: Favorable trajectories of physical function and depressive symptoms, combined with low and declining cognitive performance; Group 3: Favorable trajectory of physical function, but poor and deteriorating depressive symptoms and cognitive performance; Group 4: Rapidly declining physical function, combined with poor and worsening depressive symptoms and cognitive performance;

**Bolded text indicates statistically significant;**

Abbreviations: OR, odds ratio; CI, confidence interval.

**Table DD.** Subgroup analyses of the associations between falls with hip fracture and joint trajectories of physical function, depressive symptoms, and cognitive performance

|  | **Group 2** | | **Group 3** | | **Group 4** | |
| --- | --- | --- | --- | --- | --- | --- |
|  | **OR (95% CI)** | ***P*-interaction** | **OR (95% CI)** | ***P*-interaction** | **OR (95% CI)** | ***P*-interaction** |
| **Age (< 75 years)** |  |  |  |  |  |  |
| No falls | Ref. |  | Ref. |  | Ref. |  |
| Falls without hip fracture | 1.08 (0.94-1.23) |  | **1.80 (1.50-2.16)** |  | **2.16 (1.62-2.91)** |  |
| Falls with hip fracture | 0.96 (0.42-2.21) |  | 1.76 (0.70-4.44) |  | **5.70 (2.09-15.53)** |  |
| **Age (≥ 75 years)** |  |  |  |  |  |  |
| No falls | Ref. |  | Ref. |  | Ref. |  |
| Falls without hip fracture | **1.23 (1.00-1.51)** | 0.301 | **2.61 (1.92-3.55)** | 0.053 | **3.30 (2.27-4.81)** | 0.155 |
| Falls with hip fracture | **3.40 (1.33-8.69)** | 0.061 | **4.77 (1.55-14.65)** | 0.163 | **11.59 (3.69-36.38)** | 0.342 |
| **Male** |  |  |  |  |  |  |
| No falls | Ref. |  | Ref. |  | Ref. |  |
| Falls without hip fracture | 1.17 (0.99-1.37) |  | **1.92 (1.49-2.48)** |  | **3.23 (2.26-4.63)** |  |
| Falls with hip fracture | 1.76 (0.72-4.27) |  | 1.67 (0.43-6.49) |  | **7.53 (2.36-24.06)** |  |
| **Female** |  |  |  |  |  |  |
| No falls | Ref. |  | Ref. |  | Ref. |  |
| Falls without hip fracture | 1.02 (0.88-1.21) | 0.215 | **1.97 (1.62-2.40)** | 0.965 | **2.06 (1.52-2.79)** | **0.041** |
| Falls with hip fracture | 1.41 (0.65-3.10) | 0.990 | **2.89 (1.25-6.67)** | 0.480 | **6.13 (2.37-15.87)** | 0.909 |
| **Primary education level** |  |  |  |  |  |  |
| No falls | Ref. |  | Ref. |  | Ref. |  |
| Falls without hip fracture | 1.13 (0.95-1.34) |  | **2.03 (1.60-2.58)** |  | **2.36 (1.69-3.27)** |  |
| Falls with hip fracture | 1.21 (0.52-2.79) |  | 1.88 (0.65-5.45) |  | **5.05 (1.81-14.08)** |  |
| **Secondary education level** |  |  |  |  |  |  |
| No falls | Ref. |  | Ref. |  | Ref. |  |
| Falls without hip fracture | 1.12 (0.94-1.33) | 0.952 | **1.76 (1.39-2.23)** | 0.451 | **2.66 (1.86-3.82)** | 0.552 |
| Falls with hip fracture | 2.10 (0.84-5.27) | 0.308 | **3.69 (1.31-10.41)** | 0.309 | **7.39 (2.20-24.85)** | 0.662 |
| **Tertiary education level** |  |  |  |  |  |  |
| No falls | Ref. |  | Ref. |  | Ref. |  |
| Falls without hip fracture | 0.89 (0.61-1.30) | 0.244 | **3.49 (1.98-6.15)** | 0.070 | **2.42 (1.04-5.62)** | 0.926 |
| Falls with hip fracture | 1.02 (0.14-7.27) | 0.693 | NA | NA | NA | NA |
| **Lower wealth** |  |  |  |  |  |  |
| No falls | Ref. |  | Ref. |  | Ref. |  |
| Falls without hip fracture | 1.01 (0.86-1.18) |  | **1.82 (1.48-2.22)** |  | 2.09 (1.58-2.77) |  |
| Falls with hip fracture | 1.69 (0.68-4.23) |  | **3.29 (1.23-8.85)** |  | **9.31 (3.48-24.89)** |  |
| **Upper wealth** |  |  |  |  |  |  |
| No falls | Ref. |  | Ref. |  | Ref. |  |
| Falls without hip fracture | 1.15 (0.98-1.35) | 0.215 | **2.09 (1.63-2.69)** | 0.380 | **3.41 (2.23-5.22)** | **0.035** |
| Falls with hip fracture | 1.52 (0.69-3.35) | 0.920 | 2.21 (0.75-6.45) | 0.671 | 3.00 (0.73-12.43) | 0.367 |

ORs were estimated using multinomial logistic regression models with Group 1 as the reference outcome category, adjusted for age, sex, cohort, education level, total household wealth, marital status, current smoking status, current alcohol consumption, physical activity, stroke, arthritis, memory-related diseases, eyesight, hearing, and number of other comorbidities;

Group 1: Favorable trajectories of physical function, depressive symptoms, and cognitive performance; Group 2: Favorable trajectories of physical function and depressive symptoms, combined with low and declining cognitive performance; Group 3: Favorable trajectory of physical function, but poor and deteriorating depressive symptoms and cognitive performance; Group 4: Rapidly declining physical function, combined with poor and worsening depressive symptoms and cognitive performance;

**Bolded text indicates statistically significant;**

Abbreviations: OR, odds ratio; CI, confidence interval; NA, not applicable.

**Table EE.** Sensitivity analysis 1 for joint trajectory analyses: Conducting inverse probability weighting analysis

|  | **Group 1** | **Group 2** | **Group 3** | **Group 4** |
| --- | --- | --- | --- | --- |
|  | **OR (95% CI)** | **OR (95% CI)** | **OR (95% CI)** | **OR (95% CI)** |
| **Fall status** |  |  |  |  |
| No falls | Ref. | Ref. | Ref. | Ref. |
| Falls | Ref. | 1.11 (1.00-1.24) | **1.98 (1.70-2.30)** | **2.40 (1.95-2.96)** |
| **Falls with injuries** |  |  |  |  |
| No falls | Ref. | Ref. | Ref. | Ref. |
| Falls without injuries | Ref. | 1.08 (0.95-1.22) | **1.67 (1.40-1.99)** | **2.21 (1.74-2.79)** |
| Falls with injuries | Ref. | 1.14 (0.98-1.34) | **2.12 (1.73-2.59)** | **2.56 (1.96-3.36)** |
| **Recurrent falls** |  |  |  |  |
| No falls | Ref. | Ref. | Ref. | Ref. |
| Single fall | Ref. | 1.05 (0.89-1.23) | **1.46 (1.17-1.83)** | **1.51 (1.10-2.07)** |
| Multiple falls | Ref. | **1.41 (1.17-1.70)** | **2.90 (2.30-3.67)** | **4.80 (3.59-6.42)** |
| **Falls with hip fracture** |  |  |  |  |
| No falls | Ref. | Ref. | Ref. | Ref. |
| Falls without hip fracture | Ref. | 1.09 (0.97-1.22) | **1.96 (1.68-2.28)** | **2.44 (1.96-3.04)** |
| Falls with hip fracture | Ref. | 1.35 (0.78-2.33) | **2.37 (1.24-4.42)** | **5.63 (2.89-10.97)** |

Models were adjusted for age, sex, cohort, education level, total household wealth, marital status, current smoking status, current alcohol consumption, physical activity, stroke, arthritis, memory-related diseases, eyesight, hearing, and number of other comorbidities;

Group 1: Favorable trajectories of physical function, depressive symptoms, and cognitive performance; Group 2: Favorable trajectories of physical function and depressive symptoms, combined with low and declining cognitive performance; Group 3: Favorable trajectory of physical function, but poor and deteriorating depressive symptoms and cognitive performance; Group 4: Rapidly declining physical function, combined with poor and worsening depressive symptoms and cognitive performance;

**Bolded text indicates statistically significant;**

Abbreviations: OR, odds ratio; CI, confidence interval.

**Table FF.** Sensitivity analysis 2 for joint trajectory analyses: Imputing missing covariates using multiple imputation

|  | **Group 1** | **Group 2** | **Group 3** | **Group 4** |
| --- | --- | --- | --- | --- |
|  | **OR (95% CI)** | **OR (95% CI)** | **OR (95% CI)** | **OR (95% CI)** |
| **Fall status** |  |  |  |  |
| No falls | Ref. | Ref. | Ref. | Ref. |
| Falls | Ref. | **1.12 (1.00-1.25)** | **1.98 (1.70-2.30)** | **2.42 (1.94-3.00)** |
| **Falls with injuries** |  |  |  |  |
| No falls | Ref. | Ref. | Ref. | Ref. |
| Falls without injuries | Ref. | 1.09 (0.96-1.23) | **1.67 (1.40-1.99)** | **2.22 (1.97-3.47)** |
| Falls with injuries | Ref. | 1.17 (0.99-1.37) | **2.13 (1.73-2.61)** | **2.61 (1.97-3.47)** |
| **Recurrent falls** |  |  |  |  |
| No falls | Ref. | Ref. | Ref. | Ref. |
| Single fall | Ref. | 1.04 (0.90-1.22) | **1.44 (1.16-1.79)** | **1.55 (1.13-2.12)** |
| Multiple falls | Ref. | **1.39 (1.16-1.66)** | **2.86 (2.28-3.58)** | **4.60 (3.44-6.16)** |
| **Falls with hip fracture** |  |  |  |  |
| No falls | Ref. | Ref. | Ref. | Ref. |
| Falls without hip fracture | Ref. | 1.10 (0.98-1.23) | **1.96 (1.68-2.29)** | **2.45 (1.95-3.07)** |
| Falls with hip fracture | Ref. | 1.48 (0.83-2.65) | **2.47 (1.23-4.93)** | **5.99 (2.89-12.39)** |

Models were adjusted for age, sex, cohort, education level, total household wealth, marital status, current smoking status, current alcohol consumption, physical activity, stroke, arthritis, memory-related diseases, eyesight, hearing, and number of other comorbidities;

Group 1: Favorable trajectories of physical function, depressive symptoms, and cognitive performance; Group 2: Favorable trajectories of physical function and depressive symptoms, combined with low and declining cognitive performance; Group 3: Favorable trajectory of physical function, but poor and deteriorating depressive symptoms and cognitive performance; Group 4: Rapidly declining physical function, combined with poor and worsening depressive symptoms and cognitive performance;

**Bolded text indicates statistically significant;**

Abbreviations: OR, odds ratio; CI, confidence interval.

**Fig A.** Physical function, depressive symptoms, and cognitive performance trajectories fitted using local polynomial regression by fall status.

**Fig B.** Physical function, depressive symptoms, and cognitive performance trajectories fitted using local polynomial regression by injuries from falls.

**Fig C.** Physical function, depressive symptoms, and cognitive performance trajectories fitted using local polynomial regression by recurrent falls.

**Fig D.** Physical function, depressive symptoms, and cognitive performance trajectories fitted using local polynomial regression by recurrent falls.

**Fig E.** Trajectories of physical function, depressive symptoms, and cognitive performance before and after falls and fall-related characterizations using spline-based mixed-effects models.

Models were adjusted age, sex, cohort, education level, total household wealth, marital status, current smoking status, current alcohol consumption, physical activity, stroke, arthritis, memory-related diseases, eyesight, hearing, and number of other comorbidities;

Pre_fall internal knot: time = -2.25; and post_fall internal knot: time = 2.92.
