# Supplementary material for: Trajectories of physical function, depressive symptoms, and cognitive performance before and after falls in older adults: A matched multicohort study
Source: PLoS Med. 2026 Aug 3;23(8):e1005083. doi: 10.1371/journal.pmed.1005083 (PMC13446737; doi:10.1371/journal.pmed.1005083)
Supplement: Supplementary file 3 [file pmed.1005083.s003.docx]

**Manuscript ID:** PMEDICINE-D-26-01351R1

**Title:** Trajectories of physical, psychological, and cognitive functioning before and after falls in older adults: a matched multicohort study

Dear Dr Katarzyna Starowicz,

Thank you for reviewing our manuscript and giving us the opportunity to revise and resubmit. We would like to thank the editors and reviewers for their thorough appraisals and comments. We have considered the editors’ and the reviewers’ comments in the point-by-point reply. **Our response is given in the blue text** and changes/additions to the **manuscript are given in the red text**.

Dr. Xiaolin Xu, on behalf of all authors

**Response to the academic editor**

**Comment 1:** This is a nicely written study that is of clinical interest and appears to fill a gap in the literature. The reviewers have picked up on a range of appropriate issue. One extra issue I would like to raise is the measurement of physical function. It is not really clear from the methods how it was measured as it does not appear that any of the cohorts used a validated measure. The authors should provide the actual questions and response items used in each cohort for greater clarity. The use of "difficulty controlling urination & defecation" in place of difficulty walking across the room in CHARLS does not seem appropriate to me and needs explanation. Do the results change if this item was omitted from the physical function score? I am not sure most people would agree that urinary/defecation control should be considered "physical" function and grouped with the other items "walking across a room, dressing, bathing, eating, getting in and out of bed, and using the toilet".

**Response:** Thank you for your assessment and for raising this important issue. We agree that the measurement of physical functioning needed to be described more clearly. We have added details of the Activities of Daily Living (ADL) measures in the Methods section and in Table S1.

Regarding the validity of the ADL measures, the items used in CHARLS, ELSA, and HRS are based on widely used basic ADL and physical functioning frameworks. The Katz Index of Activities of Daily Living includes bathing, dressing, toileting, transferring, continence, and feeding, and is one of the classic instruments for assessing basic functional status in older adults [Katz et al., 1963; Wallace and Shelkey, 2007]. The Barthel Index similarly includes bowel and bladder control, toilet use, transfers, mobility, feeding, bathing, grooming, dressing, and stairs as core components of ADL assessment [Mahoney and Barthel, 1965]. In HRS, physical functioning documentation has also shown that walking across a room, dressing, eating, bathing, and getting in and out of bed load on the ADL factor, supporting their use as physical functioning/ADL measures in this cohort [Fonda et al., 2004]. ADL items in ELSA are consistent with the HRS-family physical functioning framework.

Therefore, we retained the CHARLS continence item in the physical functioning score rather than omitting it from the main analysis, because removing a recognized ADL component could also reduce the content validity of the ADL measure in CHARLS. At the same time, we agree that continence is not directly equivalent to walking across a room and that differences in item composition across cohorts may affect comparability [Sanses et al., 2017].

Previous harmonized HRS-family studies have generally acknowledged differences in ADL item composition across cohorts. We further used cohort-specific z-scores, together with adjustment for cohort in the models, to improve comparability across cohorts. Nevertheless, standardization cannot fully eliminate measurement differences or prevent loss of original scale information [Lee et al., 2021; Gateway to Global Aging Data; Chan et al., 2012].

**Nevertheless, we conducted an additional sensitivity analysis redefining physical functioning using the five ADL items common to all cohorts** (Table S21)**. The results remained robust, and we have also discussed this limitation in the revised manuscript** (Page 21, Lines 424-425)**.**

Katz S, Ford AB, Moskowitz RW, Jackson BA, Jaffe MW. Studies of illness in the aged: the index of ADL: a standardized measure of biological and psychosocial function. JAMA. 1963;185:914–919.

Mahoney FI, Barthel DW. Functional evaluation: the Barthel Index. Md State Med J. 1965;14:61–65.

Wallace M, Shelkey M. Katz Index of Independence in Activities of Daily Living (ADL). Urol Nurs. 2007;27:93–94.

Fonda S, Wallace RB, Herzog AR. Documentation of physical functioning measured in the Health and Retirement Study and the Asset and Health Dynamics among the Oldest Old Study. HRS/AHEAD Documentation Report. 2004.

Chan KS, Kasper JD, Brandt J, Pezzin LE. Measurement equivalence in ADL and IADL difficulty across international surveys of aging: findings from the HRS, SHARE, and ELSA. J Gerontol B Psychol Sci Soc Sci. 2012;67B:121–132. doi:10.1093/geronb/gbr133.

Sanses TVD, Rantell A, Brown J, Panicker JN. The relationship between urinary incontinence, mobility limitations, and disability in older women. Curr Geriatr Rep. 2017;6:74–80.

Lee J, Phillips D, Wilkens J, Gateway to Global Aging Data Team. Gateway to Global Aging Data: resources for cross-national comparisons of family, social environment, and healthy aging. J Gerontol B Psychol Sci Soc Sci. 2021;76:S5–S16. doi:10.1093/geronb/gbab050.

(Page 21, Lines 427-431) Fifth, between-cohort differences (e.g., mean age and the instruments used to assess functioning) should be taken into account. Therefore, cohort-specific interpretations may still be more appropriate in certain contexts. In addition, although z-scores are used to harmonize such heterogeneity, some valuable original information may have been lost.

**Comment 2:** Perhaps the authors have taken "using the toilet" literally but in a physical function context that would usually mean how difficult is it to get on/off the toilet.... Comorbid conditions have been included as a covariate- but they have been included as crude "number of conditions" and thus assume each condition is equally as important/influential as the other. For example, I would consider stroke to be potentially much more impactful on falls risk than diabetes. The authors should justify this approach and/or consider including key comorbidities separately, rather than simply as a crude "count"

**Response:** Thank you for your comment. We agree that “difficulties in getting on and off the toilet” is a more appropriate description rather than extracting the original question, and we have revised the corresponding text.

We also agree that using only the number of comorbidities may not fully capture the different impacts of specific conditions. Following your suggestion and after carefully reviewing previous studies, we additionally included key comorbidities closely related to falls, including stroke, arthritis, and memory-related disease, as separate covariates in the models [Kathryn et al., 2014; Stephanie et al., 2025]. To avoid potential overadjustment, particularly in some small subgroups, other conditions, including hypertension, diabetes, heart disease, and chronic lung disease (i.e., cardio-metabolic-respiratory conditions), were still adjusted for as part of the comorbidity count, an approach also suggested by some studies [Kathryn et al., 2014; Lawlor et al., 2023]. As also suggested by the reviewers, we further adjusted for memory-related disease, eyesight, and hearing. We have revised the **Covariates** section accordingly, provided detailed definitions in **Table S1, and updated the all results.**

Sibley KM, Voth J, Munce SE, Straus SE, Jaglal SB. Chronic disease and falls in community-dwelling Canadians over 65 years old: a population-based study exploring associations with number and pattern of chronic conditions. BMC Geriatr. 2014;14: 22. doi:10.1186/1471-2318-14-22.

Saunders S, D’Amore C, Hao Q, El-Moneim NA, Richardson J, Kuspinar A, et al. Risk Factors for Falls in Community-Dwelling Older Adults: An Umbrella Review. J Am Med Dir Assoc. 2025;26: 105765. doi:10.1016/j.jamda.2025.105765.

(Page 11, Lines191-200) Covariates were drawn from the baseline wave and included sociodemographic characteristics (age, sex, education level [primary, secondary, tertiary], net household wealth [Q1–Q4], and marital status [married or partnered; widowed; separate or divorced or single]), health-related lifestyle factors (current smoking status, alcohol consumption, and physical activity), eyesight, hearing, and chronic conditions (hypertension, diabetes, chronic lung disease, heart problem, stroke, arthritis, cancer, and memory-related diseases). Given their particular relevance to falls, stroke, arthritis, and memory-related disease were included as separate covariates, while other chronic conditions were summarized as the number of comorbidities (0, 1, ≥2). Missing values for covariates were categorized as “unknown”. Harmonized covariate classifications are shown in the appendix **(Table S1 in S1 Appendix).**

**Response to the reviewers**

**Reviewer #1**

Thank you for the opportunity to review your manuscript! The results are very interesting and important for falls prevention. The implication that prevention should start as early as possible is particularly relevant. I have provided a detailed assessment; however, most of my comments mainly relate to the use of more precise and inclusive language.

**Response:** We sincerely thank you for your detailed and valuable comments. We have carefully revised the manuscript accordingly, and we believe these revisions have improved the precision and inclusiveness of the language throughout following your comments and suggestions.

**Comment 1:** 23 and the following: I would suggest a change in wording, as the term "fallers" is not appropriate. For further information, please see: Preferred language when referring to older people in a health context | British Geriatrics Society

**Response:** We have revised the relevant wording throughout the manuscript to align with preferred person-first language in health contexts. Specifically, we replaced “fallers” with “participants with lived falls experience” or “participants with falls” as appropriate.

**Comment 2:** 35 fall onset: I would suggest changing the wording, as "onset" is not commonly used for falls but rather for diseases.

**Response:** We have also revised the relevant wording throughout the manuscript and replaced “fall onset” with more appropriate phrasing.

**Comment 3:** 82 The description of activity restriction following a fall could be elaborated further. The cited report refers to restricted activity for only one day and does not specify the type of activity (e.g., social or physical). I would therefore suggest citing an alternative source, such as one addressing medical attention or reductions in physical activity.

**Response:** We have cited an alternative source reporting health-service use, treatment, and declines in social and physical activities following a fall, and revised the sentence accordingly.

(Page 6, Lines 86-88) Studies indicate that, among older adults who experienced a fall, 23.5% used health services, 17.2% received treatment, and more than 15% experienced declines in social and physical activities.

**Comment 4:** 82 Did you include hip fractures because they are the most common injury after a fall? This clarification would be important, particularly as fall severity is later operationalised using hip fractures rather than other injuries, such as traumatic brain injuries.

**Response:** Thank you for this comment. We considered hip fractures because they are the most common and severe injuries resulting from falls as you mentioned. However, the choice was also influenced by data availability. We have added this clarification in the Limitations section. Please also see our response to **Comment 14** for further details.

(Pages 21-22, Lines 429-430) Seventh, data unavailability limited our ability to consider or differentiate other indicators related to fall severity.

**Comment 5:** 83 The term "psychological problems" is vague. Please specify the condition (e.g., depression) and consider using more precise and inclusive language. I would argue that the aforementioned risk factors are only partially preventable and would suggest softer wording.

**Response:** Thank you for this suggestion. We have revised “psychological problems” to “depressive symptoms” and softened the wording from “many of which are preventable” to “some of which are partially preventable.”

**Comment 6:** 88 You stated that many studies are of cross-sectional in nature; however, the study by Ek et al. that you cite analysed associations over a longer follow-up period. Similarly, the study by Jacob et al. assessed anxiety across two waves. I also believe, that they are quite a few studies which analyse trajectories. In the context of your rationale, I would suggest clarifying this sentence. You could mention that many studies assess trajectories either after a fall or in heterogeneous populations that include individuals with and without a history of falls, and that there appears to be a lack of studies examining the impact of a fall on changes in ongoing trajectories.

**Response:** Thank you for this comment and helpful suggestion. We agree that the original wording could have been misleading, as some of the cited studies used longitudinal designs. Our intention was to highlight that many previous studies relied on single assessments of exposure and/or outcome, rather than to imply that they were cross-sectional. We have therefore revised the wording to avoid this ambiguity.

We also agree with you that some studies have assessed trajectories either after a fall or in heterogeneous populations that include individuals with and without a history of falls. We appreciate your suggested clarification, and we placed the revised statement at the end of the paragraph because it better connects the existing evidence with the following paragraph, which explains the rationale for the present study.

(Page 6, Lines 94-96) Much of the existing research has examined activities of daily living (ADL), depressive symptoms, and cognitive impairment either as risk factors for falls or as consequences of falls, often using longitudinal time-to-event analyses.

(Page 6, Lines 101-102) Emerging studies have also assessed functional trajectories either after a fall or in heterogeneous populations that include individuals with and without a history of falls.

**Comment 7:** 88-90 I would split this sentence into two separate ones to improve readability and precision. In the second sentence, I would suggest explicitly mentioning the comparison group (e.g., individuals without a history of falls). For example: "Falls were associated with poorer ADLs compared to individuals without fall experience."

**Response:** We have revised the relevant text accordingly.

(Page 6, Lines 98-99) Conversely, in a study of 9,816 older Korean adults, individuals with a history of falls had poorer activities of daily living (ADL) and cognitive performance than those without a history of falls.

**Comment 8:** 92 This association is also longitudinal. How does this reference strengthen your rationale?

**Response:** Thank you for raising this question. Similar to the studies by Ek et al. and Lee et al., the study by Choi et al. was cited to support the bidirectional associations between falls and impairments in physical disability, depressive symptoms, and cognitive impairment. However, these studies largely remain within a time-to-event or pairwise association framework and therefore do not fully capture how falls are associated with changes in ongoing functional trajectories. As we note in the following paragraph, examining the impact of a fall on changes in ongoing trajectories may help identify opportunities for timely multifactorial fall prevention and inform post-fall management.

**Comment 9:** 93 The rationale represents an important research aim. You could further strengthen the introduction by highlighting that trajectories following a fall are relatively well studied, whereas comparisons of trajectories before and after a fall remain limited. Is there literature examining whether more severe falls are associated with changes in these trajectories?

**Response:** Thank you for this constructive suggestion. We have revised the Introduction to better highlight this important research aim. As you also noted in your previous comment, we acknowledge that some studies have examined functional trajectories either after a fall or in heterogeneous populations that include individuals with and without a history of falls.

We reviewed the relevant literature and found that some previous studies of post-fall functional decline have considered fall severity, for example by distinguishing injurious falls and hip fractures. The study by Gill et al. is relevant to our work, as it examined trajectories of physical disability before serious fall injuries and the likelihood of functional recovery afterward. We have discussed how its findings compare with ours in the Discussion. In addition, the study by Kou et al., cited above, examined trajectories of cognitive function after falls and accounted for fall frequency.

(Page 6, Lines 101-102) Emerging studies have also assessed functional trajectories either after a fall or in heterogeneous populations that include individuals with and without a history of falls.

However, few studies have examined physical, psychological, and cognitive functioning in relation to falls to describe how these key domains evolve in the periods before and after fall events, while comprehensively considering fall severity and related characteristics.

**Comment 10:** 96 The "s" is missing in "hip fracture" to indicate the plural.

**Response:** We have revised it.

**Comment 11:** 109 no hyphen between individual and participant.

**Response:** We have revised it.

**Comment 12:** 110 Were these countries chosen because their data are most comparable? Are there differences in characteristics between these countries, for example in fall incidence?

**Response:** Thank you for this question. Comparability was an important reason for selecting these cohorts. After considering all HRS-family cohorts, we selected CHARLS, ELSA, and HRS because they provide **more than 10 years of follow-up, have comparable assessments of falls and fall-related characteristics, and include harmonizable measures of ADL disability, depressive symptoms, and cognitive function**. Although other HRS-family studies, such as SHARE, also have long-term follow-up, their measurements of falls and functional domain were less consistent with those in CHARLS, ELSA, and HRS, and therefore were not included in the present harmonized analysis.

We acknowledge that fall occurrence differed across cohorts. Both our raw data and previous burden studies suggested that fall occurrence was slightly higher in the US than in England and China.

(Page 7, Lines 123-126) These cohorts were selected because, among all HRS-family cohorts, they provided more than 10 years of follow-up, comparable fall-related assessments, and harmonizable measures of physical disability, depressive symptoms, and cognitive functioning.

**Comment 13:** 120 To make the data more comparable, would it not have been possible to include all persons aged 65 years? Why did you decide against that?

**Response:** Thank you for raising this point. First, we included participants aged ≥60 years in ELSA and CHARLS to minimize sample loss as much as possible, especially for the hip fracture subgroup. In addition, based on WHO guidelines, age 60 years is already a relevant threshold for fall-risk stratification. We also considered comparability and noted in the Discussion the need for cautious interpretation of cohort-specific results.

**Comment 14:** Could you clarify why fall severity was stratified using these variables? Was this decision based on the availability of data for these factors? The World Falls Guidelines, for example, also suggest categories such as frailty and loss of consciousness after a fall.

**Response:** We acknowledge that the fall severity indicators we used were selected primarily based on data availability and represent all fall-related variables available in the questionnaires. We agree that the World Falls Guidelines suggest additional indicators, such as frailty and loss of consciousness, for assessing fall severity; however, these measures were not available in the cohorts we used. For example, even frailty is typically calculated using over 30 component variables to construct a frailty index in these cohorts, many of which overlap with items assessing ADL, depressive symptoms, and cognitive functioning. We have acknowledged this limitation.

(Page 22, Lines 432-434) Seventh, data unavailability limited our ability to assess additional fall-severity indicators suggested by the World Falls Guidelines, such as frailty and loss of consciousness after a fall.

**Comment 15:** 154 I wonder whether there is an even better term than "psychological functioning", because cognitive function could also be considered part of psychological functioning, and because depression represents only a small part of psychological functioning.

**Response:** We agree with your comment and consider “depressive symptoms” to be a more appropriate term. We have revised the terminology where appropriate throughout the manuscript.

**Comment 16:** 178 Was there information on other conditions such as glaucoma, Parkinson's disease?

**Response:** Yes, related information was available to some extent. For Parkinson’s disease, the harmonized variable consistently available across the three cohorts was “whether participants had ever had a memory-related disease, such as Alzheimer’s disease, dementia, brain atrophy, or Parkinson’s disease”. Glaucoma was not directly assessed in all three cohorts; therefore, we used self-rated eyesight as a proxy variable. **These two harmonized variables were additionally adjusted for in all models**, and their details are provided in **Table S1**.

(Page 11, Lines191-200) Covariates were drawn from the baseline wave and included sociodemographic characteristics (age, sex, education level [primary, secondary, tertiary], net household wealth [Q1–Q4], and marital status [married or partnered; widowed; separate or divorced or single]), health-related lifestyle factors (current smoking status, alcohol consumption, and physical activity), eyesight, hearing, and chronic conditions (hypertension, diabetes, chronic lung disease, heart problem, stroke, arthritis, cancer, and memory-related diseases). Given their particular relevance to falls, stroke, arthritis, and memory-related disease were included as separate covariates, while other chronic conditions were summarized as the number of comorbidities (0, 1, ≥2). Missing values for covariates were categorized as “unknown”. Harmonized covariate classifications are shown in the appendix **(Table S1 in S1 Appendix)**.

**Comment 17:** 193 and following: fall-related conditions: what do you define as a fall-related conditions? I would suggest defining this better in the methods section.

**Response:** Thank you for this suggestion. We have revised the relevant wording in the definition and avoided using the term “fall-related conditions.” As you also suggested in a subsequent comment, we have made corresponding revisions in other relevant parts of the manuscript.

(Page 9, Lines 154-155) To assess fall severity and fall-related characteristics, we constructed three alternative indicators: falls with injuries, recurrent falls, and falls with hip fracture.

**Comment 18:** 200 why was the cut-off 7 years and not a shorter time period?

**Response:** Thank you for raising this point. In the subgroup of falls with hip fracture, periods with fewer than 20 participants were not included in model fitting to avoid unstable estimates, even though time was modeled as a continuous variable. Considering the follow-up structure, approximately 7 years before the fall corresponded to the earliest available wave with sparse observations in this subgroup; therefore, we used 7 years (n = 17) as the cut-off rather than applying an exact cut-off based solely on n = 20. This is also shown in **Figure 2** and **Table S9**.

**Comment 19:** 201 The entire sentence is bold, although only the headline should be.

**Response:** We have corrected the formatting, and only the subheading “**Subgroup and sensitivity analyses:**” is now bolded.

**Comment 20:** 229 I would suggest stating the origin of each database earlier. It may not be common knowledge that, for example, the Health and Retirement Study is a USA-based study.

**Response:** We have revised the Methods section to state the origin of each cohort.

(Page 7, Lines 120-123) This population-based, cross-national study used individual participant data from community-based samples in three sister cohorts in the Global Aging, Health, and Policy programme: the China Health and Retirement Longitudinal Study (CHARLS; China), the English Longitudinal Study of Ageing (ELSA; England), and the Health and Retirement Study (HRS; United States).

**Comment 21:** 250 I would suggest rewording this section. Recurrent falls should not be classified as a fall-related condition, and hip fractures are more appropriately described as injuries rather than conditions.

**Response:** We have revised the relevant wording throughout the manuscript. Please also see our response to your **Comment 17**. In addition, we have also revised this paragraph to improve readability.

**Comment 22:** Figure 2: Very interesting!

**Response:** Thank you!

**Comment 23:** 314 Please see my comment on the introduction. The point you are making is strong; however, I would suggest a different argument, as there are many longitudinal studies, which you then mention a couple sentences later.

**Response:** Thanks for this suggestion. We have revised accordingly.

(Page 18, Lines 346-348) The present findings extend previous evidence on bidirectional associations between falls and ADL disability, depressive symptoms, and cognitive performance, which has largely relied on longitudinal time-to-event analyses reflecting between-person differences.

**Comment 24:** 316 I would suggest placing the phrase "highlights a long window for prevention and intervention" at the end of the paragraph. This represents an important implication of the findings for future research and could be elaborated further. For example, individuals with less favourable trajectories may have more risk factors, suggesting that prevention requires sustained efforts. Interventions such as strength and balance training need to be done regularly. At the same time, the importance of secondary prevention could be emphasised to reduce the risk of subsequent falls.

**Response:** Thank you for this helpful suggestion. We have placed it at the end of the paragraph and elaborated on it.

(Page 18, Lines 356-360) In addition, individuals with less favorable trajectories may have more risk factors[11], underscoring the need for sustained primary prevention, such as regular mobility (e.g., balance, gait, and strength) and cognitive training[6,13], as well as secondary prevention to reduce the risk of subsequent falls[2,28]. Together, this highlights a long window for prevention and intervention.

**Comment 25:** 338 It is possible that interventions targeting physical function may also benefit other domains. For example, physical activity may positively influence cognitive health, while group-based exercise and social interaction may contribute to overall well-being.

**Response:** We agree with this suggestion and have revised the wording to acknowledge these potential cross-domain benefits.

(Pages 19-20, Lines 372-386) Interventions targeting physical functioning for older adults with lived fall experience may also have benefits for depressive symptoms and cognitive health or broader well-being[28,31]. Nevertheless, interventional research is needed to test whether strategies targeting multiple functional domains outperform those focused on physical functioning only.

**Comment 26:** 341 I would suggest using the term "concerns about falling" instead of "fear of falling", because it may be perceived as less stigmatizing.

**Response:** We have revised it.

**Comment 27:** 347 Perhaps you could place the following paragraph on implications before the future research paragraph. This would allow the future research paragraph to build on the implications.

**Response:** Thank you for this helpful suggestion. We have moved the paragraph on public-health implications one paragraph earlier, before the paragraph on future mechanistic research. We did not move it before the preceding two paragraphs because those paragraphs mainly discuss the multi-trajectory findings and their research implications. Therefore, we believe that moving the paragraph forward by one position is the most appropriate.

**Comment 28:** 348 How do you define simple assessments? Assessments that are short and quick? Maybe it would also be relevant that the screenings are regular to identify decline earlier? Can you define a cut-off of, based on this research, when decline is relevant and significant?

**Response:** We agree with you that a “simple assessment” should be short, quick, and feasible to implement in routine settings. We have revised the text and have provided examples of simple assessment tools suggested by the World Fall Guidelines. We have also emphasized the importance of regular screening to facilitate earlier detection of functional decline. However, we acknowledge that defining a specific cut-off for clinically relevant decline is challenging based on the present study; therefore, we have interpreted this point cautiously in the revised text.

(Page 20, Lines 387-395) Short and quick assessments of physical, psychological, and cognitive functioning—such as brief questionnaires, short physical performance battery or balance tests, and rapid cognitive screening tools—may help identify older adults at higher risk of falls in older adults[2,4,6], and when decline in these functional domains is identified, individualized support tailored to functional needs may be warranted to help reduce falls and their more severe consequences[2,6]. Regular screening that tracks functional changes may facilitate early detection of decline. For example, our multi-trajectory modelling suggests that even modest deterioration, approximately 0.25-SD relative to the baseline distribution, may be associated with higher odds of falls.

**Comment 29:** 351 Not all older adults have a caregiver, and individuals are often responsible for managing their own health. It may therefore be more appropriate to refer to the individual and their support system, as well as the healthcare system.

**Response:** Thank you for this helpful suggestion. We have revised the text to further acknowledge the role of older adults themselves in post-fall assessment, rehabilitation, and self-management, as well as the support provided by their support systems and the healthcare system.

(Page 20, Lines 395-398) Once a fall occurs, older adults’ support systems and the healthcare system should provide comprehensive support to promote recovery across these domains and prevent further decline, while older adults themselves should be encouraged to actively engage in post-fall assessment, rehabilitation, and self-management as well[2].

**Comment 30:** 364 This may also be relevant for falls. If a fall occurred a long time ago and had no severe consequences, it may not be recalled or reported. It appears that fall status prior to cohort entry was not assessed and information on that is not available, right? If so, the fall included in the analysis may not represent the first fall, and earlier falls may have already contributed to functional decline?

**Response:** Thank you for raising this important point. We agree that this issue needs to be clarified. We have added this as a limitation and clarified its potential influence on the interpretation of our findings.

(Page 21, Lines 418-422) Second, fall history may be affected by recall bias, particularly when a fall occurred a long time ago and had no severe consequences. In addition, falls before cohort entry could not be fully assessed. Therefore, some participants with earlier falls may have been misclassified into the no-fall group, which may have led to underestimation of the observed associations.

**Comment 31:** Would it be a limitation that slightly different questionnaires were used, for example for assessing depression?

**Response:** We agree with you that the use of slightly different questionnaires across cohorts should be acknowledged as a limitation.

(Pages 21-22, Lines 427-431) Fifth, between-cohort differences (e.g., mean age and the instruments used to assess functioning) should be taken into account. Therefore, cohort-specific interpretations may still be more appropriate in certain contexts. In addition, although z-scores are used to harmonize such heterogeneity, some valuable original information may have been lost.

**Comment 32:** 370 What hypotheses do you have regarding the surgery data, particularly given its potential impact on fall severity?

**Response:** Thank you for this thoughtful question. Our hypothesis is that surgery may play two roles in the interpretation of fall severity and post-fall recovery. First, the need for surgery, particularly after hip fracture, is likely to indicate more severe fall-related injury and may therefore be associated with poorer functional status around the fall event. Second, surgical treatment and subsequent rehabilitation may also influence post-fall trajectories by relieving pain, restoring mobility, and facilitating physical recovery; they may also indirectly affect depressive symptoms or broader well-being through improved independence and activity. Because surgery data were not available, we could not examine whether surgical management modified post-fall functional trajectories or distinguish the contribution of injury severity from that of treatment and rehabilitation.

Dyer SM, Crotty M, Fairhall N, et al. A critical review of the long-term disability outcomes following hip fracture. BMC Geriatr. 2016;16:158. doi:10.1186/s12877-016-0332-0.

Step Safely: Strategies for Preventing and Managing Falls Across the Life-Course. 1st ed. Geneva: World Health Organization; 2021.

**Comment 33:** 375 Very important result!

**Response:** Thank you :)

**Comment 34:** 378 I wonder whether the conclusion should instead be that risk assessment should be multifactorial, as also stated in the World Falls Guidelines.

**Response:** Thank you for this suggestion. We have revised the conclusion paragraph accordingly.

(Page 22, Lines 438-441) In summary, falls at older ages are often associated with long-term multidomain functional deterioration, particularly in relation to more severe fall events. These findings support multifactorial risk assessment, prevention, and post-fall management strategies that comprehensively consider physical, psychological, and cognitive domains, rather than focusing solely on physical functioning.

**Reviewer #2**

Firstly, I would like to commend the authors on their recent submission which is indeed thorough. They show clear evidence of statistical considerations with subgroup and sensitivity analyses. The question posed is of importance, assessing falls and its impact on physical, psychological and cognitive functioning. I have some points below for your consideration. Please note, I recommended major revisions due to the potentially long computational time for modelling.

**Response:** We sincerely thank you for your thorough evaluation and constructive comments.

**Comment 1:** The methods should mention that all patients are community dwelling, it is reported as a limitation but I feel it should be clearly stated earlier.

**Response:** Thank you for this suggestion. We have clarified this point in the Methods section.

(Page 7, Lines 120-123) This population-based, cross-national study used individual participant data **from community-based samples** in three sister cohorts in the Global Aging, Health, and Policy programme: the China Health and Retirement Longitudinal Study (CHARLS; China), the English Longitudinal Study of Ageing (ELSA; England), and the Health and Retirement Study (HRS; United States).

**Comment 2:** Age was not considered during the matching process. In table S2 it shows the HRS cohort is older than the other two (74.9 vs 66.8 and 69.7 years). This also leads to a statistically significant difference in the matching for age. With those who fall being older, this is commonly known impact of ageing. I appreciate this is then utilised as a covariate in the model. Could the authors please clarify their reasoning for this? Do they consider age to be a predictive factor, similar to number of comorbidities, and have included due to this? If so, could the authors please clarify why they chose the factors they did for matching and those they did not. Additionally, as age is a significant factor for falls, were there more falls in the HRS cohort than the other cohorts? Number of falls data is not presented but is an outcome. I appreciate multiple falls is not available for one cohort (CHARLS).

**Response:** Thank you for raising this important point. We apologize that our original wording may not have made the matching procedure sufficiently clear. Age was considered in the matching process; specifically, we performed 1:1 matching between participants with and without falls based on age (±2 years) and sex within each cohort. We have revised the Methods section to clarify this point.

We selected age, sex, and cohort for matching because these are key demographic and design-related factors. Matching on these variables helped avoid substantial loss of eligible participants, particularly in small subgroups with severe fall-related outcomes, which could still have occurred even with propensity score matching. We were also cautious about potential overmatching and reduced statistical efficiency.

We have additionally included descriptive statistics for falls and fall-related characteristics across the different cohorts in **Table S2** to provide further clarity. In addition, we have noted the between-cohort differences in age in the Limitations section and emphasized the importance of cautious interpretation of cohort-specific results.

(Page 8, Lines 148-149) Then, we performed 1:1 matching between participants who reported falls during follow-up and those who did not based on age (±2 years) and sex within each cohort.

(Pages 20-21, Lines 427-432) Fifth, between-cohort differences (e.g., mean age and the instruments used to assess functioning) should be taken into account. Therefore, cohort-specific interpretations may still be more appropriate in certain contexts. In addition, although z-scores are used to harmonize such heterogeneity, some valuable original information may have been lost. Sixth, despite matching and adjustment for some key covariates, residual confounding may remain.

**Comment 3:** With regards to comorbidities, was data available for those who had neurological conditions such as Parkinson disease? If so, can the authors provide justification for not considering those patients? Neurological conditions do not appear to be part of the eligibility criteria.

**Response:** Thank you for raising this important point. We agree with you that neurological conditions are relevant to falls and functional decline and should be considered. Rather than excluding participants with such conditions, we retained them to better reflect the real-world population of community-dwelling older adults and have now additionally adjusted. The harmonized variable consistently available across the three cohorts was whether participants had ever had a memory-related disease, such as Alzheimer’s disease, dementia, brain atrophy, or Parkinson’s disease. As also suggested by Reviewer #1, we further adjusted for eyesight and hearing in all models.

**Reviewer #1, Comment 16:** 178 Was there information on other conditions such as glaucoma, Parkinson's disease?

**Response to Reviewer #1, Comment 16:** Yes, related information was available to some extent. For Parkinson’s disease, the harmonized variable consistently available across the three cohorts was “whether participants had ever had a memory-related disease, such as Alzheimer’s disease, dementia, brain atrophy, or Parkinson’s disease”. Glaucoma was not directly assessed in all three cohorts; therefore, we used self-rated eyesight as a proxy variable. **These two harmonized variables were additionally adjusted for in all models**, and their details are provided in **Table S1**.

**Comment 4:** For the analyses of separate trajectories, linear mixed effect models are utilised, however, the figures suggest that linearity does not hold. I would recommend a sensitivity analysis using non-linear methods, the piecewise model could be applied in a non-linear manner. You may also wish to consider other models such as fractional polynomials or spline models, if not, provide justification.

**Response:** Thank you for this helpful suggestion and for providing several possible approaches to address potential non-linearity. We conducted an additional sensitivity analysis using spline-based mixed-effects models with natural cubic splines. This approach (splines model) allowed us to flexibly model potential non-linear trajectories without imposing a specific parametric functional form (nlme model). The results were generally consistent with the main findings, suggesting that our conclusions were robust to potential departures from linearity (Table S20, Figure S5).

**Table S20.** Sensitivity analysis 3: Conducting spline-based mixed-effects models with natural cubic splines of 2 degrees of freedom

|  | **Physical functioning β (95% CI)** | **Psychological functioning β (95% CI)** | **Cognitive functioning β (95% CI)** |
| --- | --- | --- | --- |
| **Fall status ^a^** |  |  |  |
| Falls *(n = 5,495)* | **-0.293 (-0.342, -0.244)** | **-0.234 (-0.271, -0.197)** | **-0.044 (-0.080, -0.007)** |
| Falls × pre_spline1 | -0.079 (-0.342, -0.185) | -0.009 (-0.205, 0.186) | -0.013 (-0.197, 0.171) |
| Falls × pre_spline2 | **-0.362 (-0.428, -0.295)** | **-0.124 (-0.174, -0.073)** | **-0.056 (-0.103, -0.009)** |
| Falls × post_spline1 | **-0.286 (-0.388, -0.184)** | 0.254 (-0.052, 0.103) | -0.027 (-0.100, 0.045) |
| Falls × post_spline2 | **-0.250 (-0.355, -0.144)** | -0.020 (-0.103, 0.062) | **-0.108 (-0.192, -0.024)** |
| **Injuries from falls ^a^** |  |  |  |
| Falls without injuries *(n = 3,131)* | **-0.220 (-0.275, -0.134)** | **-0.188 (-0.231, -0.145)** | **-0.049 (-0.092, -0.007)** |
| Falls with injuries *(n = 1,795)* | **-0.452 (-0.520, -0.385)** | **-0.291 (-0.343, -0.239)** | **-0.063 (-0.115, -0.011)** |
| Falls without injuries × pre_spline1 | -0.178 (-0.467, 0.112) | 0.022 (-0.196, 0.241) | 0.035 (-0.173, 0.243) |
| Falls with injuries × pre_spline1 | 0.020 (-0.338, 0.378) | -0.044 (-0.316, 0.227) | -0.198 (-0.459, 0.064) |
| Falls without injuries × pre_spline2 | **-0.255 (-0.329, -0.180)** | **-0.081 (-0.137, -0.024)** | -0.045 (-0.099, 0.008) |
| Falls with injuries × pre_spline2 | **-0.577 (-0.668, -0.486)** | **-0.193 (-0.263, -0.122)** | **-0.086 (-0.154, -0.019)** |
| Falls without injuries × post_spline1 | **-0.227 (-0.343, -0.110)** | 0.070 (-0.020, 0.159) | 0.039 (-0.046, 0.123) |
| Falls with injuries × post_spline1 | -0.054 (-0.192, 0.085) | 0.024 (-0.085, 0.132) | -0.094 (-0.198, 0.010) |
| Falls without injuries × post_spline2 | -0.091 (-0.218, 0.034) | -0.009 (-0.109, 0.091) | -0.056 (-0.156, 0.044) |
| Falls with injuries × post_spline2 | **-0.230 (-0.371, -0.089)** | -0.024 (-0.139, 0.090) | **-0.146 (-0.268, -0.024)** |
| **Recurrent falls ^b^** |  |  |  |
| Single fall *(n = 1,901)* | -0.059 (-0.126, 0.008) | **-0.102 (-0.153, -0.050)** | -0.003 (-0.052, 0.048) |
| Multiple falls *(n = 1,516)* | **-0.516 (-0.589, -0.443)** | **-0.326 (-0.383, -0.270)** | **-0.143 (-0.198, -0.088)** |
| Single fall × pre_spline1 | -0.006 (-0.381, 0.369) | 0.070 (-0.223, 0.363) | 0.118 (-0.149, -0.385) |
| Multiple falls × pre_spline1 | 0.252 (-0.204, 0.709) | 0.023 (-0.335, 0.381) | 0.013 (-0.312, 0.337) |
| Single fall × pre_spline2 | -0.088 (-0.177, 0.001) | -0.035 (-0.105, 0.035) | -0.055 (-0.119, 0.009) |
| Multiple falls × pre_spline2 | **-0.646 (-0.750, -0.542)** | **-0.173 (-0.256, -0.091)** | **-0.128 (-0.203, -0.053)** |
| Single fall × post_spline1 | -0.056 (-0.193, 0.081) | -0.009 (-0.117, 0.100) | -0.002 (-0.101, 0.097) |
| Multiple falls ×post _spline1 | **-0.411 (-0.558, -0.264)** | 0.062 (-0.056, 0.180) | 0.002 (-0.106, 0.109) |
| Single fall × post_spline2 | -0.076 (-0.239, 0.088) | -0.030 (-0.160, 0.101) | -0.087 (-0.207, 0.033) |
| Multiple falls × post_spline2 | **-0.233 (-0.408, -0.057)** | -0.070 (-0.212, 0.072) | -0.065 (-0.198, 0.068) |
| **Falls with hip fracture ^c^** |  |  |  |
| Falls without hip fracture *(n = 5,120)* | **-0.270 (-0.318, 0.221)** | **-0.232 (-0.370, -0.194)** | **-0.041 (-0.078, -0.003)** |
| Falls with hip fracture *(n = 135)* | **-1.522 (-1.736, -1.308)** | **-0.450 (-0.624, -0.277)** | **-0.241 (-0.413, -0.069)** |
| Falls without hip fracture × pre_spline1 | -0.088 (-0.350, -0.173) | 0.015 (-0.184, 0.214) | -0.040 (-0.228, 0.147) |
| Falls with hip fracture × pre_spline1 | 0.088 (-0.786, 2.537) | -0.710 (-2.003, -0.574) | -0.028 (-1.328, 1.272) |
| Falls without hip fracture × pre_spline2 | **-0.328 (-0.394, -0.261)** | **-0.120 (-0.171, -0.069)** | **-0.059 (-0.108, -0.011)** |
| Falls with hip fracture × pre_spline2 | **-1.747 (-2.060, -1.434)** | **-0.407 (-0.658, -0.156)** | -0.148 (-0.375, 0.098) |
| Falls without hip fracture × post_spline1 | **-0.234 (-0.334, -0.133)** | 0.016 (-0.063, 0.094) | -0.021 (-0.095, 0.053) |
| Falls with hip fracture × post_spline1 | 0.328 (-0.148, 0.805) | **0.612 (0.214, 1.010)** | -0.227 (-0.614, 0.160) |
| Falls without hip fracture × post_spline2 | **-0.207 (-0.312, -0.102)** | -0.020 (-0.103, 0.064) | **-0.099 (-0.184, -0.014)** |
| Falls with hip fracture × post_spline2 | -0.322 (-0.905, 0.261) | **0.064 (0.110, 1.163)** | -0.190 (-0.731, 0.351) |

Models were adjusted age, sex, cohort, education level, total household wealth, marital status, current smoking status, current alcohol consumption, physical activity, stroke, arthritis, memory-related diseases, eyesight, hearing, and number of other comorbidities;

**Bolded text indicates statistically significant;**

**Note:** pre/post_spline represents a centered natural cubic spline basis term for time before/after matching wave; Spline terms were used to flexibly model potential non-linearity in functional trajectories; individual spline coefficients are not directly interpreted. Pre_fall internal knot: time = -2.25; and post_fall internal knot: time = 2.92;

a. Available for CHARLS, ELSA, and HRS, Ref: No falls (n = 5,495);

b. Available for ELSA, and HRS, Ref: No falls (n = 3,976);

c. Available for CHARLS, ELSA, and HRS, Ref: No falls (n = 5,371).

**Figure S5.** Trajectories of physical, psychological, and cognitive functioning before and after falls and fall-related characterizations using spline-based mixed-effects models.

Models were adjusted age, sex, cohort, education level, total household wealth, marital status, current smoking status, current alcohol consumption, physical activity, stroke, arthritis, memory-related diseases, eyesight, hearing, and number of other comorbidities;

Pre_fall internal knot: time = -2.25; and post_fall internal knot: time = 2.92.

**Reviewer #3**

The manuscript addresses an important and timely topic concerning changes in physical, psychological, and cognitive functioning before and after falls in older adults. A major strength of the study is the use of data from three large longitudinal cohorts and the multidomain approach to functional trajectories. The findings suggesting that functional decline begins several years before a fall and continues afterward are interesting and may have important clinical and public health implications. However, several issues should be addressed before publication.

**Response:** We thank you for taking the time to review our manuscript and for providing these helpful comments. Point-by-point replies to the comments are provided below.

**Comment 1:** The interpretation of the findings is sometimes too strong. Since this is an observational study, it is difficult to determine whether declines in functioning are a consequence of falls or whether poorer functioning increases the risk of falling. The conclusions should therefore be more cautious.

**Response:** Thank you for this important comment. We agree with you that, given the observational design, the results should be interpreted as associations rather than evidence of causality. We have revised the relevant text to use more cautious wording.

e.g., (Page 17, Lines 339-340) Although a more marked decline in cognitive functioning was observed only after the fall

(Page 17, Lines 344-345) Finally, less favorable multidomain functional trajectories were most commonly observed among participants who experienced more severe falls.

(Pages 18-19, Lines 367-369) These findings suggest that personal and social resources in older adults may be related to the risk and recovery of severe fall-related outcomes.

(Page 19, Lines 381-382) These findings support the use of multifactorial approaches in fall prevention and post-fall management.

(Page 20, Lines 389-390) may help identify older adults at higher risk of falls

**Comment 2:** Falls were assessed using self-reported data, which may introduce recall bias and underreporting. This limitation should be discussed in more detail.

**Response:** We have expanded the discussion of this limitation in the revised manuscript.

(Page 21, Lines 418-422) Second, fall history may be affected by recall bias, particularly when a fall occurred a long time ago and had no severe consequences. In addition, falls before cohort entry could not be fully assessed. Therefore, some participants with earlier falls may have been misclassified into the no-fall group, which may have led to underestimation of the observed associations.

**Comment 3:** Matching was performed only for age, sex, and cohort. Despite matching, differences between groups remained in physical activity and comorbidities, suggesting possible residual confounding.

**Response:** Thank you for this helpful comment. We agree that residual confounding is possible. We have acknowledged this issue as a limitation.

(Page 21, Lines 431-432) Sixth, despite matching and adjustment for some key covariates, residual confounding may remain.

**Comment 4:** Although harmonization across CHARLS, ELSA, and HRS is an important strength, differences in measurements and assessment tools between cohorts may still affect comparability of the results. This issue deserves further discussion.

**Response:** We have acknowledged this limitation and underscored the importance of interpreting cohort-specific results.

(Pages 20-21, Lines 427-431) Fifth, between-cohort differences (e.g., mean age and the instruments used to assess functioning) should be taken into account. Therefore, cohort-specific interpretations may still be more appropriate in certain contexts. In addition, although z-scores are used to harmonize such heterogeneity, some valuable original information may have been lost.

**Comment 5:** Some observed associations, particularly those related to cognitive functioning, appear relatively small. It would be helpful to discuss whether these differences are clinically meaningful.

**Response:** Thank you very much for this helpful suggestion. After carefully reviewing the relevant literature and using the additional spline-based mixed-effects models **(Table S20 and Figure S5)**, we found that ADL disability and depressive symptoms may be more closely related to falls and fall-related injuries, whereas cognitive decline usually develops more gradually and may be influenced by longer-term or indirect pathways (Page xx, Lines xx-xx).

In the short term, although the smaller standardized differences in cognitive function may not represent clinically meaningful impairment at the individual level, the joint trajectory results suggest that they may still be relevant for population-level risk stratification, particularly when cognitive decline co-occurs with ADL disability and depressive symptoms. Therefore, interventions targeting physical function and depressive symptoms in older adults with lived fall experience may also have benefits for cognitive health. (Page xx, Lines xx-xx).

(Page 19, Lines 370-377) Second, the associations with cognitive function were smaller than those for ADL disability and depressive symptoms and should therefore be interpreted cautiously. Our spline-based mixed-effects models suggested that post-fall cognitive decline may be delayed relative to changes in physical function and depressive symptoms, raising a hypothesis that physical and psychological functioning may partly link falls with cognitive decline. A Swedish community-based study of 2,267 community-dwelling older adults showed that physical performance and depressive symptoms mediated approximately 26% and 8% of this association, respectively[33]. Further studies are needed to clarify the temporal interplay among these functional trajectories before and after falls.

(Pages 19-20, Lines 382-386) Interventions targeting physical functioning for older adults with lived fall experience may also have benefits for depressive symptoms and cognitive health or broader well-being[30,34]. Nevertheless, interventional research is needed to test whether strategies targeting multiple functional domains outperform those focused on physical functioning only.

**Comment 6:** Survivor bias may also have influenced the findings, especially among participants with severe falls or hip fractures.

**Response:** We have acknowledged this limitation, as also discussed above.

(Page 19, Lines 364-365) …… but, unexpectedly, some improvement in depressive symptoms after the fall. Whether survivor bias contributed to this pattern remains uncertain

(Page 21, Lines 423-425) Fourth, severe falls and hip fractures may be fatal in older adults; participants who died shortly after a fall or were lost to follow-up may introduce survivor bias, and this may also have attenuated the observed associations.

**Reviewer #4**

The authors investigated into the trajectories of matched fallers and non-fallers in the physical, psychological and cognitive function using data of three well known Longitudinal studies (UK, China, and USA). The trajectories of a longer period of time is making this manuscript very valuable, and will add further evidence to the current knowledge in the fall prevention area. The manuscript is already in good shape but some major comments needed to be addressed before it can be considered for publication. The reviewer has really enjoyed reading this manuscript.

**Response:** We sincerely thank you for your helpful comments and are glad that you enjoyed reading our manuscript.

**Comment 1:** Abstract: may be the authors should consider to also include the longitudinal aspect of these trajectories (which is an asset of this manuscript) into the background.

**Response:** We have revised it.

(Page 2, Lines 20-21) However, how these functional domains change longitudinally before and after fall events remains unclear.

**Comment 2:** Key words: For the reviewer the key word of "hip fracture" is not directly related but more important are information on the setting of the cohort.

**Response:** Thank you for this helpful suggestion. We have replaced “hip fracture” with “HRS-family cohorts” in the keywords.

**Comment 3:** Authors summary: well written no comments.

**Response:** Thank you!

**Comment 4:** The authors are leading very good into their research topic. They are citing the WHO Step safely and other important recommendations. Nevertheless, the reviewer would suggest to insert on page 6 line 78-85 also a quick introduction into the four domains provided by the WHO Framework "Step Safely".

**Response:** Thank you for this helpful suggestion. We have added a brief introduction of the four domains provided by the WHO Step Safely framework.

(Page 6, Lines 89-91) The World Health Organization Step Safely framework emphasizes that falls and fall-related injuries arise from interacting biological, behavioral, environmental, and socioeconomic risk factors.

**Comment 5:** In Line 86 it would be useful to insert an explanation of which variables in the physical psychological and cognitive domain the authors are taking into account. As for the reviewer the domain of cognition would normally be a subcategory of a psychological domain.

**Response:** We have revised it.

(Page 6, Lines 94-96) Much of the existing research has examined activities of daily living (ADL), depressive symptoms, and cognitive impairment either as risk factors for falls or as consequences of falls[5], often using longitudinal time-to-event analyses.

**Comment 6:** The objectives are very well stated at the end of the introduction.

**Response:** Thank you :)

**Comment 7:** For the reviewer the following statement it is not clear: "available data on physical, psychological, and cognitive functioning in at least one wave" (p 8 line 124-125), as the authors investigated also the pre-post trajectories and this would include at least two assessment times. Please explain.

**Response:** Thank you for raising this important point. Some participants provided functional data at only one wave, either at the fall/matching wave, before it, or after it (n = 774). Consistent with the use of piecewise mixed-effects models in longitudinal analyses, these participants could still be included because they contribute available information to the estimation of population-level trajectories, while also helping preserve sample size, particularly in small subgroups.

Two related sensitivity analyses supported the robustness of the results: (1) restricting the analysis to participants who had at least one measurement both before and after the fall/matching time point, thereby providing information on both pre-time and post-time; and (2) restricting the analysis to participants with at least three repeated measurements to improve the precision of trajectory estimation.

**Comment 8:** The classification and categorization process is well described.

**Response:** Thank you :)

**Comment 9:** As the authors categorized different faller groups (multiple faller, hip fracture faller) a short differentiation in line (p 13 line 226-236) should be added, as just the term "fallers" is being too broad. this aspect is later being shortly mentioned (same page line 239-240) but would be need in the short summary of results as stated above.

**Response:** Thank you for this suggestion. We agree that describing baseline characteristics across different fall subgroups is important. Accordingly, we have added baseline characteristics according to falls with injuries, recurrent falls, and falls with hip fracture in **Tables S3-S5**. Given space limitations, we did not provide a detailed interpretation of these supplementary tables in the main text; however, we have referred readers to these tables.

(Page 14, Lines 262-263) The characteristics of participants by fall-related characteristics are shown in the appendix (Tables S3-S5 in S1 Appendix).

**Comment 10:** The section p 14 line 250 to 266 is really hard to read, and authors should consider reviewing it or presenting it in a table for more readability.

**Response:** Thank you for this suggestion. We have revised the text to retain the key findings, with the detailed results presented in **Table 2**.

**Comment 11:** Already well written and in good shape.

**Response:** Thank you very much!

**Comment 12:** One aspect which the author might consider: the ADL measures are a very rough instrument of physical function and impairments in this area are probably more a sign of frailty (directing to a very vulnerable population), and might be therefore a bias of a predictor of falls.

**Response:** Thank you for this helpful comment. We agree that the ADL measures are a relatively crude instrument of physical function, and impairments in this domain may reflect broader frailty or vulnerability. Although frailty is typically calculated using more than 30 component variables in these cohorts (i.e., frailty index), many of these components overlap with items assessing ADL, depressive symptoms, and cognitive functioning, and therefore we did not include a separate frailty index as a comprehensive functional outcome. We have acknowledged this limitation and revised the terminology where appropriate throughout the manuscript to use “ADL disability” or “physical disability” when referring specifically to ADL-based measures, rather than broadly referring to “physical functioning.”

(Page 22, Lines 434-435) Eighth, the ADL measures used to assess physical functioning are relatively coarse and may primarily reflect underlying frailty.

**Comment 13:** Another aspect the authors might consider is that all the harmonization (z-scores, and categorization) valuable information may have been lost (just a short sentence acknowledge will be fine).

**Response:** We have acknowledged it.

(Page 22, Lines 430-431) In addition, although z-scores are used to harmonize such heterogeneity, some valuable original information may have been lost.

**General editorial requests:**

We ask every co-author listed on the manuscript to fill in a contributing author statement, making sure to declare all competing interests. If any of the co-authors have not filled in the statement, we will remind them to do so when the paper is revised. If all statements are not completed in a timely fashion this could hold up the re-review process. If new competing interests are declared later in the revision process, this may also hold up the submission. Should there be a problem getting one of your co-authors to fill in a statement we will be in contact. Please do not add or remove authors without first discussing this with the handling editor. You can see our competing interests policy here: http://journals.plos.org/plosmedicine/s/competing-interests.

**Response:** Thanks for the reminder.

Please upload any figures associated with your paper as individual TIF or EPS files with 300dpi resolution at resubmission; please read our figure guidelines for more information on our requirements: http://journals.plos.org/plosmedicine/s/figures. While revising your submission, we strongly recommend that you use PLOS's NAAS tool (https://ngplosjournals.pagemajik.ai/artanalysis) to test your figure files. NAAS can convert your figure files to the TIFF file type and meet basic requirements (such as print size, resolution), or provide you with a report on issues that do not meet our requirements and that NAAS cannot fix.

**Response:** All figures now meet the requirements.

Please ensure that the paper adheres to the PLOS Data Availability Policy (see http://journals.plos.org/plosmedicine/s/data-availability), which requires that all data underlying the study's findings be provided in a repository or as Supporting Information. For data residing with a third party, authors are required to provide instructions with contact information (web or email address) for obtaining the data. Please note that a study author cannot be the contact person for the data. PLOS journals do not allow statements supported by "data not shown" or "unpublished results." For such statements, authors must provide supporting data or cite public sources that include it.

**Response:** We have stated this in the **Data availability** section.

We expect all researchers with submissions to PLOS in which author-generated code underpins the findings in the manuscript to make all author-generated code available without restrictions upon publication of the work. In cases where code is central to the manuscript, we require the code to be made available as a condition of publication. Authors are responsible for ensuring that the code is reusable and well documented. Please make any custom code available, either as part of your data deposition or as a supplementary file. Please add a sentence to your data availability statement regarding any code used in the study, e.g. "The code used in the analysis is available from Github [URL] and archived in Zenodo [DOI link]" Please review our guidelines at https://journals.plos.org/plosmedicine/s/materials-software-and-code-sharing and ensure that your code is shared in a way that follows best practice and facilitates reproducibility and reuse. Because Github depositions can be readily changed or deleted, we encourage you to make a permanent DOI'd copy (e.g. in Zenodo) and provide the URL.

**Response:** We have stated how the code can be accessed in the Data Availability section, and the code will be made publicly available upon acceptance of the manuscript.

Abstract: Please structure your abstract using the PLOS Medicine headings (Background, Methods and Findings, Conclusions). Please combine the Methods and Findings sections into one section.

**Response:** We have revised it.

At this stage, we ask that you include a short, non-technical Author Summary of your research to make findings accessible to a wide audience that includes both scientists and non-scientists. The Author Summary should immediately follow the Abstract in your revised manuscript. This text is subject to editorial change and should be distinct from the scientific abstract. Ideally each sub-heading should contain 2-3 single sentence, concise bullet points containing the most salient points from your study. In the final bullet point of 'What Do These Findings Mean?', please include the main limitations of the study in non-technical language. Please see our author guidelines for more information: https://journals.plos.org/plosmedicine/s/revising-your-manuscript#loc-author-summary.

**Response:** We have added it.

Please express the main results with 95% CIs as well as p values. When reporting p values please report as p<0.001 and where higher as the exact p value p=0.002, for example. Throughout, suggest reporting statistical information as follows to improve clarity for the reader "22% (95% CI [13%,28%]; p</=)". Please be sure to define all numerical values at first use.

**Response:** Thanks for the reminder.

Please include page numbers and line numbers in the manuscript file. Use continuous line numbers (do not restart the numbering on each page).

**Response:** Thanks for the reminder.

Please cite the reference numbers in square brackets. Citations should precede punctuation.

**Response:** Thanks for the reminder.

Please provide titles and legends for all figures and tables (including those in Supporting Information files).

**Response:** Thanks for the reminder.

Please define all abbreviations used in each figure/table (including those in Supporting Information files).

**Response:** Thanks for the reminder.

Please consider avoiding the use of red and green in order to make your figure more accessible to those with color blindness.

**Response:** Not applicable.

Please note that supplementary material will be posted as supplied by the authors. Therefore, please amend it according to the relevant comments outlined here.

**Response:** Thanks for the reminder.

Please cite your Supporting Information as outlined here: https://journals.plos.org/plosmedicine/s/supporting-information

**Response:** We have revised relevant texts according to this guideline.

PLOS uses the numbered citation (citation-sequence) method and first six authors, et al.

**Response:** We have revised them.

Please ensure that journal name abbreviations match those found in the National Center for Biotechnology Information (NCBI) databases (http://www.ncbi.nlm.nih.gov/nlmcatalog/journals), and are appropriately formatted and capitalised.

**Response:** Thanks for the reminder.

Where website addresses are cited, please include the complete URL and specify the date of access (e.g. [accessed: 12/06/2023]).

**Response:** Thanks for the reminder.

Please also see https://journals.plos.org/plosmedicine/s/submission-guidelines#loc-references for further details on reference formatting.

**Response:** Thanks.

Abstract: Please include the study design, population and setting, number of participants, years during which the study took place (enrollment and follow up), length of follow up, and main outcome measures.

**Response:** We have revised the abstract.

Please ensure that the study is reported according to the STROBE (or appropriate STOBE extension) guideline (available from: https://www.equator-network.org/reporting-guidelines/strobe) and include the completed STROBE (or STROBE extension) checklist as Supporting Information. Please add the following statement, or similar, to the Methods: "This study is reported as per the Strengthening the Reporting of Observational Studies in Epidemiology (STROBE) guideline (S1 Checklist)." When completing the checklist, please use section and paragraph numbers, rather than page numbers.

**Response:** We have uploaded the STROBE checklist and referred to it in the Methods section.

[FOR MEDIATION ANALYSES] We recommend that the study is reported according to the AGReMA statement (https://agrema-statement.org/#:~:text=AGReMA%20is%20an%20evidence%2D%20and,randomised%20trials%20and%20observational%20studies) and include the completed checklist as Supporting Information. Please add the following statement, or similar, to the Methods: "This study is reported as per the Guideline for Reporting Mediation Analyses (AGReMA) statement (S1 Checklist)." When completing the checklist, please use section and paragraph numbers, rather than page numbers.

**Response:** Not applicable.

For all observational studies, in the manuscript text, please indicate: (1) the specific hypotheses you intended to test, (2) the analytical methods by which you planned to test them, (3) the analyses you actually performed, and (4) when reported analyses differ from those that were planned, transparent explanations for differences that affect the reliability of the study's results. If a reported analysis was performed based on an interesting but unanticipated pattern in the data, please be clear that the analysis was data driven.

**Response:** We have revised the relevant sections of the manuscript accordingly.

Please state in the Methods section whether the study had a prospective protocol or analysis plan. If a prospective analysis plan (from your funding proposal, IRB or other ethics committee submission, study protocol, or other planning document written before analyzing the data) was used in designing the study, please include the relevant document(s) with your revised manuscript as a Supporting Information file to be published alongside your study and cite it in the Methods section. A legend for this file should be included at the end of your manuscript. If no such document exists, please make sure that the Methods section transparently describes when analyses were planned, and when/why any data-driven changes to analyses took place. Changes in the analysis, including those made in response to peer review comments, should be identified as such in the Methods section of the paper, with rationale.

**Response:** We have added it in the Method section.

(Page 13, Lines 247-249) The study hypotheses and analytical approach were developed before the main statistical analyses were conducted, and the analyses were performed between February and April 2026. No changes to the analytical approach were made based on data-driven findings.

**Journal Requirements**

We have noticed that you have uploaded Supporting Information files, but you have not included a list of legends. Please add a full list of legends for your Supporting Information files after the references list.

**Response:** We have added a full list of legends.

Some material included in your submission may be copyrighted. According to PLOS’s copyright policy, authors who use figures or other material (e.g., graphics, clipart, maps) from another author or copyright holder must demonstrate or obtain permission to publish this material under the Creative Commons Attribution 4.0 International (CC BY 4.0) License used by PLOS journals. Please closely review the details of PLOS’s copyright requirements here: PLOS Licenses and Copyright. If you need to request permissions from a copyright holder, you may use PLOS's Copyright Content Permission form.

**Response:** Not applicable.

**[ Figure 1]:** Please confirm whether you drew the images / clip-art within the figure panels by hand. If you did not draw the images, please provide (a) a link to the source of the images or icons and their license / terms of use; or (b) written permission from the copyright holder to publish the images or icons under our CC BY 4.0 license. Alternatively, you may replace the images with open source alternatives.

**Response:** We have added the source of the images or icons and their license.

Icons were adapted from free icons available from Flaticon (https://www.flaticon.com/) and used in accordance with the Flaticon license with attribution.
