# Supplementary material for: Trajectories of physical function, depressive symptoms, and cognitive performance before and after falls in older adults: A matched multicohort study
Source: PLoS Med. 2026 Aug 3;23(8):e1005083. doi: 10.1371/journal.pmed.1005083 (PMC13446737; doi:10.1371/journal.pmed.1005083)
Supplement: Supplementary file 4 [file pmed.1005083.s004.docx]

**Manuscript ID:** PMEDICINE-D-26-01351R2

**Title:** Trajectories of physical function, depressive symptoms, and cognitive performance before and after falls in older adults: a matched multicohort study

Dear Dr Katarzyna Starowicz,

Thank you for your continued time and effort in reviewing our revised manuscript and for giving us the opportunity to further improve it through these minor revisions. We would like to thank the editors and reviewers for their thorough comments. We have considered the reviewer’s comments in the point-by-point reply. **Our response is given in the blue text** and changes/additions to the **manuscript are given in the red text**.

Dr. Xiaolin Xu, on behalf of all authors

**Response to the reviewers**

**Reviewer #1**

Thank you very much for carefully revising the manuscript! I know I had many comments on your manuscript, so I really appreciate the time and effort you put into the revisions. You did a great job addressing them.

I only have a few additional comments regarding your responses to my previous review (Reviewer 1). However, most of them concern language issues and should be relatively quick to address.

**Response:** Thank you very much for your valuable comments and for your careful evaluation of our revised manuscript. We have addressed your additional comments and revised the manuscript accordingly.

**Comment 1:** Both wordings still sound a bit unnatural to me. I would suggest something like "persons with a history of falls", "persons who experienced falls/experienced a fall".

**Response:** Thank you. We have revised the relevant wording throughout the manuscript to “participants/persons who experienced falls,” as appropriate.

**Comment 2:** I am still not entirely sure about the term preventable. While, in an ideal world, depression and cognitive impairment may be preventable, what seems more important is that severe consequences may be mitigated, and, for example, low mobility may be improved through strength training. Working on the risk factors mentioned in the cited studies may help to make falls and their consequences somewhat preventable.

**Response:** Thank you for your clarification. We removed the term “preventable” to ensure more precise and inclusive language and, following your suggestion, revised the sentence.

(Page 6, Lines 99-100) Addressing these factors may help reduce the risk of falls and	mitigate their adverse consequences[4,6].

**Comment 3:** There is some information missing in the sentence, because there is no pendant to the word either. If the studies analyze functional trajectories after a fall, all participants have a history of falls. Please revise the sentence. I would also suggest describing the samples of the cited studies to better contextualize how heterogeneous the populations are.

**Response:** Thank you for pointing this out. We recognize that the original wording may have caused confusion and have revised the sentence for clarity.

(Pages 6-7, Lines 109-1114) Emerging studies have examined functional trajectories after a	fall, either among individuals who experienced falls alone or by comparing their trajectories	with those of individuals who did not experience falls.

**Comment 4:** I would suggest using the term physical function and performance here and maybe the following manuscript because it is more neutral.

**Response:** We have replaced “physical disability” with “physical function” throughout the manuscript, including in the title.

**Comment 5:** How would you define regular mobility? I would suggest using terms such as strength and balance training. You could also highlight that regular assessments are important for identifying individuals at risk and implementing preventive measures, because otherwise risk factors may remain unidentified and individuals may remain untreated. Furthermore, I am not sure whether I would emphasize primary prevention, given that the aim of the study was to examine trajectories before and after falls. However, you could state that prevention should start as early as possible.

**Response:** We have revised this passage following your helpful suggestion.

(Pages 18-19, Lines369-373) In addition, individuals with less favorable trajectories may	have a greater burden of risk factors[11], underscoring the importance of initiating fall	prevention as early as possible and sustaining it after a fall. Regular assessments are	important for identifying individuals at risk and implementing timely preventive interventions,	such as strength, balance, and cognitive training[6,13], while continued post-fall	management may help reduce the risk of recurrent falls[2,30].

**Comment 6:** I think the point you make here is really important, especially regarding the need for tools that are feasible for implementation in clinical practice, as well as the importance of regular screening to detect early decline.

I would suggest specifying to which domain (cognitive, physical, or depression) the threshold of 0.25 refers.

Regarding the wording, "short" and "quick" are very similar terms, so I would suggest replacing one of them with "feasible." In addition, using "brief" and "rapid" in the same sentence seems somewhat repetitive. May I suggest the following wording: "Short assessments that are validated and feasible for clinical practice may help identify older adults at higher risk of falls." The Short Physical Performance Battery, for example, primarily assesses lower-extremity function, whereas e.g. measures such as gait speed or the Timed Up and Go test are also recommended. Therefore, I think the choice of assessment depends on the specific aspect one aims to evaluate. In addition, although screening tools can be rapid, they may have lower sensitivity or specificity. Therefore, I would also emphasize the importance of feasibility in your argument.

**Response:** Thank you for your positive comment and helpful suggestions. We have revised accordingly, and we have also clarified the selection of assessment instruments and specified the domain to which the 0.25-SD threshold refers.

(Page 20, Lines 401-406) Short assessments that are validated and feasible for clinical	practice may help identify older adults at higher risk of falls[2,4,6]. The choice of instrument	should depend on the specific functional domain being assessed. Examples include	questionnaires for depressive symptoms; gait speed, the Timed Up and Go test, or the Short	Physical Performance Battery for physical function; and validated cognitive screening	tools[2,4,6].

(Page 20, Lines 409-410) For example, our multi-trajectory modelling suggests that even	modest deterioration, **approximately 0.25 SD in any of the three domains relative to the	baseline distribution**, may be associated with higher odds of falls.

**General editorial requests:**

Please confirm that your title complies with PLOS Medicine's style. Your title must be nondeclarative and not a question. It should begin with main concept if possible. "Effect of" should be used only if causality can be inferred, i.e., for an RCT. Please place the study design ("A randomized controlled trial," "A retrospective study," "A modelling study," etc.) in the subtitle (ie, after a colon).

**Response:** We confirm that the title complies with PLOS Medicine’s style requirements.

Please confirm that your abstract complies with our requirements, including format (three sections: Background, Methods and Findings, and Conclusions) and providing all the information relevant to this study type https://journals.plos.org/plosmedicine/s/submission-guidelines#loc-abstract.

**Response:** We have confirmed that the abstract complies with the journal’s requirements.

Please ensure that the Introduction ends with a clear description of the study question or hypothesis.

**Response:** We have confirmed this.

Please ensure that all abbreviations are defined at first use throughout the text.

**Response:** We have confirmed this.

Please confirm that all numbers presented in the abstract are present and identical to numbers presented in the main manuscript text.

**Response:** Yes, we have confirmed this.

**GENERAL**

Please disclose any use of AI tools and technologies to the study or article content at the end of the Methods. Please specify the name(s) and version(s) of any tool(s) used and a description of their use, including how the AI-assisted or generated content was evaluated by authors, and a clear statement of which aspects of the study, article contents, data, or supporting files were affected/generated by AI tool usage. Please also include a statement specifying who retains responsibility for the final manuscript. See our guidelines for more information: https://journals.plos.org/plosmedicine/s/ethical-publishing-practice#loc-artificial-intelligence-tools-and-technologies.

**Response:** No AI tools or technologies were used in this study.

Please review your text for claims of novelty or primacy (e.g. 'for the first time') and remove this language. In addition, please check that any use of statistical terms (such as trend or significant) are supported by the data, and if not please remove them.

**Response:** We have reviewed the manuscript and confirmed that it contains no claims of novelty or primacy. We also verified that all statistical terms are supported by the data.

Statistical reporting: Please revise throughout the manuscript, including tables and figures.

- Please report statistical information as follows to improve clarity for the reader ""22% (95% CI [13,28]; p</=)"".

- Please separate upper and lower bounds with commas instead of hyphens as the latter can be confused with reporting of negative values.

- Please repeat statistical definitions (HR, CI etc.) for each set of parentheses."

**Response:** We have revised the statistical reporting throughout the manuscript, including the tables and figures, to follow the requested format.

In the last sentence of the Abstract Methods and Findings section, please describe the main limitation(s) of the study's methodology.

**Response:** We have added the main methodological limitations to the final sentence of the Abstract Methods and Findings section.

In the abstract, please include the important dependent variables that are adjusted for in the analyses.

**Response:** We have added the main covariates adjusted for in the analyses to the Abstract Methods and Findings section.

Thank you for providing Author Summary. In the author summary, in the final bullet point of 'What Do These Findings Mean?', please include the main limitations of the study in non-technical language. Please see our author guidelines for more information: https://journals.plos.org/plosmedicine/s/revising-your-manuscript#loc-author-summary."

**Response:** We have added the study limitations to the Author Summary.

**FUNDING STATEMENT**
The funding statement should include: specific grant numbers, initials of authors who received each award, URLs to sponsors’ websites. Also, please state whether any sponsors or funders (other than the named authors) played any role in study design, data collection and analysis, the decision to publish, or preparation of the manuscript. If they had no role in the research, include this sentence: “The funders had no role in study design, data collection and analysis, decision to publish, or preparation of the manuscript.”

**Response:** We have included all requested information and clarified that the funders had no role in the study.

**COMPETING INTERESTS STATEMENT**

All authors must declare their relevant competing interests per the PLOS policy, which can be seen here: https://journals.plos.org/plosmedicine/s/competing-interests For authors with ties to industry, please indicate whether any of the interests has a financial stake in the results of the current study.

Please amend your competing interests statement. Funding information should go to the relevant section and include URLs.

**Response:** We have amended the competing interests statement.

**DATA AVAILABILITY**
Thank you for agreeing to make your code available. I have checked URL and repository is empty. Please upload your materials. Along with Github link please include a citable digital object identifier (DOI) or other persistent identifier of the code (such as through linkage with Zenodo).

**Response:** Thank you for pointing this out. We have uploaded code to the GitHub repository and added the corresponding Zenodo DOI as a persistent identifier.

**ETHICS AND CONSENT**

Please revise for use of patient-centered language. Please note that patient-centered language is constructed with the use of post-modified nouns (e.g. 'patients with psoriasis’ (or similar) instead of ‘psoriasis patients’) putting the person first in the sentence structure.

**Response:** We have confirmed that the Ethics and Consent section and other relevant content comply with this requirement.

**FIGURES**
Please ensure that where relevant figures include 95% CIs.

When a p value is given, please specify the statistical test used to determine it in the legend.

Please provide the unadjusted comparisons as well as the adjusted comparisons in all relevant Tables.

**Response:** We have confirmed that the Ethics and Consent section and all other relevant sections comply with this requirement.

**ACKNOWLEDGMENTS**

Please include an acknowledgment of study participants.

**Response:** We have revised the Acknowledgements section.

**OBSERVATIONAL, COHORT, CROSS-SECTIONAL, AND CASE CONTROL STUDIES**

Thank you for providing STROBE Checklist. When completing the checklist, please use section and paragraph numbers, rather than page numbers – please revise.

Your study is observational and therefore causality cannot be inferred. Please remove language that implies causality and refer to associations instead.

**Response:** We have revised the STROBE Checklist to use section and paragraph numbers instead of page numbers. We have also reviewed the manuscript and revised any language implying causality to describe associations instead.
