# Supplementary material for: Trajectories of physical function, depressive symptoms, and cognitive performance before and after falls in older adults: A matched multicohort study
Source: PLoS Med. 2026 Aug 3;23(8):e1005083. doi: 10.1371/journal.pmed.1005083 (PMC13446737; doi:10.1371/journal.pmed.1005083)
Supplement: Peer review history

# Editor report: Decision Letter 0

**DOI:** 10.1371/journal.pmed.1005083.r001

**Authors:** Katarzyna Starowicz

21 Apr 2026

Dear Dr Xu,

Thank you for submitting your manuscript entitled "Trajectories of physical, psychological, and cognitive functioning before and after falls in older adults: a matched multicohort study" for consideration by PLOS Medicine.

Your manuscript has now been evaluated by the PLOS Medicine editorial staff, and I am writing to let you know that we would like to send your submission out for external peer review.

However, before we can send your manuscript to reviewers, we need you to complete your submission by providing the metadata that is required for full assessment. To this end, please login to Editorial Manager where you will find the paper in the 'Submissions Needing Revisions' folder on your homepage. Please click 'Revise Submission' from the Action Links and complete all additional questions in the submission questionnaire.

For clinical studies, please upload a copy of your trial study protocol as a supporting information file. The study protocol should be the version submitted for approval to the institutional review board or ethics committee, should include any amendments to the study protocol, as well as the date of their approval by the institutional review or ethics committee. Please also detail any deviations from the study protocol in the Methods section of your manuscript. The editors will consider the protocol and study conduct prior to a final decision for external review.

Please re-submit your manuscript within two working days, i.e. by Apr 23 2026 11:59PM.

Login to Editorial Manager here: https://www.editorialmanager.com/pmedicine

Once your full submission is complete, your paper will undergo a series of checks in preparation for peer review. Once your manuscript has passed all checks it will be sent out for review.

Feel free to email us at plosmedicine@plos.org if you have any queries relating to your submission.

Kind regards,

Katarzyna Starowicz, PhD

Associate Editor

PLOS Medicine

# Peer review: Decision Letter 1

**DOI:** 10.1371/journal.pmed.1005083.r002

**Authors:** Katarzyna Starowicz

28 May 2026

Dear Dr Xu,

Many thanks for submitting your manuscript "Trajectories of physical, psychological, and cognitive functioning before and after falls in older adults: a matched multicohort study" (PMEDICINE-D-26-01351R1) to PLOS Medicine. The paper has been reviewed by subject experts and a statistician; their comments are included below and can also be accessed here: [LINK]

As you will see, the reviewers and Academic Editor find your study interesting and important for the field. Nevertheless, they raised some concerns around methodology and results. After discussing the paper with the editorial team and an academic editor with relevant expertise, I'm pleased to invite you to revise the paper in response to the reviewers' comments. We plan to send the revised paper to some or all of the original reviewers, and we cannot provide any guarantees at this stage regarding publication.

Please note, after reviewers comments there are listed editorial general requests and study-type specific requests at the end of this letter. You must provide point-by-point response to each of them. Some of them might not be applicable to your study, in that case please respond "not applicable". Not providing this in form of rebuttal will significantly delay your manuscript.

In addition to these revisions, you may need to complete some formatting changes, which you will receive in a follow up email. A member of our team will be in touch with a set of requests shortly. If you do not receive a separate email within a few days, please assume that checks have been completed, and no additional changes are required.

When you upload your revision, please include a point-by-point response that addresses all of the reviewer and editorial points, indicating the changes made in the manuscript and either an excerpt of the revised text or the location (eg: page and line number) where each change can be found. Please also be sure to check the general editorial comments at the end of this letter and include these in your point-by-point response. When you resubmit your paper, please include a clean version of the paper as the main article file and a version with changes tracked as a marked-up manuscript. It may also be helpful to check the guidelines for revised papers at http://journals.plos.org/plosmedicine/s/revising-your-manuscript for any that apply to your paper.

We ask that you submit your revision by Jun 18 2026 11:59PM. However, if this deadline is not feasible, please contact me by email, and we can discuss a suitable alternative.

Don't hesitate to contact me directly with any questions (kstarowicz@plos.org).

Best regards,

Katarzyna

Katarzyna Starowicz, PhD

Associate Editor

PLOS Medicine

kstarowicz@plos.org

-----------------------------------------------------------

Comments from the academic editor:

This is a nicely written study that is of clinical interest and appears to fill a gap in the literature. The reviewers have picked up on a range of appropriate issue. One extra issue I would like to raise is the measurement of physical function. It is not really clear from the methods how it was measured as it does not appear that any of the cohorts used a validated measure. The authors should provide the actual questions and response items used in each cohort for greater clarity. The use of "difficulty controlling urination & defecation" in place of difficulty walking across the room in CHARLS does not seem appropriate to me and needs explanation. Do the results change if this item was omitted from the physical function score? I am not sure most people would agree that urinary/defecation control should be considered "physical" function and grouped with the other items "walking across a room, dressing, bathing, eating, getting in and out of bed, and using the toilet". perhaps the authors have taken "using the toilet" literally but in a physical function context that would usually mean how difficult is it to get on/off the toilet.... Comorbid conditions have been included as a covariate- but they have been included as crude "number of conditions" and thus assume each condition is equally as important/influential as the other. For example I would consider stroke to be potentially much more impactful on falls risk than diabetes. The authors should justify this approach and/or consider including key comorbidites separately, rather than simply as a crude "count"

-----------------------------------------------------------

Comments from the reviewers:

Reviewer #1: Review:

Thank you for the opportunity to review your manuscript! The results are very interesting and important for falls prevention. The implication that prevention should start as early as possible is particularly relevant. I have provided a detailed assessment; however, most of my comments mainly relate to the use of more precise and inclusive language.

Abstract and manuscript:

23 and the following: I would suggest a change in wording, as the term "fallers" is not appropriate. For further information, please see: Preferred language when referring to older people in a health context | British Geriatrics Society

35 fall onset: I would suggest changing the wording, as "onset" is not commonly used for falls but rather for diseases.

Introduction:

82 The description of activity restriction following a fall could be elaborated further. The cited report refers to restricted activity for only one day and does not specify the type of activity (e.g., social or physical). I would therefore suggest citing an alternative source, such as one addressing medical attention or reductions in physical activity.

82 Did you include hip fractures because they are the most common injury after a fall? This clarification would be important, particularly as fall severity is later operationalised using hip fractures rather than other injuries, such as traumatic brain injuries.

83 The term "psychological problems" is vague. Please specify the condition (e.g., depression) and consider using more precise and inclusive language. I would argue that the aforementioned risk factors are only partially preventable and would suggest softer wording.

88 You stated that many studies are of cross-sectional in nature; however, the study by Ek et al. that you cite analysed associations over a longer follow-up period. Similarly, the study by Jacob et al. assessed anxiety across two waves. I also believe, that they are quite a few studies which analyse trajectories. In the context of your rationale, I would suggest clarifying this sentence. You could mention that many studies assess trajectories either after a fall or in heterogeneous populations that include individuals with and without a history of falls, and that there appears to be a lack of studies examining the impact of a fall on changes in ongoing trajectories.

88- 90 I would split this sentence into two separate ones to improve readability and precision. In the second sentence, I would suggest explicitly mentioning the comparison group (e.g., individuals without a history of falls). For example: "Falls were associated with poorer ADLs compared to individuals without fall experience."

92 This association is also longitudinal. How does this reference strengthen your rationale?

93 The rationale represents an important research aim. You could further strengthen the introduction by highlighting that trajectories following a fall are relatively well studied, whereas comparisons of trajectories before and after a fall remain limited. Is there literature examining whether more severe falls are associated with changes in these trajectories?

96 The "s" is missing in "hip fracture" to indicate the plural

Methods:

109 no hyphen between individual and participant

110 Were these countries chosen because their data are most comparable? Are there differences in characteristics between these countries, for example in fall incidence?

120 To make the data more comparable, would it not have been possible to include all persons aged 65 years? Why did you decide against that?

147 Could you clarify why fall severity was stratified using these variables? Was this decision based on the availability of data for these factors? The World Falls Guidelines, for example, also suggest categories such as frailty and loss of consciousness after a fall.

154 I wonder whether there is an even better term than "psychological functioning", because cognitive function could also be considered part of psychological functioning, and because depression represents only a small part of psychological functioning.

178 Was there information on other conditions such as glaucoma, Parkinson's disease?

193 and following: fall-related conditions: what do you define as a fall-related conditions? I would suggest defining this better in the methods section.

200 why was the cut-off 7 years and not a shorter time period?

201 The entire sentence is bold, although only the headline should be.

Results:

229 I would suggest stating the origin of each database earlier. It may not be common knowledge that, for example, the Health and Retirement Study is a USA-based study.

250 I would suggest rewording this section. Recurrent falls should not be classified as a fall-related condition, and hip fractures are more appropriately described as injuries rather than conditions.

Figure 2: Very interesting!

Discussion:

314 Please see my comment on the introduction. The point you are making is strong; however, I would suggest a different argument, as there are many longitudinal studies, which you then mention a couple sentences later.

316 I would suggest placing the phrase "highlights a long window for prevention and intervention" at the end of the paragraph. This represents an important implication of the findings for future research and could be elaborated further. For example, individuals with less favourable trajectories may have more risk factors, suggesting that prevention requires sustained efforts. Interventions such as strength and balance training need to be done regularly. At the same time, the importance of secondary prevention could be emphasised to reduce the risk of subsequent falls.

338 It is possible that interventions targeting physical function may also benefit other domains. For example, physical activity may positively influence cognitive health, while group-based exercise and social interaction may contribute to overall well-being.

341 I would suggest using the term "concerns about falling" instead of "fear of falling" , because it may be perceived as less stigmatizing.

347 Perhaps you could place the following paragraph on implications before the future research paragraph. This would allow the future research paragraph to build on the implications.

348 How do you define simple assessments? Assessments that are short and quick? Maybe it would also be relevant that the screenings are regular to identify decline earlier? Can you define a cut-off of, based on this research, when decline is relevant and significant?

351 Not all older adults have a caregiver, and individuals are often responsible for managing their own health. It may therefore be more appropriate to refer to the individual and their support system, as well as the healthcare system.

364 This may also be relevant for falls. If a fall occurred a long time ago and had no severe consequences, it may not be recalled or reported. It appears that fall status prior to cohort entry was not assessed and information on that is not available, right? If so, the fall included in the analysis may not represent the first fall, and earlier falls may have already contributed to functional decline?

Would it be a limitation that slightly different questionnaires were used, for example for assessing depression?

370 What hypotheses do you have regarding the surgery data, particularly given its potential impact on fall severity?

375 Very important result!

378 I wonder whether the conclusion should instead be that risk assessment should be multifactorial, as also stated in the World Falls Guidelines.

Figure S1: I would suggest not using the word "Fall-free older adults" here. You use the wording multiple falls here. I think recurrent falls are often defined as >1 Fall in one year, which is not the definition you use in your paper. Nonetheless, I would suggest using the same wording for the paper and the figure.

Reviewer #2: Firstly, I would like to commend the authors on their recent submission which is indeed thorough. They show clear evidence of statistical considerations with subgroup and sensitivity analyses. The question posed is of importance, assessing falls and its impact on physical, psychological and cognitive functioning. I have some points below for your consideration. Please note, I recommended major revisions due to the potentially long computational time for modelling.

The methods should mention that all patients are community dwelling, it is reported as a limitation but I feel it should be clearly stated earlier.

Age was not considered during the matching process. In table S2 it shows the HRS cohort is older than the other two (74.9 vs 66.8 and 69.7 years). This also leads to a statistically significant difference in the matching for age. With those who fall being older, this is commonly known impact of ageing. I appreciate this is then utilised as a covariate in the model. Could the authors please clarify their reasoning for this? Do they consider age to be a predictive factor, similar to number of comorbidities, and have included due to this? If so, could the authors please clarify why they chose the factors they did for matching and those they did not. Additionally, as age is a significant factor for falls, were there more falls in the HRS cohort than the other cohorts? Number of falls data is not presented but is an outcome. I appreciate multiple falls is not available for one cohort (CHARLS).

With regards to comorbidities, was data available for those who had neurological conditions such as Parkinson disease? If so, can the authors provide justification for not considering those patients? Neurological conditions do not appear to be part of the eligibility criteria.

For the analyses of separate trajectories, linear mixed effect models are utilised, however, the figures suggest that linearity does not hold. I would recommend a sensitivity analysis using non-linear methods, the piecewise model could be applied in a non-linear manner. You may also wish to consider other models such as fractional polynomials or spline models, if not, provide justification.

Reviewer #3: The manuscript addresses an important and timely topic concerning changes in physical, psychological, and cognitive functioning before and after falls in older adults. A major strength of the study is the use of data from three large longitudinal cohorts and the multidomain approach to functional trajectories.

The findings suggesting that functional decline begins several years before a fall and continues afterward are interesting and may have important clinical and public health implications.

However, several issues should be addressed before publication.

1. The interpretation of the findings is sometimes too strong. Since this is an observational study, it is difficult to determine whether declines in functioning are a consequence of falls or whether poorer functioning increases the risk of falling. The conclusions should therefore be more cautious.

2. Falls were assessed using self-reported data, which may introduce recall bias and underreporting. This limitation should be discussed in more detail.

3. Matching was performed only for age, sex, and cohort. Despite matching, differences between groups remained in physical activity and comorbidities, suggesting possible residual confounding.

4. Although harmonization across CHARLS, ELSA, and HRS is an important strength, differences in measurements and assessment tools between cohorts may still affect comparability of the results. This issue deserves further discussion.

5. Some observed associations, particularly those related to cognitive functioning, appear relatively small. It would be helpful to discuss whether these differences are clinically meaningful.

6. Survivor bias may also have influenced the findings, especially among participants with severe falls or hip fractures.

Overall, this is an interesting and potentially valuable study with important implications for geriatric and public health research. However, the manuscript would benefit from a more cautious interpretation of the findings and a clearer discussion of methodological limitations.

Reviewer #4: The authors investigated into the trajectories of matched fallers and non-fallers in the physical, psychological and cognitive function using data of three well known Longitudinal studies (UK, China, and USA). The trajectories of a longer period of time is making this manuscript very valuable, and will add further evidence to the current knowledge in the fall prevention area. The manuscript is already in good shape but some major comments needed to be addressed before it can be considered for publication. The reviewer has really enjoyed reading this manuscript.

Title:No comments

Abstract: may be the authors should consider to also include the longitudinal aspect of these trajectories (which is an asset of this manuscript) into the background.

key words: For the reviewer the key word of "hip fracture" is not directly related but more important are information on the setting of the cohort.

Authors summary: well written no comments.

Introduction:

The authors are leading very good into their research topic. They are citing the WHO Step safely and other important recommendations. Nevertheless, the reviewer would suggest to insert on page 6 line 78-85 also a quick introduction into the four domains provided by the WHO Framework "Step Safely".

In Line 86 it would be useful to insert an explanation of which variables in the Physical psychological and cognitive domain the authors are taking into account. As for the reviewer the domain of cognition would normally be a subcategory of a psychological domain.

The objectives are very well stated at the end of the introduction.

Methods:

For the reviewer the following statement it is not clear: "available data on physical, psychological, and cognitive functioning in at least one wave" (p 8 line 124-125), as the authors investigated also the pre-post trajectories and this would include at least two assessment times. Please explain.

the classification and categorization process is well described.

Results:

As the authors categorized different faller groups (multiple faller, hip fracture faller) a short differentiation in line (p 13 line 226-236) should be added, as just the term "fallers" is being too broad. this aspect is later being shortly mentioned (same page line 239-240) but would be need in the short summary of results as stated above.

the section p 14 line 250 to 266 is really hard to read, and authors should consider reviewing it or presenting it in a table for more readability.

Discussion:

Already well written and in good shape.

One aspect which the author might consider: the ADL measures are a very rough instrument of physical function and impairments in this area are probably more a sign of frailty (directing to a very vulnerable population), and might be therefore a bias of a predictor of falls.

another aspect the authors might consider is that all the harmonization (z-scores, and categorization) valuable information may have been lost (just a short sentence acknowledge will be fine).

Any attachments provided with reviews can be seen via the following link: [LINK]

--------------------------------------------------------- ---

General editorial requests:

(Note: not all will apply to your paper, but please check each item carefully)

*We ask every co-author listed on the manuscript to fill in a contributing author statement, making sure to declare all competing interests. If any of the co-authors have not filled in the statement, we will remind them to do so when the paper is revised. If all statements are not completed in a timely fashion this could hold up the re-review process. If new competing interests are declared later in the revision process, this may also hold up the submission. Should there be a problem getting one of your co-authors to fill in a statement we will be in contact. Please do not add or remove authors without first discussing this with the handling editor. You can see our competing interests policy here: http://journals.plos.org/plosmedicine/s/competing-interests.

*Please upload any figures associated with your paper as individual TIF or EPS files with 300dpi resolution at resubmission; please read our figure guidelines for more information on our requirements: http://journals.plos.org/plosmedicine/s/figures. While revising your submission, we strongly recommend that you use PLOS's NAAS tool (https://ngplosjournals.pagemajik.ai/artanalysis) to test your figure files. NAAS can convert your figure files to the TIFF file type and meet basic requirements (such as print size, resolution), or provide you with a report on issues that do not meet our requirements and that NAAS cannot fix.

After uploading your figures to PLOS's NAAS tool - https://ngplosjournals.pagemajik.ai/artanalysis, NAAS will process the files provided and display the results in the "Uploaded Files" section of the page as the processing is complete.

If the uploaded figures meet our requirements (or NAAS is able to fix the files to meet our requirements), the figure will be marked as "fixed" above. If NAAS is unable to fix the files, a red "failed" label will appear above.

When NAAS has confirmed that the figure files meet our requirements, please download the file via the download option, and include these NAAS processed figure files when submitting your revised manuscript.

*Please ensure that the paper adheres to the PLOS Data Availability Policy (see http://journals.plos.org/plosmedicine/s/data-availability), which requires that all data underlying the study's findings be provided in a repository or as Supporting Information. For data residing with a third party, authors are required to provide instructions with contact information (web or email address) for obtaining the data. Please note that a study author cannot be the contact person for the data. PLOS journals do not allow statements supported by "data not shown" or "unpublished results." For such statements, authors must provide supporting data or cite public sources that include it.

*We expect all researchers with submissions to PLOS in which author-generated code underpins the findings in the manuscript to make all author-generated code available without restrictions upon publication of the work. In cases where code is central to the manuscript, we require the code to be made available as a condition of publication. Authors are responsible for ensuring that the code is reusable and well documented. Please make any custom code available, either as part of your data deposition or as a supplementary file. Please add a sentence to your data availability statement regarding any code used in the study, e.g. "The code used in the analysis is available from Github [URL] and archived in Zenodo [DOI link]" Please review our guidelines at https://journals.plos.org/plosmedicine/s/materials-software-and-code-sharing and ensure that your code is shared in a way that follows best practice and facilitates reproducibility and reuse. Because Github depositions can be readily changed or deleted, we encourage you to make a permanent DOI'd copy (e.g. in Zenodo) and provide the URL.

*Please ensure that the study is reported according to the STROBE guideline. When completing the checklist, please use section and paragraph numbers, rather than page numbers. Please add the following statement, or similar, to the Methods: "This study is reported as per STROBE guideline (S1 Checklist)."

FORMATTING - GENERAL

*Abstract: Please structure your abstract using the PLOS Medicine headings (Background, Methods and Findings, Conclusions). Please combine the Methods and Findings sections into one section.

*At this stage, we ask that you include a short, non-technical Author Summary of your research to make findings accessible to a wide audience that includes both scientists and non-scientists. The Author Summary should immediately follow the Abstract in your revised manuscript. This text is subject to editorial change and should be distinct from the scientific abstract. Ideally each sub-heading should contain 2-3 single sentence, concise bullet points containing the most salient points from your study. In the final bullet point of 'What Do These Findings Mean?', please include the main limitations of the study in non-technical language. Please see our author guidelines for more information: https://journals.plos.org/plosmedicine/s/revising-your-manuscript#loc-author-summary.

*Please express the main results with 95% CIs as well as p values. When reporting p values please report as p<0.001 and where higher as the exact p value p=0.002, for example. Throughout, suggest reporting statistical information as follows to improve clarity for the reader "22% (95% CI [13%,28%]; p</=)". Please be sure to define all numerical values at first use.

*Please include page numbers and line numbers in the manuscript file. Use continuous line numbers (do not restart the numbering on each page).

*Please cite the reference numbers in square brackets. Citations should precede punctuation.

FIGURES AND TABLES

*Please provide titles and legends for all figures and tables (including those in Supporting Information files).

*Please define all abbreviations used in each figure/table (including those in Supporting Information files).

*Please consider avoiding the use of red and green in order to make your figure more accessible to those with color blindness.

SUPPLEMENTARY MATERIAL

*Please note that supplementary material will be posted as supplied by the authors. Therefore, please amend it according to the relevant comments outlined here.

*Please cite your Supporting Information as outlined here: https://journals.plos.org/plosmedicine/s/supporting-information

REFERENCES

*PLOS uses the numbered citation (citation-sequence) method and first six authors, et al.

*Please ensure that journal name abbreviations match those found in the National Center for Biotechnology Information (NCBI) databases (http://www.ncbi.nlm.nih.gov/nlmcatalog/journals), and are appropriately formatted and capitalised.

*Where website addresses are cited, please include the complete URL and specify the date of access (e.g. [accessed: 12/06/2023]).

*Please also see https://journals.plos.org/plosmedicine/s/submission-guidelines#loc-references for further details on reference formatting.

STUDY TYPE-SPECIFIC REQUESTS - not all might apply

OBSERVATIONAL STUDIES

*Abstract: Please include the study design, population and setting, number of participants, years during which the study took place (enrollment and follow up), length of follow up, and main outcome measures.

*Please ensure that the study is reported according to the STROBE (or appropriate STOBE extension) guideline (available from: https://www.equator-network.org/reporting-guidelines/strobe) and include the completed STROBE (or STROBE extension) checklist as Supporting Information. Please add the following statement, or similar, to the Methods: "This study is reported as per the Strengthening the Reporting of Observational Studies in Epidemiology (STROBE) guideline (S1 Checklist)." When completing the checklist, please use section and paragraph numbers, rather than page numbers.

*[FOR MEDIATION ANALYSES] We recommend that the study is reported according to the AGReMA statement (https://agrema-statement.org/#:~:text=AGReMA%20is%20an%20evidence%2D%20and,randomised%20trials%20and%20observational%20studies) and include the completed checklist as Supporting Information. Please add the following statement, or similar, to the Methods: "This study is reported as per the Guideline for Reporting Mediation Analyses (AGReMA) statement (S1 Checklist)." When completing the checklist, please use section and paragraph numbers, rather than page numbers.

*For all observational studies, in the manuscript text, please indicate: (1) the specific hypotheses you intended to test, (2) the analytical methods by which you planned to test them, (3) the analyses you actually performed, and (4) when reported analyses differ from those that were planned, transparent explanations for differences that affect the reliability of the study's results. If a reported analysis was performed based on an interesting but unanticipated pattern in the data, please be clear that the analysis was data driven.

*Please state in the Methods section whether the study had a prospective protocol or analysis plan. If a prospective analysis plan (from your funding proposal, IRB or other ethics committee submission, study protocol, or other planning document written before analyzing the data) was used in designing the study, please include the relevant document(s) with your revised manuscript as a Supporting Information file to be published alongside your study and cite it in the Methods section. A legend for this file should be included at the end of your manuscript. If no such document exists, please make sure that the Methods section transparently describes when analyses were planned, and when/why any data-driven changes to analyses took place. Changes in the analysis, including those made in response to peer review comments, should be identified as such in the Methods section of the paper, with rationale.

# Author comment: Author response to Decision Letter 2

**DOI:** 10.1371/journal.pmed.1005083.r003

17 Jun 2026

Attachment Submitted filename: Point-to-point response letter.docx

# Peer review: Decision Letter 2

**DOI:** 10.1371/journal.pmed.1005083.r004

**Authors:** Katarzyna Starowicz

17 Jul 2026

Dear Dr. Xu,

Thank you very much for re-submitting your manuscript "Trajectories of physical disability, depressive symptoms, and cognitive functioning before and after falls in older adults: a matched multicohort study" (PMEDICINE-D-26-01351R2) for review by PLOS Medicine.

I have discussed the paper with my colleagues and the academic editor and it was also seen again by three reviewers. I am pleased to say that provided the remaining reviewer, editorial and production issues are dealt with we are planning to accept the paper for publication in the journal.

The remaining issues that need to be addressed are listed at the end of this email. As previously please provide rebuttal to ALL editorial and reviewers comments.

Any accompanying reviewer attachments can be seen via the link below. Please take these into account before resubmitting your manuscript:

[LINK]

***Please note while forming your response, if your article is accepted, you may have the opportunity to make the peer review history publicly available. The record will include editor decision letters (with reviews) and your responses to reviewer comments. If eligible, we will contact you to opt in or out.***

In revising the manuscript for further consideration here, please ensure you address the specific points made by each reviewer and the editors. In your rebuttal letter you should indicate your response to the reviewers' and editors' comments and the changes you have made in the manuscript. Please submit a clean version of the paper as the main article file. A version with changes marked must also be uploaded as a marked up manuscript file.

Please also check the guidelines for revised papers at http://journals.plos.org/plosmedicine/s/revising-your-manuscript for any that apply to your paper. If you haven't already, we ask that you provide a short, non-technical Author Summary of your research to make findings accessible to a wide audience that includes both scientists and non-scientists. The Author Summary should immediately follow the Abstract in your revised manuscript. This text is subject to editorial change and should be distinct from the scientific abstract.

In addition to these revisions, you may need to complete some formatting changes, which you will receive in a follow up email. A member of our team will be in touch with a set of requests shortly. If you do not receive a separate email within a few days, please assume that checks have been completed, and no additional changes are required.

We expect to receive your revised manuscript within 1 week. Please email us (plosmedicine@plos.org) if you have any questions or concerns.

We ask every co-author listed on the manuscript to fill in a contributing author statement. If any of the co-authors have not filled in the statement, we will remind them to do so when the paper is revised. If all statements are not completed in a timely fashion this could hold up the re-review process. Should there be a problem getting one of your co-authors to fill in a statement we will be in contact. YOU MUST NOT ADD OR REMOVE AUTHORS UNLESS YOU HAVE ALERTED THE EDITOR HANDLING THE MANUSCRIPT TO THE CHANGE AND THEY SPECIFICALLY HAVE AGREED TO IT.

Please ensure that the paper adheres to the PLOS Data Availability Policy (see http://journals.plos.org/plosmedicine/s/data-availability), which requires that all data underlying the study's findings be provided in a repository or as Supporting Information. For data residing with a third party, authors are required to provide instructions with contact information for obtaining the data. PLOS journals do not allow statements supported by "data not shown" or "unpublished results." For such statements, authors must provide supporting data or cite public sources that include it.

To enhance the reproducibility of your results, we recommend that you deposit your laboratory protocols in protocols.io, where a protocol can be assigned its own identifier (DOI) such that it can be cited independently in the future. Additionally, PLOS ONE offers an option to publish peer-reviewed clinical study protocols. Read more information on sharing protocols at https://plos.org/protocols?utm_medium=editorial-email&utm_source=authorletters&utm_campaign=protocols

Please review your reference list to ensure that it is complete and correct. If you have cited papers that have been retracted, please include the rationale for doing so in the manuscript text, or remove these references and replace them with relevant current references. Any changes to the reference list should be mentioned in the rebuttal letter that accompanies your revised manuscript.

Please note, when your manuscript is accepted, an uncorrected proof of your manuscript will be published online ahead of the final version, unless you've already opted out via the online submission form. If, for any reason, you do not want an earlier version of your manuscript published online or are unsure if you have already indicated as such, please let the journal staff know immediately at plosmedicine@plos.org.

If you have any questions in the meantime, please contact me or the journal staff on plosmedicine@plos.org.

We look forward to receiving the revised manuscript by Jul 24 2026 11:59PM.

Sincerely,

Katarzyna Starowicz, PhD

Associate Editor

PLOS Medicine

kstarowicz@plos.org

------------------------------------------------------------

Requests from Editors:

GENERAL EDITORIAL REQUESTS

* Please confirm that your title complies with PLOS Medicine's style. Your title must be nondeclarative and not a question. It should begin with main concept if possible. "Effect of" should be used only if causality can be inferred, i.e., for an RCT. Please place the study design ("A randomized controlled trial," "A retrospective study," "A modelling study," etc.) in the subtitle (ie, after a colon).

* Please confirm that your abstract complies with our requirements, including format (three sections: Background, Methods and Findings, and Conclusions) and providing all the information relevant to this study type https://journals.plos.org/plosmedicine/s/submission-guidelines#loc-abstract

* Please ensure that the Introduction ends with a clear description of the study question or hypothesis.

* Please ensure that all abbreviations are defined at first use throughout the text.

* Please confirm that all numbers presented in the abstract are present and identical to numbers presented in the main manuscript text.

GENERAL

* Please disclose any use of AI tools and technologies to the study or article content at the end of the Methods. Please specify the name(s) and version(s) of any tool(s) used and a description of their use, including how the AI-assisted or generated content was evaluated by authors, and a clear statement of which aspects of the study, article contents, data, or supporting files were affected/generated by AI tool usage. Please also include a statement specifying who retains responsibility for the final manuscript. See our guidelines for more information: https://journals.plos.org/plosmedicine/s/ethical-publishing-practice#loc-artificial-intelligence-tools-and-technologies.

* Please review your text for claims of novelty or primacy (e.g. 'for the first time') and remove this language. In addition, please check that any use of statistical terms (such as trend or significant) are supported by the data, and if not please remove them.

* Statistical reporting: Please revise throughout the manuscript, including tables and figures.

- Please report statistical information as follows to improve clarity for the reader ""22% (95% CI [13,28]; p</=)"".

- Please separate upper and lower bounds with commas instead of hyphens as the latter can be confused with reporting of negative values.

- Please repeat statistical definitions (HR, CI etc.) for each set of parentheses."

* In the last sentence of the Abstract Methods and Findings section, please describe the main limitation(s) of the study's methodology.

* In the abstract, please include the important dependent variables that are adjusted for in the analyses.

* Thank you for providing Author Summary. In the author summary, in the final bullet point of 'What Do These Findings Mean?', please include the main limitations of the study in non-technical language. Please see our author guidelines for more information: https://journals.plos.org/plosmedicine/s/revising-your-manuscript#loc-author-summary."

FUNDING STATEMENT

* The funding statement should include: specific grant numbers, initials of authors who received each award, URLs to sponsors’ websites. Also, please state whether any sponsors or funders (other than the named authors) played any role in study design, data collection and analysis, the decision to publish, or preparation of the manuscript. If they had no role in the research, include this sentence: “The funders had no role in study design, data collection and analysis, decision to publish, or preparation of the manuscript.”

COMPETING INTERESTS STATEMENT

* All authors must declare their relevant competing interests per the PLOS policy, which can be seen here: https://journals.plos.org/plosmedicine/s/competing-interests For authors with ties to industry, please indicate whether any of the interests has a financial stake in the results of the current study.

*Please amend your competing interests statement. Funding information should go to the relevant section and include URLs.

DATA AVAILABILITY

* Thank you for agreeing to make your code available. I have checked URL and repository is empty. Please upload your materials. Along with Github link please include a citable digital object identifier (DOI) or other persistent identifier of the code (such as through linkage with Zenodo).

ETHICS AND CONSENT

* Please revise for use of patient-centered language. Please note that patient-centered language is constructed with the use of post-modified nouns (e.g. 'patients with psoriasis’ (or similar) instead of ‘psoriasis patients’) putting the person first in the sentence structure.

FIGURES

* Please ensure that where relevant figures include 95% CIs.

* When a p value is given, please specify the statistical test used to determine it in the legend.

* Please provide the unadjusted comparisons as well as the adjusted comparisons in all relevant Tables

ACKNOWLEDGMENTS

* Please include an acknowledgment of study participants.

OBSERVATIONAL, COHORT, CROSS-SECTIONAL, AND CASE CONTROL STUDIES

* Thank you for providing STROBE Checklist. When completing the checklist, please use section and paragraph numbers, rather than page numbers – please revise.

* Your study is observational and therefore causality cannot be inferred. Please remove language that implies causality and refer to associations instead.

------------------------------------------------------------

Comments from Reviewers:

Reviewer #1: Thank you very much for carefully revising the manuscript! I know I had many comments on your manuscript, so I really appreciate the time and effort you put into the revisions. You did a great job addressing them.

I only have a few additional comments regarding your responses to my previous review (Reviewer 1). However, most of them concern language issues and should be relatively quick to address.

Comment 1:

Both wordings still sound a bit unnatural to me. I would suggest something like "persons with a history of falls", "persons who experienced falls/experienced a fall".

Comment 5:

I am still not entirely sure about the term preventable. While, in an ideal world, depression and cognitive impairment may be preventable, what seems more important is that severe consequences may be mitigated, and, for example, low mobility may be improved through strength training. Working on the risk factors mentioned in the cited studies may help to make falls and their consequences somewhat preventable.

Comment 6 Revisions Page 6 Lines 101-102:

There is some information missing in the sentence, because there is no pendant to the word either. If the studies analyze functional trajectories after a fall, all participants have a history of falls. Please revise the sentence. I would also suggest describing the samples of the cited studies to better contextualize how heterogeneous the populations are.

Line 103: I would suggest using the term physical function and performance here and maybe the following manuscript because it is more neutral.

Comment 24:

How would you define regular mobility? I would suggest using terms such as strength and balance training. You could also highlight that regular assessments are important for identifying individuals at risk and implementing preventive measures, because otherwise risk factors may remain unidentified and individuals may remain untreated. Furthermore, I am not sure whether I would emphasize primary prevention, given that the aim of the study was to examine trajectories before and after falls. However, you could state that prevention should start as early as possible.

Comment 28:

I think the point you make here is really important, especially regarding the need for tools that are feasible for implementation in clinical practice, as well as the importance of regular screening to detect early decline.

I would suggest specifying to which domain (cognitive, physical, or depression) the threshold of 0.25 refers.

Regarding the wording, "short" and "quick" are very similar terms, so I would suggest replacing one of them with "feasible." In addition, using "brief" and "rapid" in the same sentence seems somewhat repetitive. May I suggest the following wording: "Short assessments that are validated and feasible for clinical practice may help identify older adults at higher risk of falls." The Short Physical Performance Battery, for example, primarily assesses lower-extremity function, whereas e.g. measures such as gait speed or the Timed Up and Go test are also recommended. Therefore, I think the choice of assessment depends on the specific aspect one aims to evaluate. In addition, although screening tools can be rapid, they may have lower sensitivity or specificity. Therefore, I would also emphasize the importance of feasibility in your argument.

Reviewer #2: The authors have taken the comments made on board and made changes which have improved the paper. The non-linear analysis shows consistency between the results.

Reviewer #4: The reviewer wants to congratulate the authors for all their effort and hard work on improving the manuscript.

There are no more comments made by this reviewer.

Any attachments provided with reviews can be seen via the following link:

[LINK]

# Author comment: Author response to Decision Letter 3

**DOI:** 10.1371/journal.pmed.1005083.r005

20 Jul 2026

Attachment Submitted filename: Point-to-point_response_letter_auresp_3.docx

# Editor report: Decision Letter 3

**DOI:** 10.1371/journal.pmed.1005083.r006

**Authors:** Katarzyna Starowicz

24 Jul 2026

Dear Dr Xu,

On behalf of my colleagues and the Academic Editor, Rana Hinman, I am pleased to inform you that we have agreed to publish your manuscript "Trajectories of physical function, depressive symptoms, and cognitive performance before and after falls in older adults: a matched multicohort study" (PMEDICINE-D-26-01351R3) in PLOS Medicine.

Before your manuscript can be formally accepted you will need to complete some formatting changes, which you will receive in a follow up email. Please be aware that it may take several days for you to receive this email; during this time no action is required by you. Once you have received these formatting requests, please note that your manuscript will not be scheduled for publication until you have made the required changes.

In the meantime, please log into Editorial Manager at http://www.editorialmanager.com/pmedicine/, click the "Update My Information" link at the top of the page, and update your user information to ensure an efficient production process.

PRESS

We frequently collaborate with press offices. If your institution or institutions have a press office, please notify them about your upcoming paper at this point, to enable them to help maximise its impact. If the press office is planning to promote your findings, we would be grateful if they could coordinate with medicinepress@plos.org. If you have not yet opted out of the early version process, we ask that you notify us immediately of any press plans so that we may do so on your behalf.

We also ask that you take this opportunity to read our Embargo Policy regarding the discussion, promotion and media coverage of work that is yet to be published by PLOS. As your manuscript is not yet published, it is bound by the conditions of our Embargo Policy. Please be aware that this policy is in place both to ensure that any press coverage of your article is fully substantiated and to provide a direct link between such coverage and the published work. For full details of our Embargo Policy, please visit http://www.plos.org/about/media-inquiries/embargo-policy/.

To enhance the reproducibility of your results, we recommend that you deposit your laboratory protocols in protocols.io, where a protocol can be assigned its own identifier (DOI) such that it can be cited independently in the future. Additionally, PLOS ONE offers an option to publish peer-reviewed clinical study protocols. Read more information on sharing protocols at https://plos.org/protocols?utm_medium=editorial-email&utm_source=authorletters&utm_campaign=protocols

Thank you again for submitting to PLOS Medicine. We look forward to publishing your paper.

Sincerely,

Katarzyna Starowicz, PhD

Associate Editor

PLOS Medicine
